# The redescription of the holotype of *Nothosaurus mirabilis* (Diapsida, Eosauropterygia)—a historical skeleton from the Muschelkalk (Middle Triassic, Anisian) near Bayreuth (southern Germany)

**DOI:** 10.7717/peerj.13818

**Published:** 2022-08-26

**Authors:** Nicole Klein, Stefan Eggmaier, Hans Hagdorn

**Affiliations:** 1 University of Bonn, Institute of Geosciences, Paleontology, Bonn, Germany; 2 Urwelt-Museum Oberfranken, Bayreuth, Germany; 3 Muschelkalkmuseum Ingelfingen, Ingelfingen, Germany

**Keywords:** Historical paleontology, Upper Muschelkalk, Marine reptiles, Morphology, Locomotion, Axial skeleton, Lithological sections

## Abstract

In 2009, the historical mount of the holotype of *Nothosaurus mirabilis* from the Upper Muschelkalk of Oschenberg (Laineck Mountain Range, near Bayreuth, southern Germany) was disassembled and the original postcranial skeleton was reworked and remounted in find position. Its morphology is described and figured for the first time in detail. Further on, a thorough overview of the sedimentary environment and the historical activities around the Upper Muschelkalk quarries in the vicinity of Bayreuth is given. The holotype of *N. mirabilis* is one out of only two fairly complete nothosaur skeletons known from the Bayreuth Upper Muschelkalk and greatly emends our knowledge of the morphology of the species and the genus. It will further allow an assignment of isolated elements to this taxon. The specimen consists of an articulated and complete neck and anterior trunk vertebral column as well as several articulated parts of the anterior tail region. The sacral region is partially preserved but disarticulated. Besides vertebrae, ribs and gastral fragments, both humeri, the right femur, few zeugopodial and autopodial elements, and the right pelvic girdle are preserved. The very high neural spines of the holotype are stabilized by a supersized zygosphene-zygantrum articulation reaching far dorsally. Together with the large intercentral spaces this character suggests lateral undulation of the trunk region during fast swimming whereas propelling with the broad and wing-shaped humerus and the flat ulna was used during slower swimming. The total body length for this not fully grown individual is reconstructed as between 290 to 320 cm. Preservation, degree of completeness, and articulation of the individual is unique. The skull and shoulder girdle are both lost, whereas articulated strings of the vertebral column have turned and appendicular bones have shifted posteriorly or anteriorly, respectively, indicating water movements and possibly also scavenging.

## Introduction

### Nothosaurus

*Nothosaurus* is a member of Sauropterygia, a diverse group of secondarily adapted marine reptiles that existed from the late Early Triassic until the end of the Cretaceous. Sauropterygia appeared in the Early Triassic after the recovery of the Permo-Triassic extinction event in the eastern and western Tethys as well as in the Pacific realm (Li & Liu, 2020; Scheyer, Neuman & Brinkman, 2019). The genus *Nothosaurus* existed during the Middle Triassic (mainly Anisian to Ladinian; one species is known from the early Carnian). Isolated bones of Sauropterygia, and especially nothosaurs, occurred in high individual numbers in the fossil record of the Germanic Basin (Muschelkalk, Lower Keuper and basal Middle Keuper; *e.g*., Meyer, 1847–1855, Rieppel, 2000; Klein et al., 2015). The group is also represented in the Alpine Triassic (summarized in Rieppel, 2000), *i.e*., in the western Tethyan realm and is well known from the eastern Tethyan realm (*e.g*., Sun et al., 2016; Li & Liu, 2020). Evidence of Sauropterygia outside the Paleotethys is rare (*e.g*., Storrs, 1991; Rieppel, Sander & Storrs, 1997; Scheyer, Neuman & Brinkman, 2019).

Contrary to numerous finds of complete skeletons of sauropterygians from localities in China and from the Alpine Triassic, the Germanic Basin has mainly yielded isolated postcranial elements and isolated skulls (black-shale *vs*. bonebed preservation). Except for the middle Anisian (Lower Muschelkalk) locality of Winterswijk (Klein et al., 2015; Voeten, Albers & Klein, 2019), the entire Muschelkalk deposits have only produced a handful of partially articulated sauropterygian skeletons (Rieppel, 2000). Aside from Winterswijk, only three at least partially articulated *Nothosaurus* skeletons (*vs*. countless isolated bones) are described from other Muschelkalk localities, two of which are Upper Muschelkalk (in the following abbreviated as UM): one is the here redescribed holotype of *Nothosaurus mirabilis* (postcranium; Münster, 1834; Meyer, 1847–1855; Rieppel & Wild, 1996) and the other is the anterior half of a small skeleton with the skull and lower jaw *in situ* (*Nothosaurus jagisteus*; Rieppel, 2001).

Within Sauropterygia, the classical Nothosauroidea (*Nothosaurus*, *Lariosaurus, Simosaurus*) as established by Rieppel (2000) comprised together with Pistosauroidea, the Eusauropterygia. Eusauropterygia and Pachypleurosauria formed the order Eosauropterygia (Rieppel, 2000). However, recent phylogenetic analyses, including numerous new taxa mainly described from China, have now questioned the traditional phylogenetic relationships of Nothosauroidea and Eosauropterygia, respectively (*e.g*., Liu et al., 2014; Li & Liu, 2020; Lin et al., 2021; Shang, Wu & Li, 2020). Until further results have clarified exact phylogenetic relationships, we use the terms here in the traditional meaning *sensu* Rieppel (2000). For a more detailed overview of the stratigraphical and geographical distribution of *Nothosaurus* and species included see Hagdorn & Rieppel (1999) and Voeten, Albers & Klein (2019).

In addition to the unclear phylogenetic in-group relationships, the ancestry and origin of Sauropterygia is unknown. Among other problems, the isolated nature of finds from the Germanic Basin hampers alpha taxonomy and comparison with taxa from other realms. These unresolved questions around phylogenetic relationships, ancestry, and origin as well as the rareness of articulated material from the Muschelkalk, emphasize the relevance of any articulated postcranial material from Muschelkalk localities.

### The Upper Muschelkalk of Bayreuth

Paleontological activities around the Bayreuth quarries, most of which are no longer accessible, have a history spanning 220 years nicely providing insights into the beginning of paleontology in Germany. Further on, the quarries in the vicinity of Bayreuth had been the most productive area of the late Anisian UM marine reptiles in Germany. Today most of the fossils from there are spread over different collections. In the following discussion we want to share and clarify information otherwise only available in scattered publications in German language. However, some of these anecdotal historical statements may be contradicting. This summary covers the history of collecting as well as the early research and exhibition of specimens, lithological sections of the most important quarries, and an overview of the Bayreuth marine reptile fauna.

#### Historical overview

Among paleontologists working on Triassic vertebrates, the town of Bayreuth (South Germany, Bavaria, Upper Franconia) and its surroundings is famous as type region of key genera of Triassic marine reptiles (summarized in Rieppel (2000)). First and foremost, this is due to the activities of Count Georg Graf zu Münster (1776–1844). Since 1806 he was ‘Kriegs- und Domänenrat’ (member of the administration) of district Oberfranken (Upper Franconia, Bavaria) in Bayreuth. His interest in natural history and his occupation enabled him to assemble the largest fossil collection in the first half of the 19th century in Germany, which later after his death became the base of the Bavarian State Collection of Paleontology and Geology (Weiss, 1937, 1983; Müller, 1979; Wild, 1988–1989). Since 1809 the Muschelkalk quarries in Bayreuth’s immediate vicinity became one of Münster’s major collecting areas yielding ‘bones of huge turtles, plesiosaurs and other still unknown antemundane reptiles, teeth, bones, and scales of fishes of several very different genera, among which some are distinguished by size, shape, and color’ (translated from Münster, 1830). Herewith he followed Cuvier (1824) who had figured nothosaur bones from the UM of the Lunéville area (Lorraine, France) and assigned them to large turtles and to *Plesiosaurus*, which was described in the same year (Conybeare, 1824). Meyer (1832: 309) compared the Bayreuth vertebrates with other still undetermined reptile remains from the Muschelkalk of France and Germany and assumed six different saurians among Münster’s specimens with the most common bones belonging to ‘*Plesiosaurus’*. Two skulls, the first of which was discovered in 1824, with large shiny black teeth were assigned to pycnodont fishes by Agassiz (1833–1844) and called *Placodus gigas* and *P. münsteri*. The reptile nature of these finds was discovered by Owen (1858). After 25 years of collecting in the Bayreuth Muschelkalk, Münster (1834) reported the here described articulated partial skeleton (UMO 1000) and other finds to the scientific community. Emphasizing a strange mix of plesiosaur and crocodile characters of the vertebrae and significant differences of the extremities, Münster (1834) concluded that the partial skeleton was a ‘completely new genus of wonderful shape that combines special characters of different animal genera and called it *Nothosaurus mirabilis*, (Bastard Saurus, made of different species of animals)’ (translated from Münster, 1834). In the same publication Münster (1834) also mentioned commonly found big bones and teeth that he called *Dracosaurus*. Relating the teeth of *Dracosaurus* with *Nothosaurus mirabilis* remained out of Münster’s consideration. He rather emphasized the similarity of *Conchiosaurus*, a fragmentary skull described by Meyer (1833), which later proved to be a senior synonym of *Nothosaurus* as is *Dracosaurus* (Rieppel & Wild, 1996). Despite the priority of *Conchiosaurus*, *Nothosaurus* Münster, 1834 was conserved by the International Commission for Zoological Nomenclature (for details see Rieppel & Brinkmann, 1996; Rieppel & Wild, 1996). Münster (1834) also named two other *Nothosaurus* species of which *Nothosaurus giganteus* is also still valid today (Rieppel & Wild, 1996). Bronn (1835–1838) reported the state of the art of fossils found in the vicinity of Bayreuth. In the second, enlarged catalogue of the fossils that Münster dedicated to the ‘Kreis-Naturalien-Sammlung zu Bayreuth’ to remain in Bayreuth ‘for all future times’, Braun (1840) illustrated the postcranial skeleton, which has been included in a mount (UMO 1000), and countless bones of the Bayreuth Muschelkalk vertebrates on 22 plates. The high amount of well-preserved remains of marine reptiles from the UM of the vicinity of Bayreuth culminated in a magnificently illustrated folio compendium published by Hermann von Meyer (1847–1855). In this monograph Meyer gave a first detailed description and illustration of UMO 1000 but only on the articulated vertebral column because of the uncertainty, which bones were added to the skeleton in the mount (see below).

After Münster’s death C.F.W. Braun, the director of the Kreis-Naturalien-Sammlung complained about fossil dealers who obtained several skulls from the quarry workers and sold them to natural history museums, *e.g*., London, Berlin, and Munich (Weiss, 1937, 1983). In the 1870s a second period of local vertebrate collecting commenced, when the Bayreuth carpenter Johann Strunz began to assemble a large collection within 30 years. He controlled the then active quarries and received in 1893 a complete cervical vertebral column from the Bindlacher Berg, which was described by Geissler (1895) as *Nothosaurus strunzi*. This find proved later to belong to *Pistosaurus longaevus* (Huene, 1949; Sues, 1987). It was sold in 1909 and 1912 by his son Christian Strunz together with almost his entire collection to the Senckenberg Museum, where he was engaged as a skillful preparator (Diener & Zapf, 2004). After 1950 a new generation of private collectors assembled vertebrate fossils from the UM of Upper Franconia farther to the West in the area of Kulmbach and Kronach, however, no more articulated material was found so far. Some of this material went to the State Museum of Natural History, Stuttgart.

In summary, the UM quarries in the vicinity of Bayreuth had so far produced a considerable number of isolated skulls and numerous (maybe thousands) isolated postcranial elements (*e.g*., Münster, 1834; Bronn, 1835–1838; Braun, 1840; Meyer, 1847–1855; Strunz in Huene, 1933; Rieppel, 2000). Contrary to this, only two articulated and partially complete skeletons are known: that of *Nothosaurus mirabilis* (holotype UMO 1000) and that of *Pistosaurus longaevus* (SMF 4041). A third partial skeleton from the Bindlacher Berg quarries was described and illustrated as outline sketch by Meyer (1847–1855: 48, pl. 34, fig. 4) and tentatively assigned to *Pistosaurus*; this skeleton cannot be located anymore (Rieppel, 2000).

The relative abundance of bones compared to other UM areas is here regarded as an artefact of the collecting activities initiated by Count Münster and pursued by his successors. They instructed quarry workers who formatted cobblestones by hand and developed keen eyes to identify bones, and were fairly well paid by the collectors. In this time of manually operated quarries, comparable collectors in most other vertebrate rich Muschelkalk regions were not present, maybe with a few short-term exceptions at Esperstädt, Lunéville, and Crailsheim.

#### The Bayreuth Muschelkalk quarries: their stratigraphical and paleogeographic position

The historical UM quarries in the vicinity of Bayreuth belong to a Muschelkalk ridge that stretches over ca. 80 km NW–SE in front of the Franconian Line in the Franconian Bruchschollenland (block faulted area). The ‘Franconian Line’ is a major fault that separates the metamorphic and granitic Paleozoic basement in the East from the Mesozoic in the foreland. During UM times the Bayreuth area was situated only some tens of kilometers West of the shoreline of the Bohemian Massif (Hagdorn & Rieppel, 1999).

**Figure 1:**
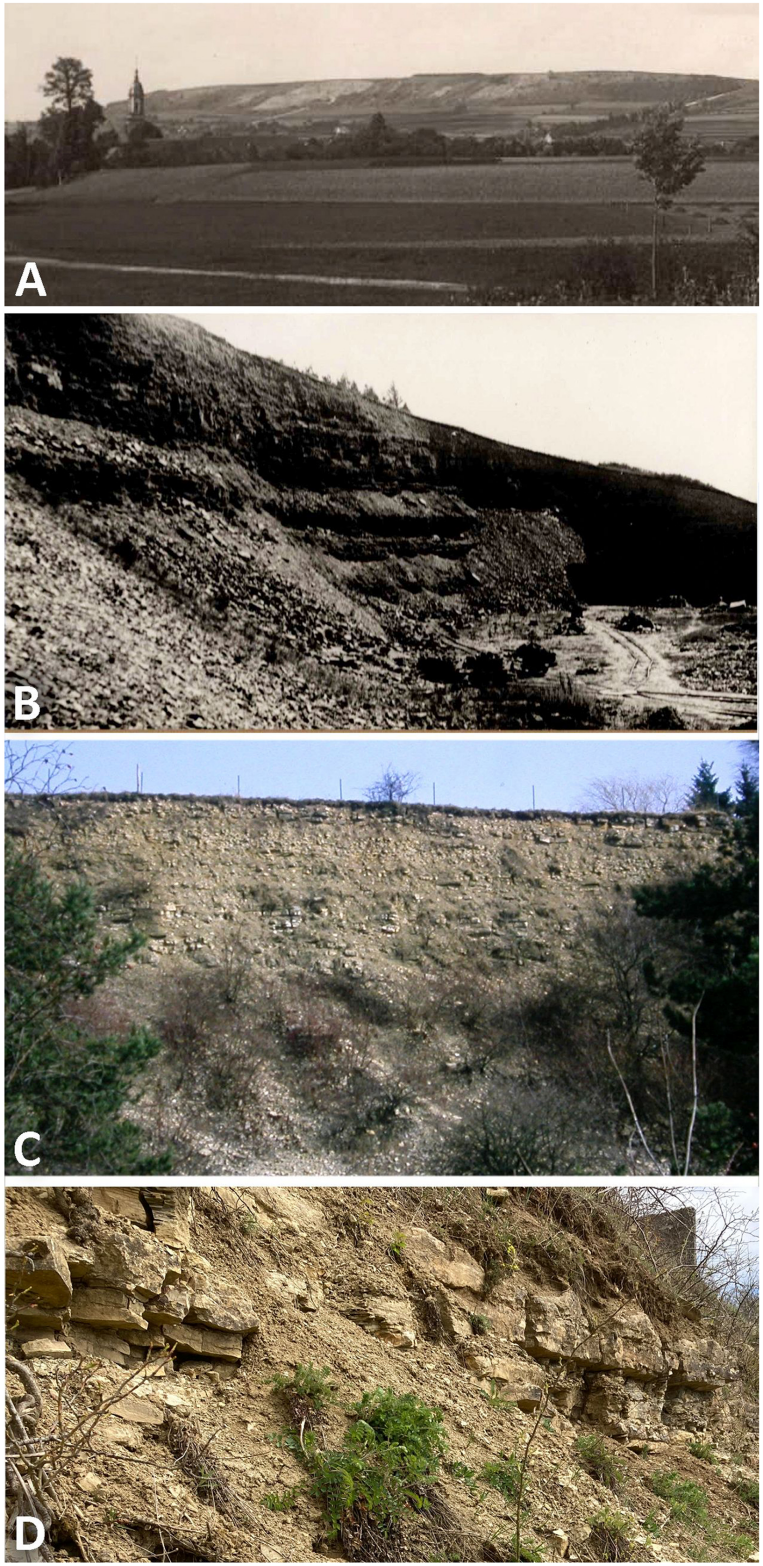
Photographs of quarries. The historical Upper Muschelkalk quarry Bindlach at the Bindlacher Berg East of Bayreuth. (A) The Bindlacher Berg seen from West with the Bindlach church in the foreground. The quarry (light greyish) stretches over several hundreds of meters along the ledges. Photo courtesy: UMO; (B) detail of the quarry when still active. Photo courtesy: UMO. (C) The quarry in February 1990 with the upper part of the section still open. Photo: H. Hagdorn. (D) The uppermost part of the quarry, above the vertebrate bearing strata, in April 2022. Photo: H. Hagdorn.

**Figure 2:**
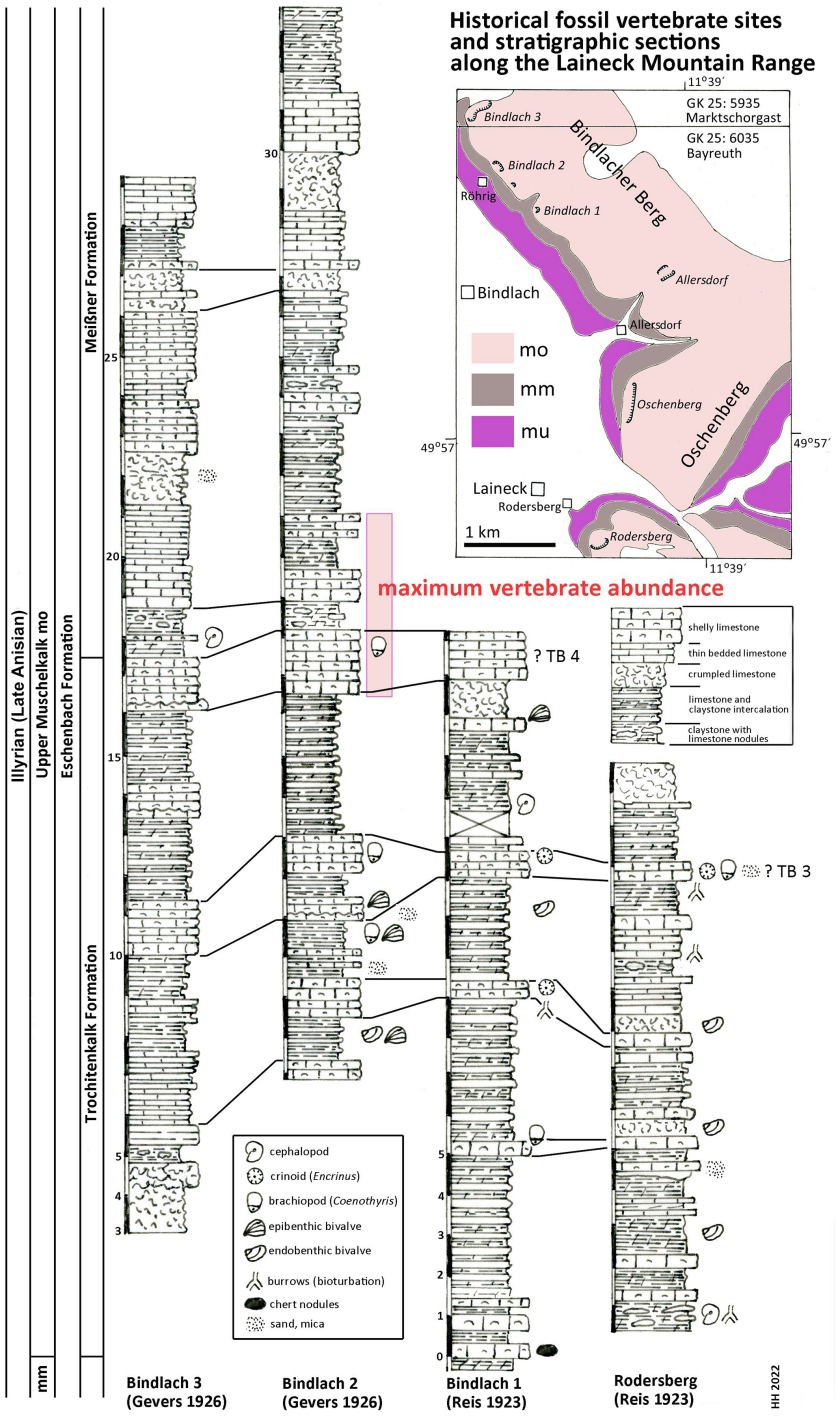
Stratigraphic sections. Historical fossil vertebrate sites and stratigraphic sections along the Laineck Mountain range East of Bayreuth. The sections drawn after historical descriptions by Reis (1923a, 1923b) and Gevers (1926) are correlated and interpreted according to recent lithostratigraphic subdivisions to the Trochitenkalk and Meißner formations of the basin facies (Hagdorn et al., 2020), alternatively to the marginal Eschenbach Formation (Geyer & Friedlein, 2020). Maximum vertebrate fossil abundance according to observations of quarry workers as reported by Gevers (1926). Abbreviations: GK 25, Geological Map of Bavaria 1:25.000 (Emmert, 1977; Emmert & Stettner, 1995); mu, Lower Muschelkalk; mm, Middle Muschelkalk; mo, Upper Muschelkalk; TB 3, Trochitenbank 3 resp. Hauptencrinitenbank; TB 4, Trochitenbank 4 resp. Terebrateldickbank.

The Muschelkalk quarries run at Münster’s times were located along the ledges of the Bindlacher Berg and the Oschenberg some 9 km East of Bayreuth (Figs. 1, 2). The oldest and largest quarry was situated 1.500 m Northeast of Laineck a village on the western slope of the Oschenberg (Geological Map of Bavaria 1:25.000, sheet 6035 Bayreuth: R 44 74200, H 55 37000), in the older literature often called Oschersberg or simply Lainecker Berg or Lainecker Höhenzug (Laineck Mountain Range). According to Emmert (1977), this quarry mentioned as early as 1787 exposed the lower part of the UM along the ledge over ca. 400 m. At the same time, two smaller quarries were situated 1.200 m Northeast of Bindlach (R 44 72900, H 55 39540) on both sides of the old road to Hof (Reis, 1923a, 1923b). Stratigraphical sections of these exposures documenting detailed positions of the vertebrate layers have not been measured. The large quarries at the western slope of Bindlacher Berg North and East of the hamlet Röhrig (R 44 72500, H 55 39850) and at the road to the village Benk (Geological Map of Bavaria 1:25.000, sheet 5935 Marktschorgast: R 44 72300, H 55 40700) were opened around 1900. Sections of these quarries have been measured by Gevers (1926). In the 20^th^ century, vertebrate remains were also discovered in additional quarries, *e.g*., at Rodersberg approx. 1.5 km South of the abandoned Oschenberg quarry (R 44 73750, H 55 35350), and 4 km West of Hegnabrunn (R 44 68 080, H 55 50 050). However, all the Muschelkalk vertebrates discovered at Münster’s times came from either the Oschenberg or the old Bindlacher Berg quarries. These outcrops were long ago refilled, overbuilt or more or less covered by vegetation and are now hardly to be identified (Fig. 1).

According to Gevers (1926: 290 and Tab. after p. 288) the highest abundance of *Nothosaurus* and *Placodus* remains in the big Bindlach quarry North of Röhrig was reported by the quarry workers in a ca. 4 m thick section in the middle part of the profile (Fig. 2). This section begins with 165 cm of thickly bedded shelly packstones with brachiopods (*Coenothyris vulgaris*), which is here interpreted as an equivalent of Trochitenbank 4, rather than as an equivalent of the Spiriferinabank as suggested by Gevers (1926). In the Bayreuth area, this marker bed is devoid of the marker brachiopod *Punctospirella fragilis* and is here assumed to be more than 10 m upsection. Upsection, 62 cm of nodular limestones, interbedded with marl are following, then again 80 cm shelly packstones, and 121 cm thinly bedded claystones interrupted by two 14 and 17 cm thick packstone beds. Facies and fauna of the underlying beds verify the lithostratigraphic correlation with the Hassmersheim Member (in Bavaria called: ‘Zeller Tonsteinhorizont’) of the Trochitenkalk Formation, which is generally poor in crinoid remains in the Bayreuth area. This correlation is also confirmed by the profiles of the quarry at the road to Benk (Gevers, 1926: 290 and Tab. after p. 288), and South of Rodersberg (Reis, 1923b), which reach downsection almost the Middle Muschelkalk (Fig. 2).

Hence, marine reptile and fish remains are most common upsection of the Trochitenkalk Formation, at the base of the Meißner Formation (formerly Ceratitenschichten). This is biostratigraphically corroborated by ceratite finds. According to Frosch (1923), ceratites were generally very rare in the Bindlacher Berg quarries, slightly more abundant in the Oschenberg quarries, which were still accessible at Frosch’s times, and rather common near Rodersberg. However, the Oschenberg and Rodersberg quarries yielded four specimens of *Paraceratites flexuosus*, the earliest ceratite, but *Ceratites pulcher* and *C. robustus* are the most common ceratites in the Bindlach quarries (Frosch, 1923). The upsection following *C. compressus* is rarely found. Correlations of UM sections in the Upper Franconian Muschelkalk range including ceratite finds were published by Weiss (1954) and Welzel (1963). The UM above the robustus biozone was exposed in the 1990s during construction work at the three-leg-interchange of motorways A9–A70 northwest of Bayreuth and measured and documented by means of bed-by-bed collected ceratites (Hagdorn in Bachmann, Beutler & Hagdorn, 1999). Despite the carbonate dominated facies and invertebrate fauna much resembling the Trochitenkalk and Meißner formations, Geyer & Friedlein (2020) assigned it to the mixed siliciclastic and carbonatic but still marine Eschenbach Formation (Member 5) and drew the boundary farther NW, that is farther towards the basin centre. The Bindlach and Hegnabrunn formations, which were introduced by Diedrich (2012), are here regarded as redundant synonyms because of the similarity of their lithologies with the existing and well-defined units.

According to Münster (1834) report, the skeleton UMO 1000 was covered with calcareous marl and partly embedded in hard limestone. This coincides with the valuable observations by Christian Strunz (in Huene, 1933) reporting that reptile bones are most commonly found in a ‘Backel’ (irregular concretion or limestone nodule), often together with brachiopod shells. The partial *Pistosaurus* skeleton, skulls and most isolated bones had been found inside such a nodule (Strunz in Huene (1933)). Likely, this was also the case with Münster’s *Nothosaurus* skeleton (UMO 1000). Such concretions are typical of the above-mentioned nodular limestone horizons within the 4 m thick section of the reptile bone maximum. Strunz (in Huene, 1933) gives also an overview of abundance of vertebrate and invertebrate fossils in the Frühhaber Quarry at Bindlacher Berg, which corroborates the lithostratigraphic correlation.

In summary, the historical profiles of Reis (1923a, 1923b) and Gevers (1926) allow to correlate Münster’s quarries lithostratigraphically with the upper Trochitenkalk and lower Meißner formations (Fig. 2) and biostratigraphically with the flexuosus through compressus ceratite biozones (Hagdorn, 2020). This corresponds exactly to the UM interval of Crailsheim (Baden-Württemberg) and Bad Sulza (Thuringia) that yields identical fish and reptile faunas. This fauna in this stratigraphic position of the UM has been called Bayreuth Fauna by Hagdorn & Rieppel (1999). The age of the Bayreuth Fauna of the UM is latest Illyrian (late Anisian).

#### Paleoenvironment of the Bayreuth Upper Muschelkalk

In the Bayreuth quarries terrestrial influx is indicated by some horizons with fine sand and mica (Gevers, 1926), which is strongly increasing towards the Southeast. As mentioned above, the Bayreuth area was situated only some tens of kilometers West of the shoreline of the Bohemian Massif. The vertebrate bearing UM horizons of Bayreuth were deposited during the transgressive branch of the sea level under fully marine conditions in a marginal position offshore of the nearby Bohemian Massif. Other than in the slightly younger black shale conservation Lagerstätten of the Besano Formation of the southern Alps (Lombardy, Italy, and Ticino, Switzerland), associated or articulated skeletons remained extraordinarily rare on the well oxygenated and bioturbated Muschelkalk seafloor at a moderate depth between wave base and storm wave base (Wild, 1972).

### The Bayreuth fauna

From the numerous reptile taxa from the vicinity of Bayreuth (*e.g*., Münster, 1834; Bronn, 1835–1838; Braun, 1840; Meyer, 1847–1855), only the placodonts *Placodus gigas* and *Cyamodus rostratus*, the nothosaurs *Nothosaurus mirabilis* and *Nothosaurus giganteus*, and the pistosaur *Pistosaurus longaevus* ‘survived’ the thorough revision of Sauropterygia conducted by Rieppel in the late 1990s, the results of which are summarized in Rieppel (2000). The protorosaur *Tanystropheus conspicuus* is also known from Bayreuth but see Spiekmann & Scheyer (2019) for the taxonomic status of *T. conspicuus*.

During the Middle Triassic, the Germanic Basin was influenced by transgressions and regressions providing certain paleoecological conditions (*i.e*., habitats) that also influenced diversity and occurrences of marine reptiles (Hagdorn & Rieppel, 1999). Based on this, Hagdorn & Rieppel (1999) established seven ‘faunas’ exclusively for the Germanic Basin across the late Olenekian to early Carnian (Upper Buntsandstein to Middle Keuper). The ‘Bayreuth fauna’ is representative for numerous lower UM (late Anisian) sites in mainly southern Germany but also from Alsace and Lothringen that provide a transgressive phase with nearly fully marine conditions (Hagdorn & Rieppel, 1999).

The Bayreuth fauna is clearly dominated by the genus *Nothosaurus*, which is represented by four taxa. *Nothosaurus mirabilis* is the dominant form, whereas *N. giganteus* and the two small nothosaurs are rare (Rieppel & Wild, 1996; Rieppel, 2000). *Nothosaurus mirabilis* is represented by several isolated skulls, dozens of isolated bones and the almost complete and partially articulated postcranial skeleton (UMO 1000), the holotype of the taxon (Rieppel & Wild, 1996; Rieppel, 2000). *N. marchicus* (formerly *N. venustus*, Rieppel & Wild, 1996) is only represented by a few isolated elements, and *N. giganteus*–known by some skull material as well as postcranial bones–is also rare in Bayreuth (Münster, 1834; Rieppel & Wild, 1996; Rieppel, 2000). *N. juvenilis* is only known from a single skull from a locality close to Heidelberg (Edinger, 1921; Rieppel, 2000). Due to size and morphological differences (see below), the nothosaurs followed different hunting and feeding strategies and thus occupied different niches and avoided so direct competition.

Given on the number of isolated teeth, the durophagous placodonts (*Placodus*, *Cyamodus*) are very common in the fauna (*e.g*., Strunz in Huene (1933)), loosing frequently teeth during feeding by normal tooth replacement. However, not only isolated teeth but also skulls and lower jaws of placodonts had been found. *Placodus gigas* was here clearly the dominant form, occurring in higher numbers than *Cyamodus*. The pistosaur *Pistosaurus longaevus* is known from Bayreuth by two skulls, one of which is lost, and one, maybe two (see above), partially preserved postcranial skeletons (Meyer, 1847–1855; Geissler, 1895; Sues, 1987; Rieppel, 2000). As usual in the Germanic Basin, ichthyosaurs are extremely rare, only documented by a few vertebrae (N. Klein and S. Eggmaier, personal observations at UMO collection in 2022). The archosauromorph *Tanystropheus conspicuus* is a constant but also rare faunal element in Bayreuth. Pachypleurosaurs are generally rare in the Bayreuth Fauna (Hagdorn & Rieppel, 1999). Mainly the occurrence of *Pistosaurus*, which is always interpreted as an open marine form and the rarity of pachypleurosaurs that are lagoonary or near shore inhabitants, support the increase of sea level and the beginning of a transgressive phase documented in the sediments of the lower UM during the late Anisian. However, nothosaurs were obviously less affected by rising or sinking sea level, since they occur throughout the entire Muschelkalk and elsewhere (see below). The occurrence or absence of placodonts, mainly that of *Placodus* is more difficult to assess and might depend on the availability and surviving of their feeding grounds (*i.e*., mussel banks) during sea level changes.

The composition of the Bayreuth Fauna differs from the—somewhat younger—early Ladinian (also UM) Hohenlohe/Lunéville Fauna (Hagdorn & Rieppel, 1999), which lacks *Placodus* and *Pistosaurus* but contains additionally *Simosaurus*, *Blezingeria*, a large pachypleurosaur, and *Cyamodus kuhnschnyderi*; *Nothosaurus* is still represented by *N. mirabilis, N. giganteus*, and the small *N. jagisteus*.

Comparing the Bayreuth Fauna with other Middle Triassic faunas outside the Germanic Basin is hampered by the unique environmental conditions prevailing in the semi-enclosed shallow epicontinental sea dominated by transgressive and regressive phases over long periods, and by exact stratigraphic correlation. The South Alpine Besano Formation of comparable late Anisian through early Ladinian age consisting of alternating laminated dolomitic beds and bituminous shales was deposited in a shallow marine setting at 30–130 m water depth (Furrer, 1995). The lowermost portion of the middle part of the Besano Formation coincides with the establishment of an intraplatform basin (Röhl et al., 2001). The fauna thus consists of mainly vertebrates that preferred open marine habitats such as ichthyosaurs and thalattosaurs. However, placodonts and nothosaurs are also common as well as *Tanystropheus*. The Franconian Muschelkalk and the Besano Formation faunas were compared in more detail by Wild (1972) and Rieppel & Hagdorn (1997).

In the last two decades numerous Triassic marine reptile taxa were described and different faunas were established from Southwest China (summarized *e.g*., in Sun et al., 2016). Among these, stratigraphically closest to the Bayreuth Fauna is the middle Anisian Panxian Fauna of the Guanling Formation, which contains three marine reptile beds (Jiang et al., 2009). The lower bed indicates a rising sea level and contains *Placodus*, the nothosaur *Lariosaurus*, and three ichthyosaur taxa. The middle bed indicates deep water and contains *Nothosaurus*, the marine archosaur *Qianosuchus*, and an ichthyosaur. The upper bed indicates shallowing water with the eosauropterygian *Wumengosaurus*, the pachypleurosaur *Keichousaurus*, an ichthyosaur and a protorosaur.

## Aim

After the re-assemblage of the holotype (UMO 1000) of *Nothosaurus mirabilis* in 2009, a thorough morphological description of the entire skeleton is now possible for the first time after more than 180 years after its discovery.

## Materials and Methods

### Material

We re-studied the holotype of *Nothosaurus mirabilis* (Figs. 3–22), a fairly complete and partially articulated postcranial skeleton, which is housed in the Urwelt-Museum Oberfranken (UMO), Bayreuth, Bavaria, Germany under the repository number UMO 1000. The specimen is a historical find discovered in 1834. It has not been available for study for a long time. Recently, it underwent further preparation and was remounted.

**Figure 3:**
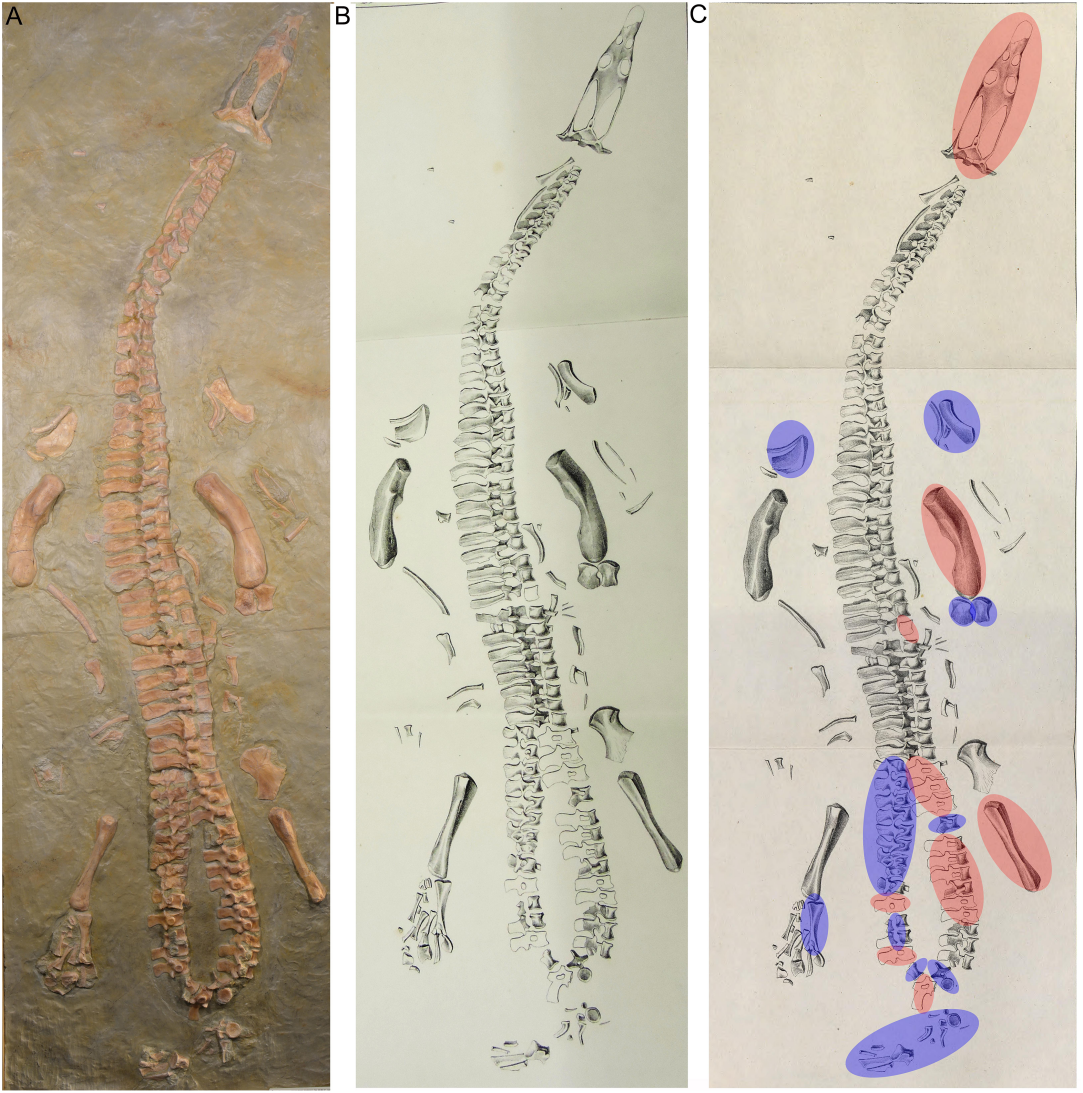
Historical mount. (A) Photo of the cast of the historical composite as exhibited in the Urwelt-Museum, Oberfranken in 2022; (B) sketch of the composite modified from Braun (1840); (C) same sketch: Bones marked in red do not belong to the original skeleton. They are added from other individuals or carved in gypsum; Bones marked in blue are elements belonging to UMO 1000 but are included in an anatomical incorrect position.

**Figure 4:**
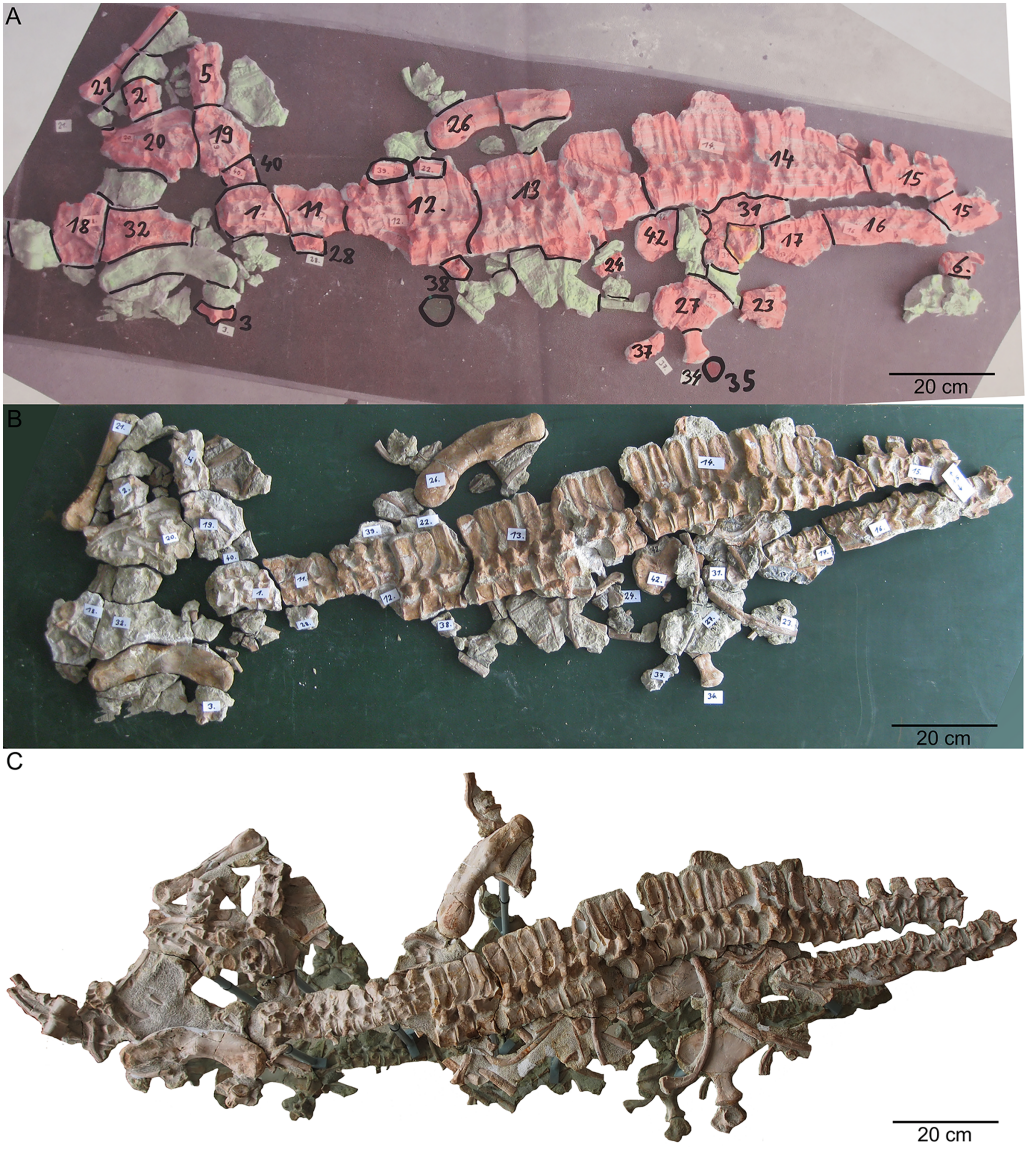
Remounting of UMO 1000. (A) Photo of the postcranium (UMO 1000) being reworked. In red are original bones that were included in the former composite. In green are in the collection recovered elements that have a clear fit. Those elements were added to the new mount of UMO 1000 in 2009; (B) reassembled postcranium; (C) UMO 1000 in 2022 after being reworked and mounted on a metal frame above a mirror.

**Figure 5:**
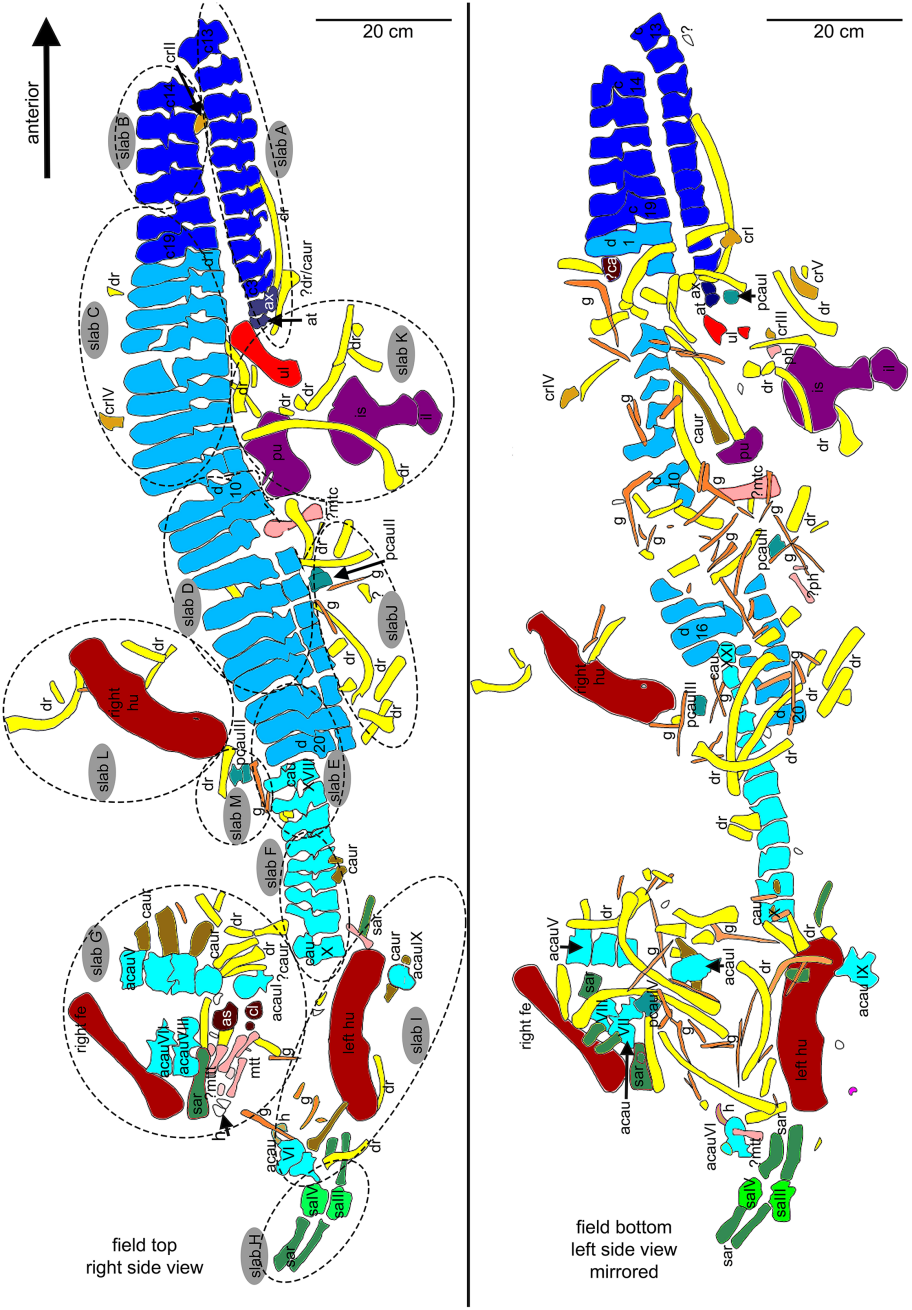
Interpretative overview sketches. (A) Overview sketch in field top view. Most elements are exposed from their right side. The dotted lines roughly indicate the 13 single slabs (A–M) in which UMO 1000 is broken. Note the well-articulated anterior vertebral column in contrast to the highly disarticulated posterior part of the vertebral column and the disarticulated and shifted limb and girdle elements; (B) overview sketch in field bottom view. Many elements are accessable now from both sides. Arabic numbers refer to the position of the articulated vertebrae of the neck and anterior trunk region. Roman numbers refer to isolated vertebrae and ribs without any reference to anatomical position.

**Figure 6:**
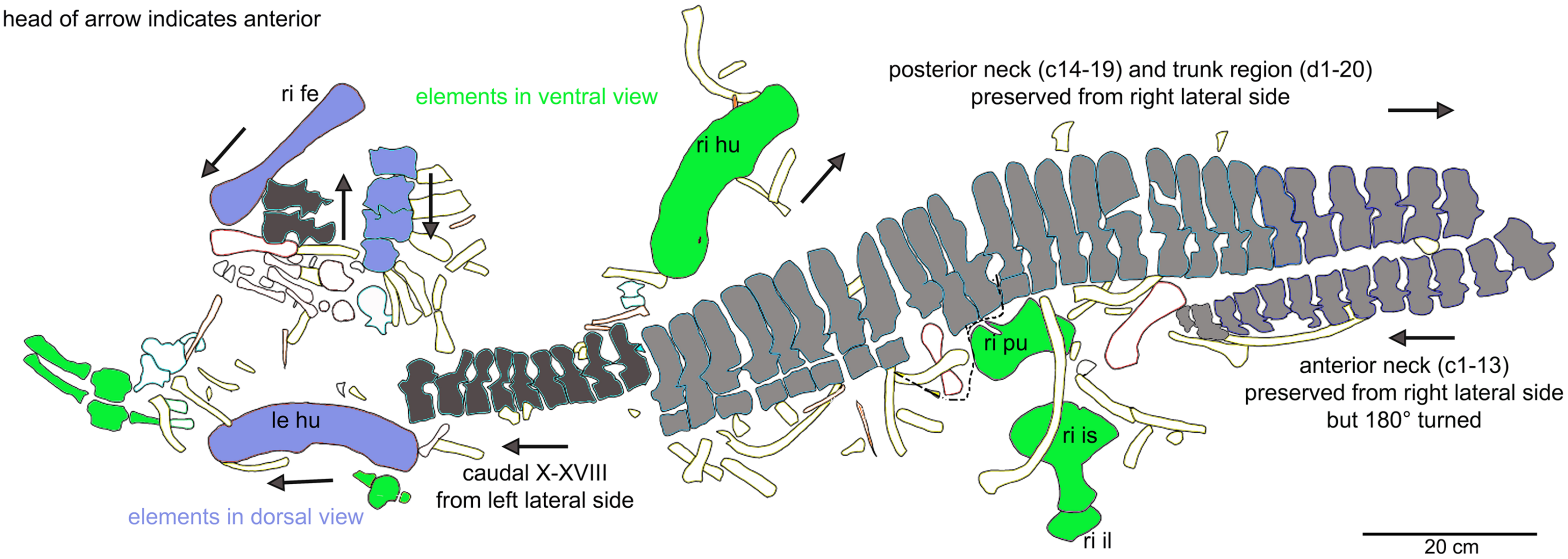
Orientation of bones. Interpretative outline sketch in field top view, different colors highlight the different bone sides in which the single elements of the individual are preserved: left (dark grey) or right lateral (bright grey) side and dorsal (purple) or ventral (green) view. Head of arrows always point in anterior direction of the respective element(s).

**Figure 7:**
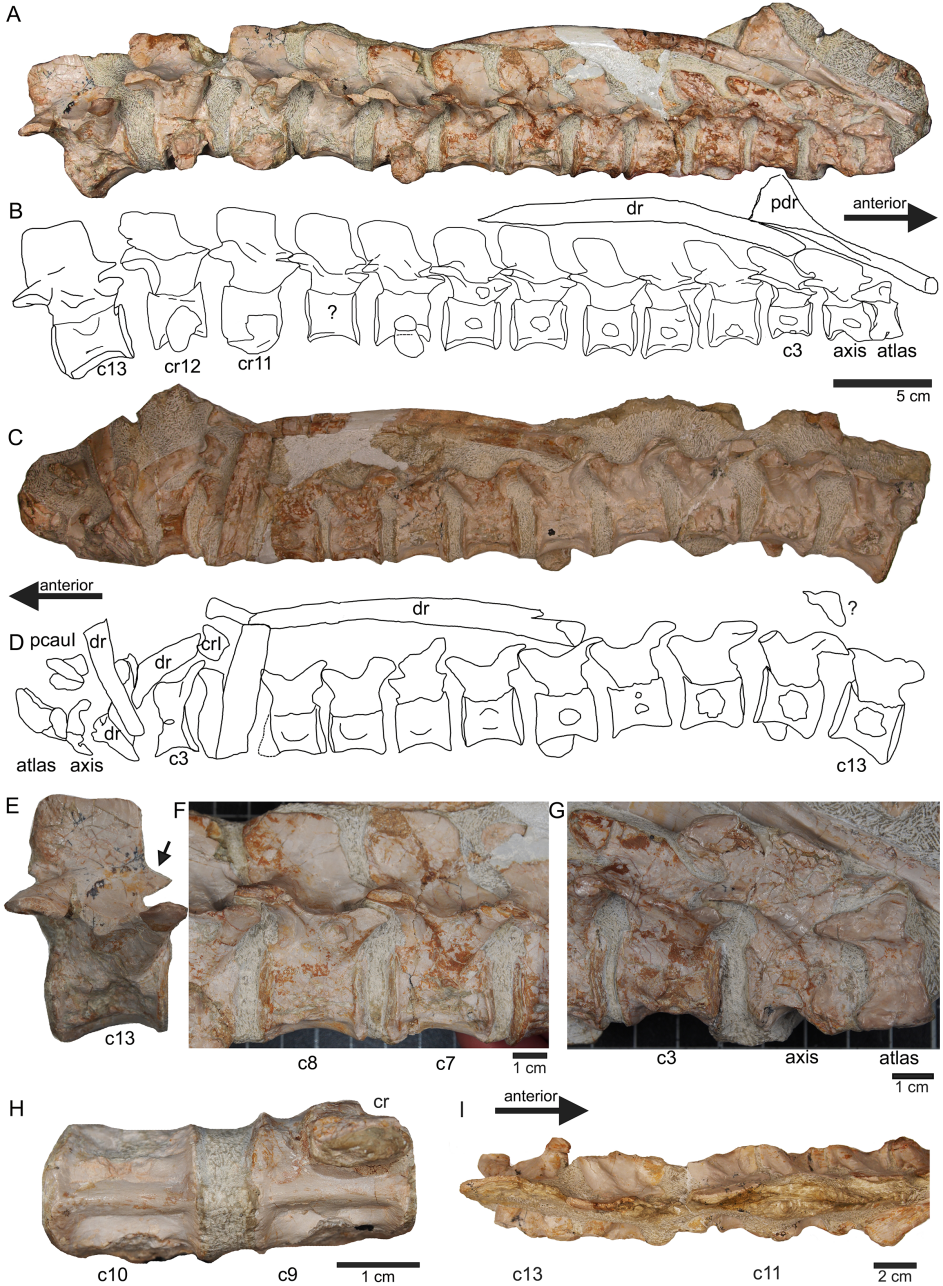
Anterior neck. (A) Slab A with the articulated cervical 1 (atlas), cervical 2 (axis) to cervical 13 in field top view, exposing the right lateral side of the vertebrae. Cervicals 9, 11, and 12 are still associated or articulated to their corresponding cervical ribs. In addition to cervical 1 to 13, a median element of a dorsal rib is visible and a complete short ?posterior cervical rib or anterior dorsal rib; Please note that in original find position this slab has turned nearly 180° with the atlas pointing caudally and the neural arches pointing ventrally (see Figs. 4–6); (B) outline sketch of slab A in field top view; (C) slab A in field bottom view, exposing the left lateral side of the vertebrae. The neural spines of the cervicals are not visible in this view and the anterior cervicals are obscured by fragments of dorsal ribs. Above atlas and axis is a posterior caudal vertebra (pcaudI) visible; (D) outline sketch of slab A in field bottom view; (E) right lateral side of cervical 13. Note the well-developed triangular zyogsophene (arrow) and the horizontally oriented zygapophyses; (F) right lateral side of cervical 7 and 8; (G) atlas, axis and cervical 3 in right lateral view. Note the change in morphology of the neural arch and the increase in size of the entire element; (H) cervical 9 (with the right cervical rib attached) and 10 in ventral view. Note the constricted and keeled centrum; (I) cervical 13 to 10 in dorsal view.

**Figure 8:**
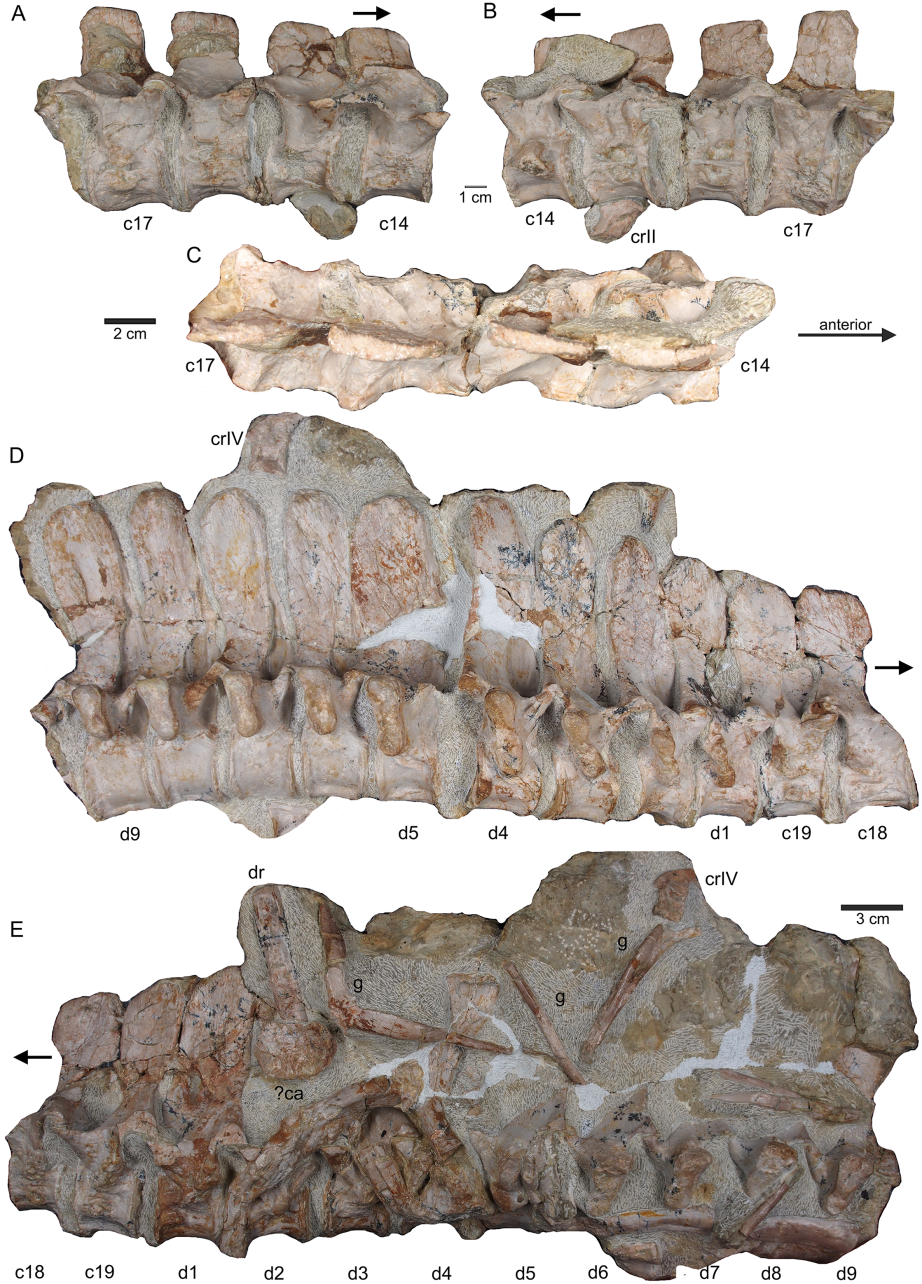
Posterior neck and anterior trunk. (A) Articulated cervical vertebrae 14 to 17 (slab B) in field top view, displaying the right lateral side; (B) slab B in field bottom view displaying the left lateral side of vertebrae. In left view, an isolated/disarticulated cervical rib (crII) is visible below cervical 15; (C) cervical vertebrae 14 to 17 in dorsal view; (D) string of articulated cervical 18 and 19 and dorsal 1–9 (slab C) in field top view exposing the right lateral side. Additionally, in this view is a cervical rib (crIV) visible; (E) slab C in field bottom view, exposing the left lateral side of vertebrae. Vertebrae d2 to d9 are incomplete in left view. Cervical rib IV is also visible from this side. In addition, a ?carpal element and fragments of dorsal ribs and gastralia are visible in this view. Please note that slab C is slightly bulged between dorsal 5 and dorsal 4 in field top direction. Arrows indicate anterior direction.

**Figure 9:**
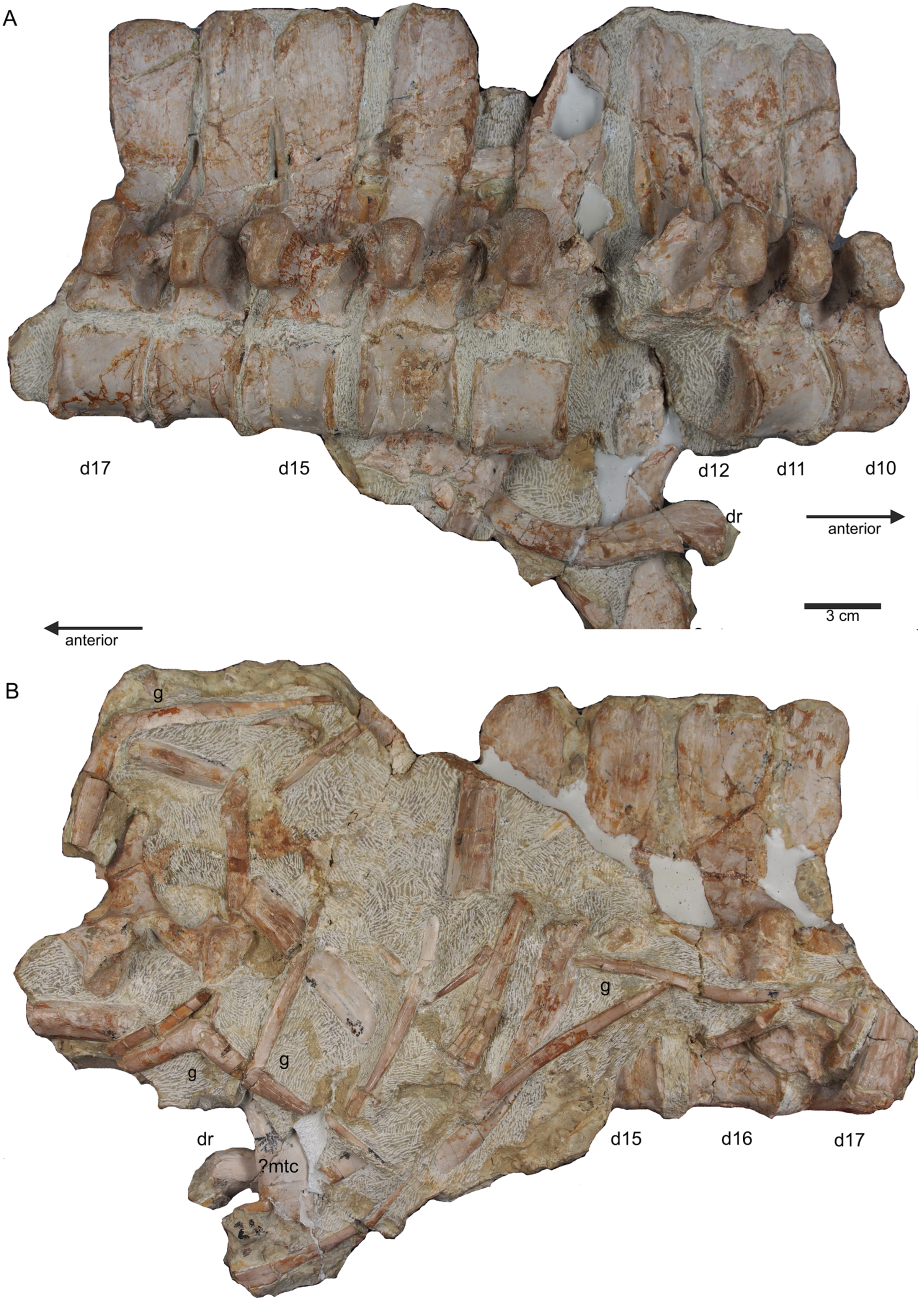
Middle trunk region. (A) Articulated dorsal vertebrae 10 to 17 (slab D) in field top view, exposing the right lateral side of vertebrae, a proximal part of a dorsal rib and a ?metacarpal element; (B) slab D in field bottom view, partially showing the left side of dorsal 15 to 17 and many fragments of dorsal ribs and gastralia; please note that slab D is slightly bulged between dorsal 12 and dorsal 13 in field top direction.

**Figure 10:**
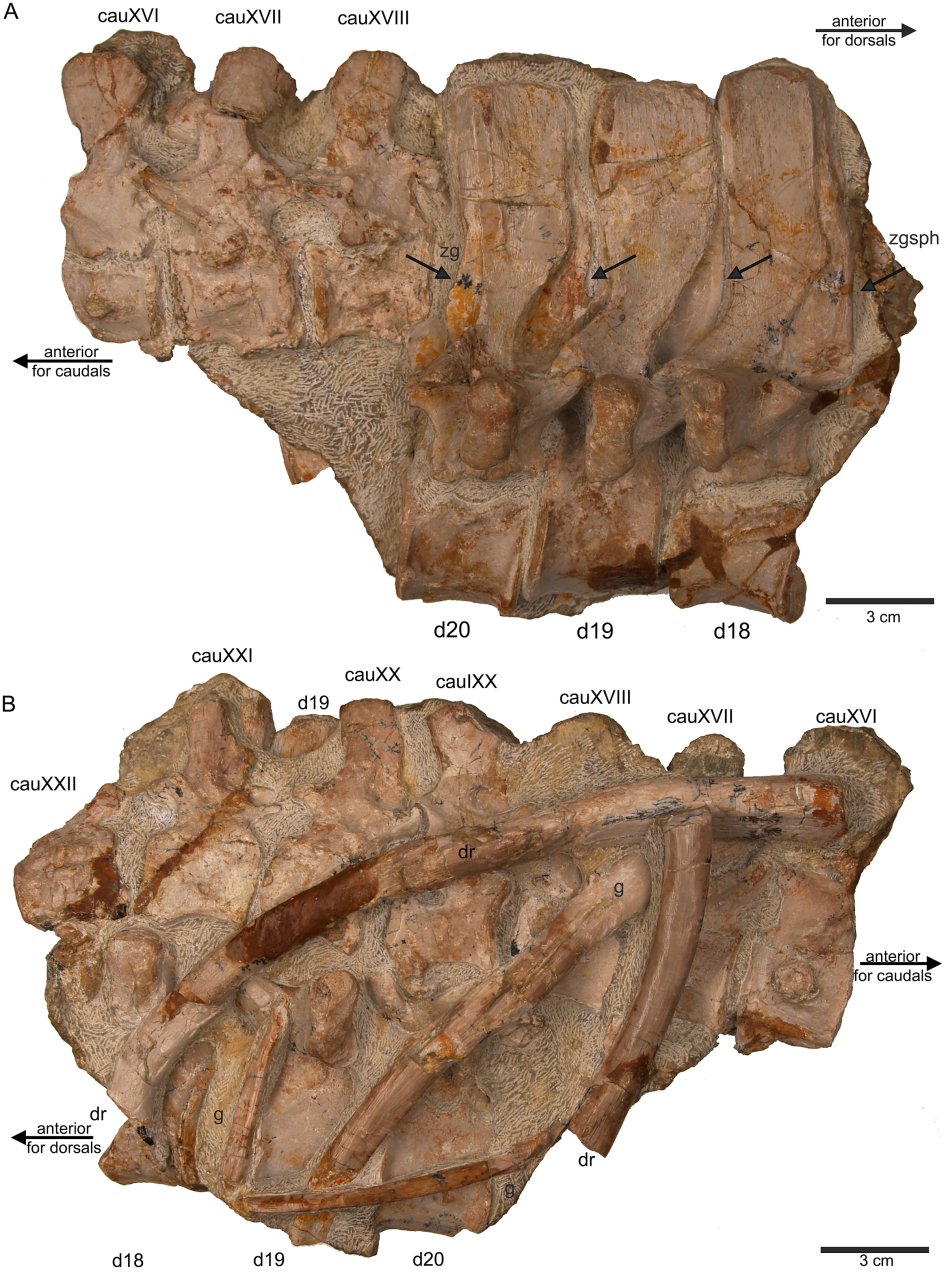
Last dorsals and posterior caudals. (A) Articulated dorsal vertebrae 18 to 20 (slab E) in field top view, showing their right lateral side. In addition, in this view three articulated caudal vertebra are visible in left view. The dorsals nicely show the supersized flat zygosphenes and zygantra (arrows); (B) slab E in field bottom view exposing the right side of the articulated string of caudals XVI to XXII. Caudals IXX to XXII are not visible in field top view because they are here covered by dorsal 18 to 20. In addition, the field bottom view display fragments of three dorsal ribs and of some gastral elements.

**Figure 11:**
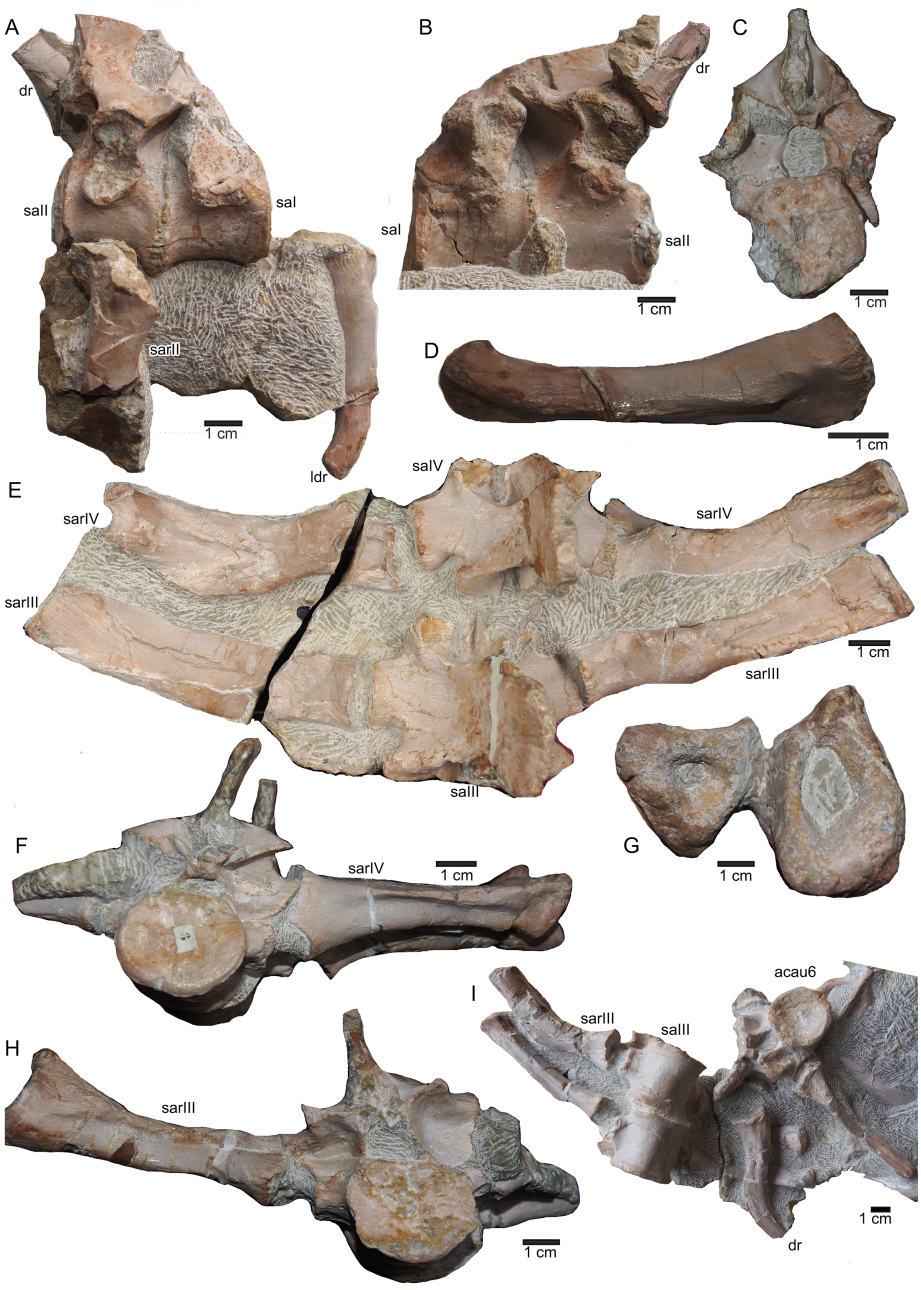
Sacral vertebrae. (A) Articulated sacral vertebra I and II in right lateral view with the last right dorsal rib associated. Sacral II is associated with the proximal part of the corresponding sacral rib. Please note that this slab has no connection to the other slabs. However, due to sediment, size and morphology we interpret this slab as belonging to the skeleton; (B) sacral vertebra I and II in left lateral view; (C) sacral vertebra II in posterior view; (D) last dorsal rib in antero-lateral view; (E) articulated sacral vertebrae III and IV (slab H) in dorsal view (as preserved in field bottom view). This slab has a connection to the rest of the skeleton *via* their right sacral ribs to slab I (see also Figs. 4, 5). Sacral III and IV are exposed from their ventral side in field top view; (F) Distal end of the left sacral ribs in lateral view; (G) sacral vertebra IV with its right sacral rib attached in posterior view; (H) sacral vertebra III with its right sacral rib attached in anterior view. (I) Sacral vertebrae III and IV in ventral view, depicting how they articulate to the main block close to anterior caudal vertebra 6 (see Fig. 5).

**Figure 12:**
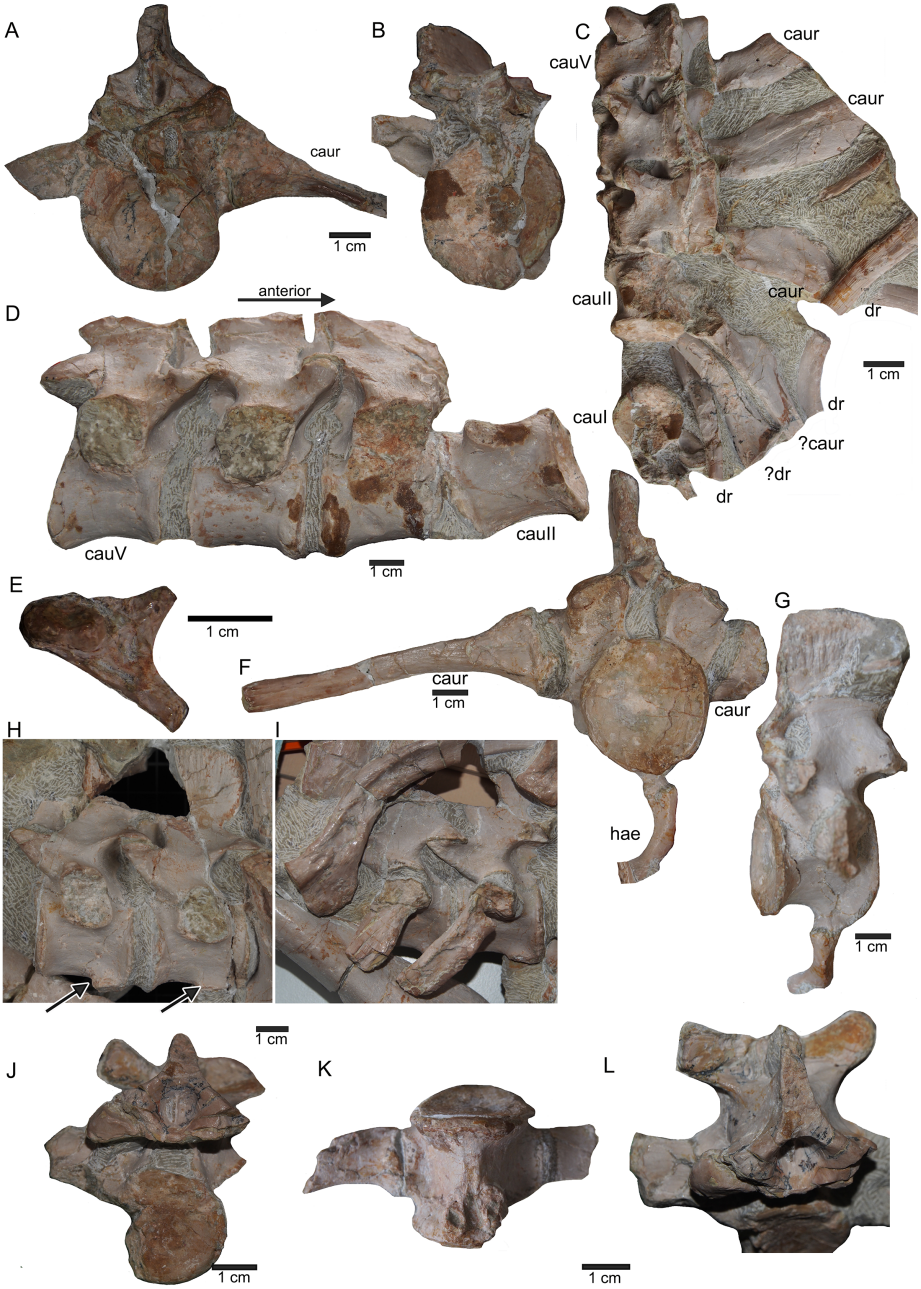
Anterior caudal vertebrae. (A) Isolated caudal I in anterior (field bottom) view, with its left caudal rib articulated and in (B) posterior view (field top). Caudal I belongs to slab G; (C) Dorsal view of articulated caudal I to V (slab G) associated with flattened caudal ribs from the left body side; (D) caudals II to V in field top view, displaying their right lateral side (slab G). The neural arch of caudal II is lost. Caudal I is disarticulated from the rest and turned 90°; (E) Isolated complete haemapophyses (visible in field top view on slab G); (F) caudal VI (slab I) in field top view, exposing its dorso-anterior face. Please note the articulated left and incomplete right haemapophyses; (G) caudal VI (slab I) in left lateral view; (H) Articulated caudal VII and VIII from left lateral side (field top view) (slab G). Note the articulation facet for the haemapophyses (arrows); (I) caudal VII and VIII from right lateral side (field bottom view). Note the attached caudal ribs and a proximal dorsal rib laying above caudal VII; (J) isolated caudal IX (slab I) in posterior view (field bottom view); (K) caudal IX in ventral view. Note the constricted and keeled centrum and the articulation facets for the haemapophyses; (L) Caudal IX in posterior-dorsal view.

**Figure 13:**
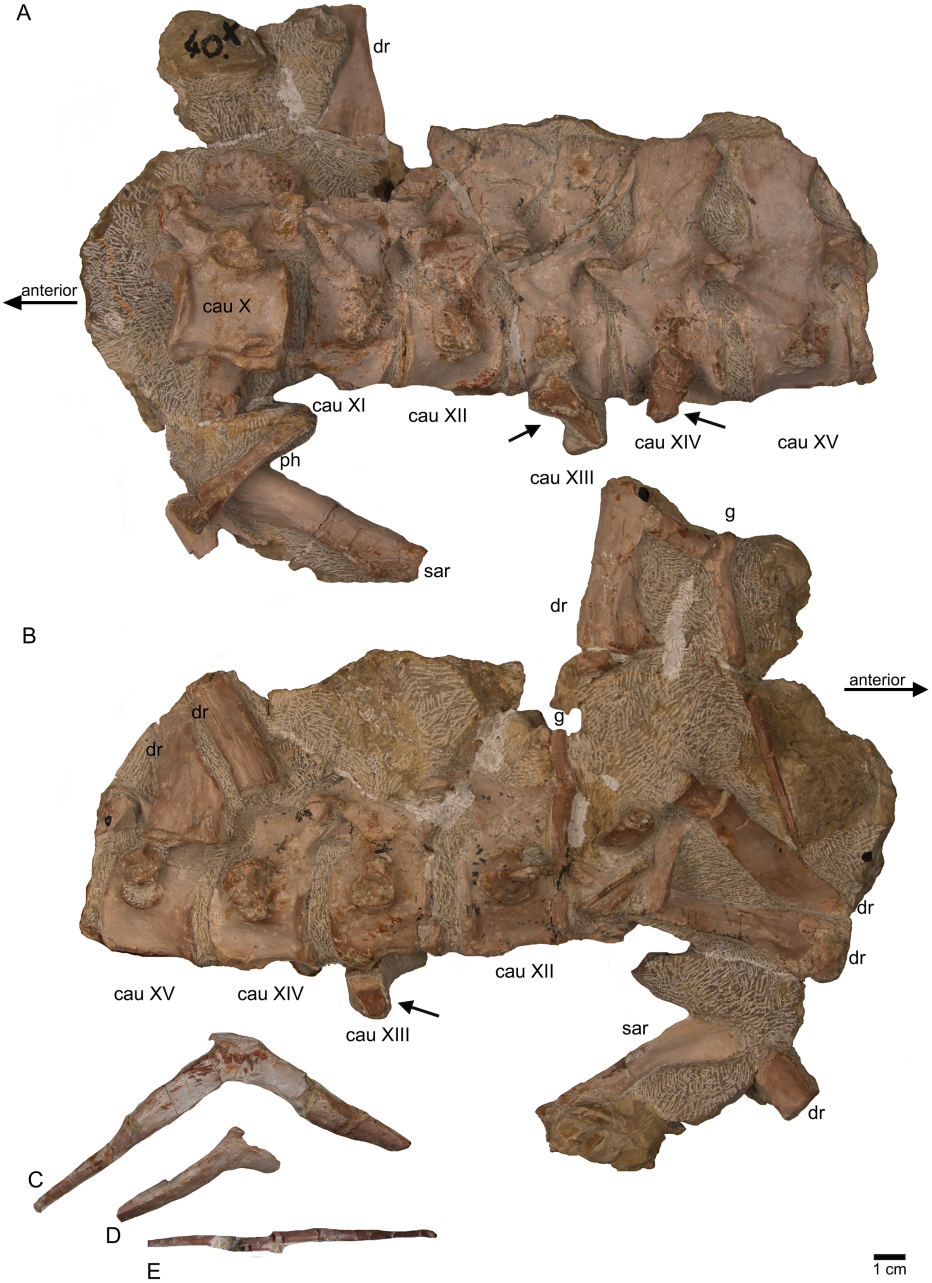
Caudal vertebrae X–XV. (A) Posterior caudal vertebrae X to XV in field top view (slab F), exposing their left lateral side. In addition, a slender phalange and a sacral rib are visible; (B) slab F in field bottom view, exposing the right lateral side of caudals X to XV; note the caudal ribs (arrows) associated with caudal XIII and XIV. The dorsal rib close to the gastral fragment continues on slab G. Please note that caudals XVI to XXII (slab E) are figured in Fig. 10; (C) median gastral element visible in field bottom view on slab C; (D) incomplete median gastral element visible in field bottom view in slab G; (E) Incomplete lateral gastral element visible in field bottom view on slab G.

**Figure 14:**
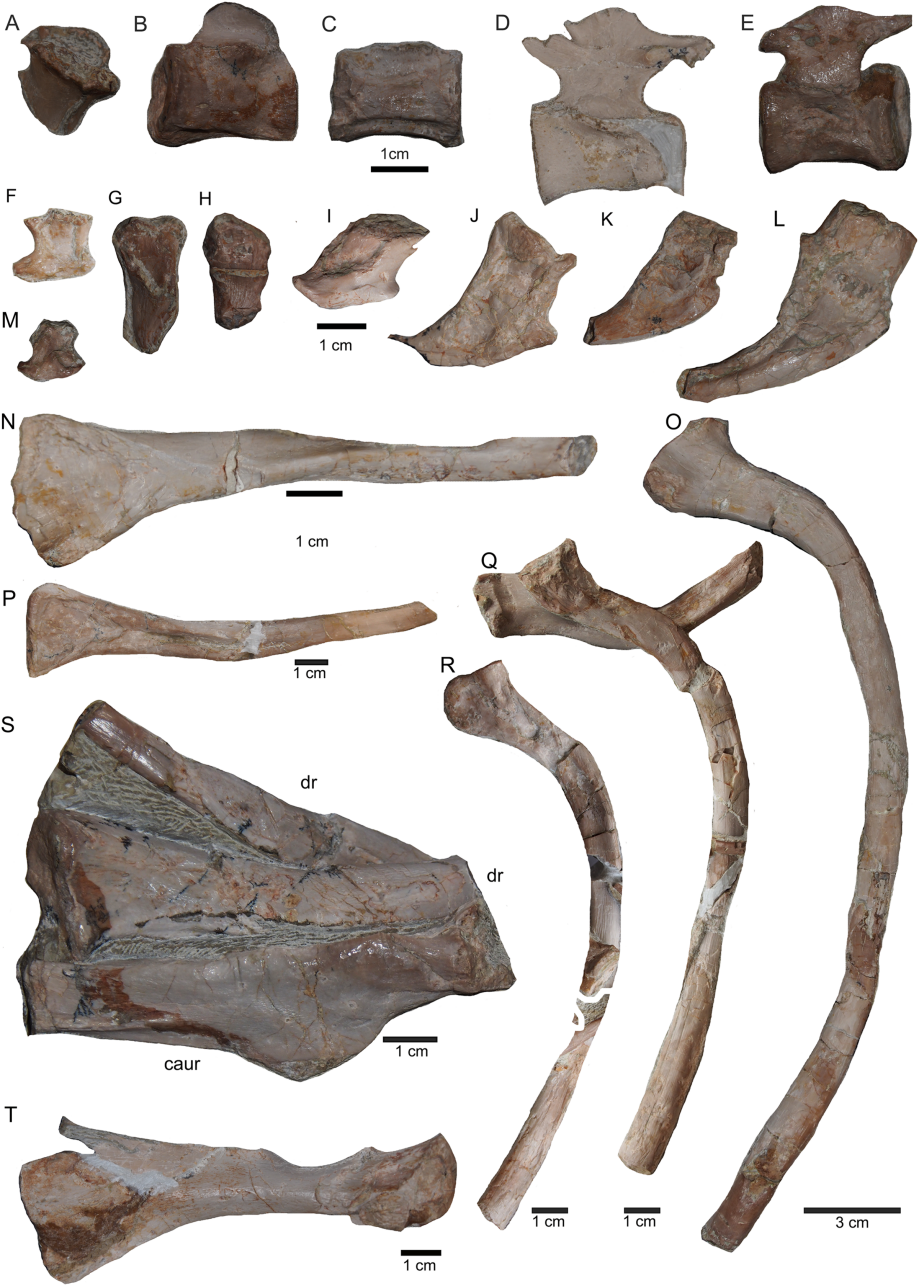
Posterior caudal vertebrae and various ribs. (A–E) Scattered and isolated posterior caudal vertebrae, all at the same scale. (A) Posterior caudal I (slab A) only visible in field bottom view; (B) posterior caudal II (slab J) mainly visible in field top view; (C) posterior caudal III (slab M). In field top view it is nearly complete and in (D) field bottom view, only the centrum visible from ventrally; (E) posterior caudal IV (slab G), only visible in field bottom view; None of these posterior caudal vertebrae has a facet for a haemapophysis. (F–M) Isolated cervical ribs all at the same scale. Except for G and H, all cervical ribs are figured in lateral view with the elongated process pointing posteriorly view; (F) anterior cervical rib I (slab A) only visible in field bottom view; (G) right cervical rib 12, articulated to cervical vertebra 12 (slab A); (H) Right cervical rib 11, articulated to cervical vertebra 11 (slab A); (I) Cervical rib IV (slab C) in field top and (J) field bottom view (mirrored); (K) cervical rib V (slab K) only visible in field bottom view; (L) anterior cervical rib III (slab K) only visible in field bottom view; (M) cervical rib II (slab B) only visible in field top (right) view; (N) short rib visible in field top view on slab A. It is unclear if this represents a posterior cervical or an anterior dorsal rib (compare to Fig. 9E); (O) Large (>26 cm) distally incomplete dorsal rib, visible in field top view, laying on the pubis and ischium (slab K); (P) posterior dorsal or anterior caudal rib visible in field bottom view, laying below the pubis (slab K) (mirrored); (Q) distally incomplete dorsal rib (>24 cm) associated with the proximal part of another dorsal rib (slab G) visible in field bottom view; (R) distally incomplete dorsal rib (>21 cm) (slab G) visible in field bottom view; (S) three ribs laying between caudal vertebrae I and II on slab G visible in field top view. The rib in front is a caudal rib whereas the other two ribs are dorsal ribs. Note the ventrally pointing crest of the caudal rib in front (which is the posterior one when in anatomical correct position). The same ribs are also figured in Fig. 12C but from a different angle; (T) ?Sacral or caudal rib visible in field top view on slab G.

**Figure 15:**
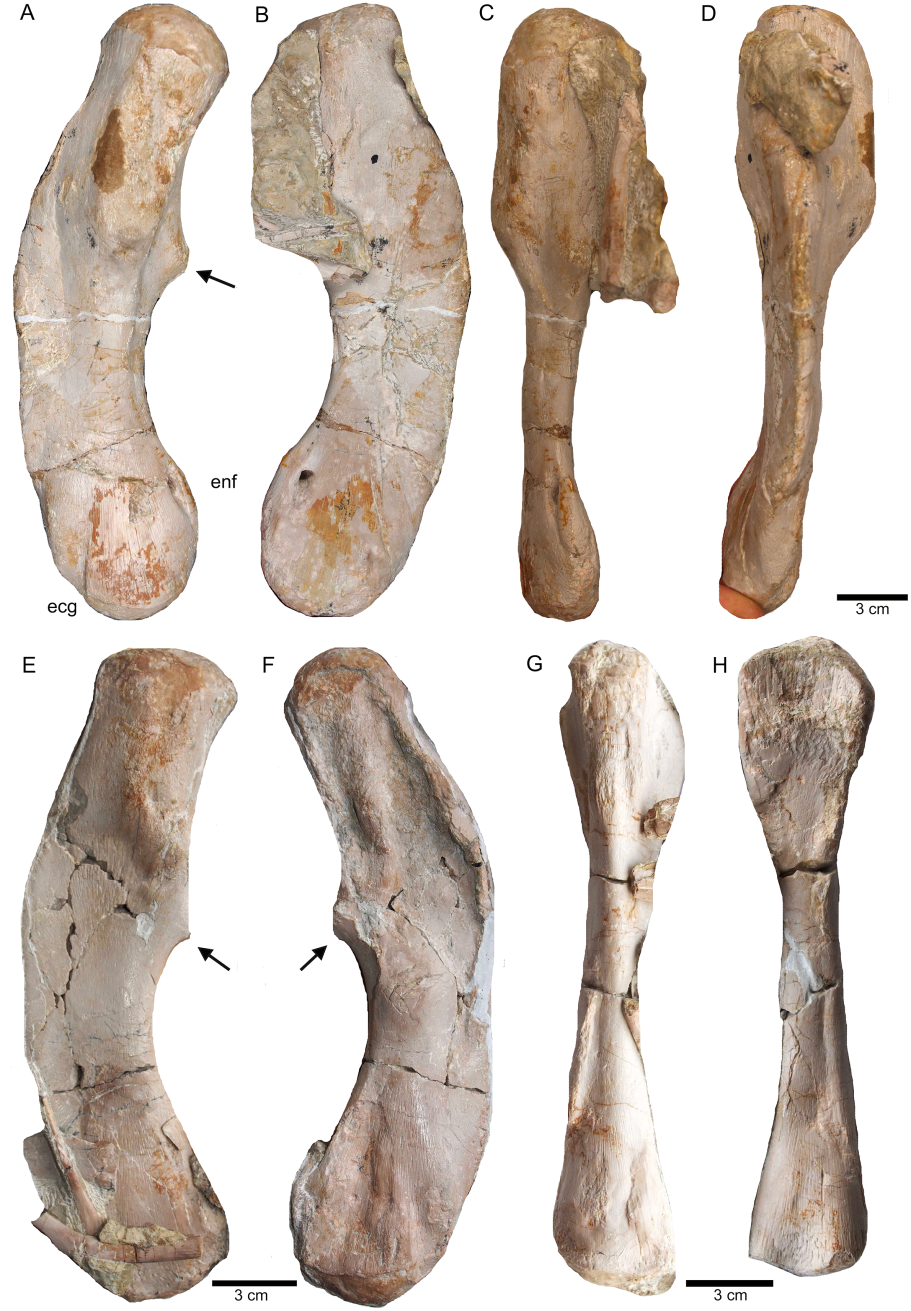
Humeri and femur. (A) Right humerus in ventral view (field top on slab L); (B) right humerus in dorsal view (field bottom slab L); (C) Right humerus in postaxial (medial) view; (D) right humerus in preaxial (lateral) view; (E) left humerus in dorsal view (field bottom view on slab I); (F) left humerus in ventral view (field top view on slab I); (G) right femur in dorsal view (field bottom view on slab G); (H) right femur in ventral view (field top view on slab G). The arrow marks a very prominent edge, which is unique in this humerus morphotype.

**Figure 16:**
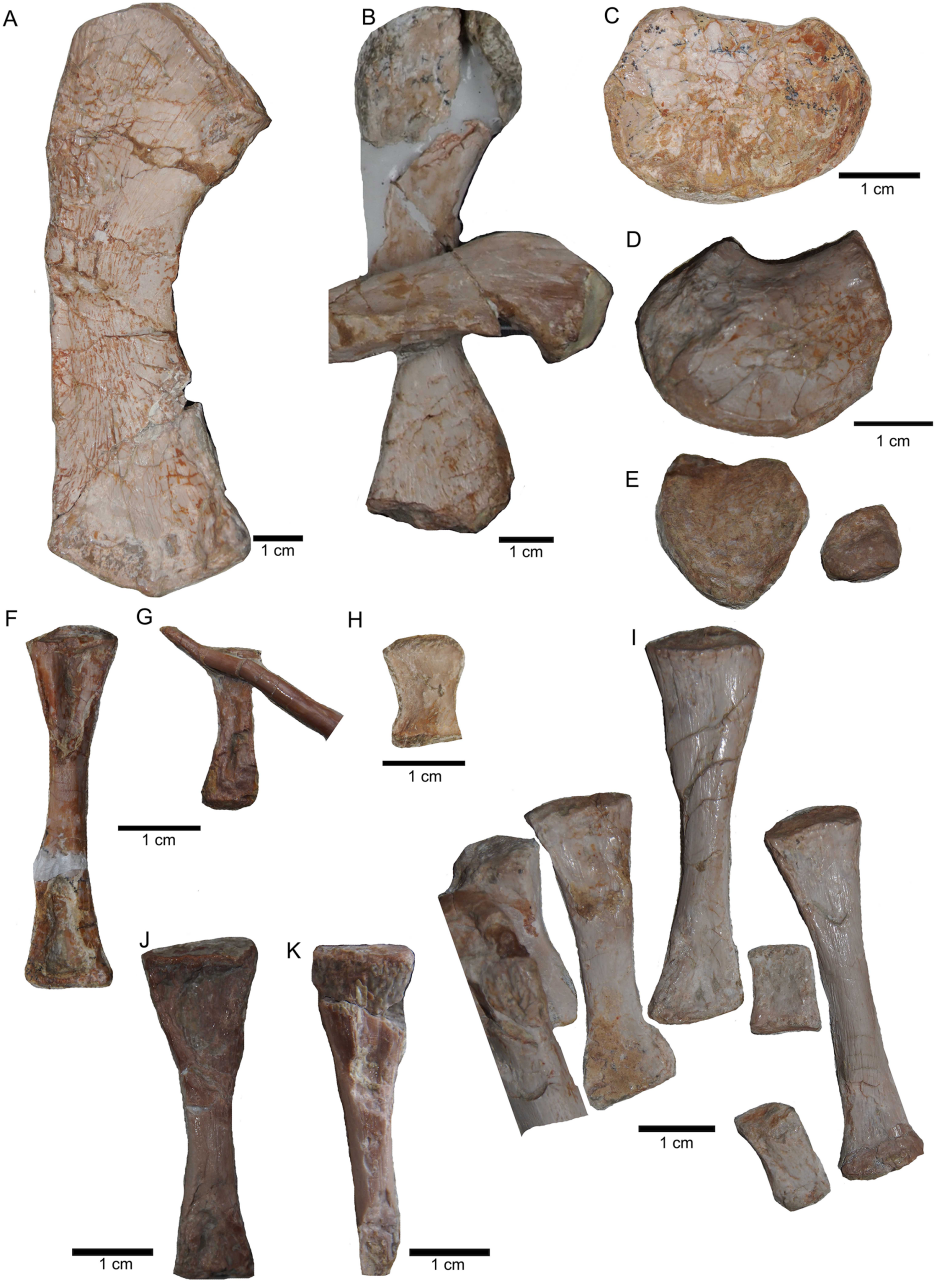
Zeugopodial and autopodial elements. (A) Ulna visible on slab K in field top view (mirrored); (B) ?Radius overlain by a proximal dorsal rib fragment visible on slab D in field top view; (C) Possible intermedium or ulnare visible on slab C in field bottom view; (D) Astragalus, visible in field top view on slab G; (E) Calcaneus, and ?centrale visible in field top view on slab G; (F) ?Metatarsale visible in field bottom view on slab J; (G) Phalange is associated with the ?metatarsale figured in Fig. 16F. The phalange is overlain by a lateral gastral rib fragment, which is here only incompletely figured; visible in field bottom view on slab J; (H) Phalange visible on slab K in field bottom view (close to the cervical rib III); (I) Associated metatarsals and phalanges visible in field top view on slab G; (J) Isolated ?metatarsale visible in field top bottom on slab I; (K) Incomplete ?metatarsale visible in field top view on slab F.

**Figure 17:**
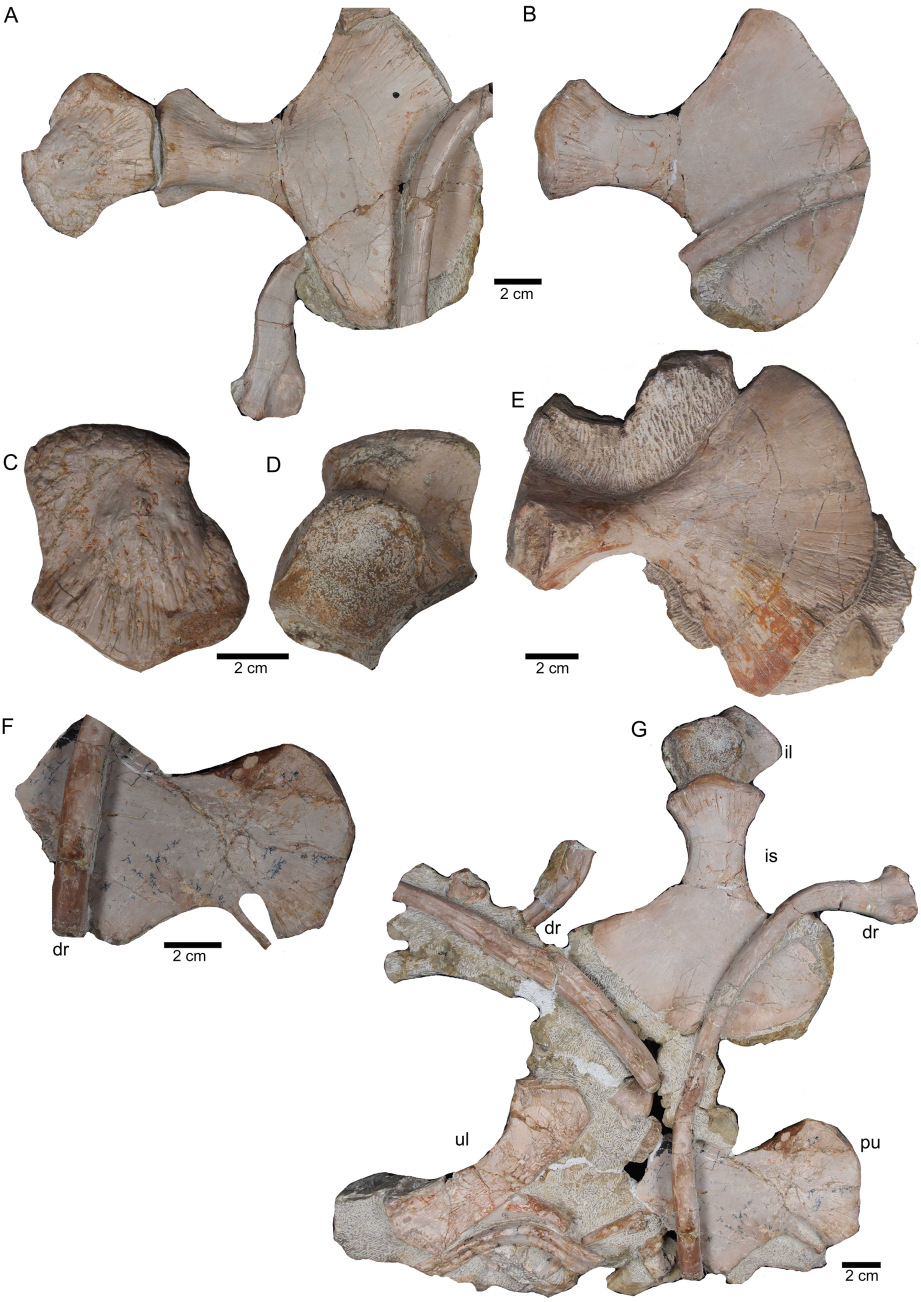
Pelvic elements. (A) Right ischium in dorsal view (field bottom view; slab K); (B) right ischium in ventral view (mirrored) as it is visible in field top view (slab K); (C) Right ilium in dorsolateral view (field bottom view; slab K); (D) right ilium in ventrolateral view (field top view; slab K); (E) isolated left ischium in dorsal view, no connection to the main block; (F) pubis in ventral view slab K dorsal view as it is visible in field top view (slab K); (G) complete slab K in field top view exposing the ulna, ilium, ischium and pubis as well as some dorsal rib fragments.

**Figure 18:**
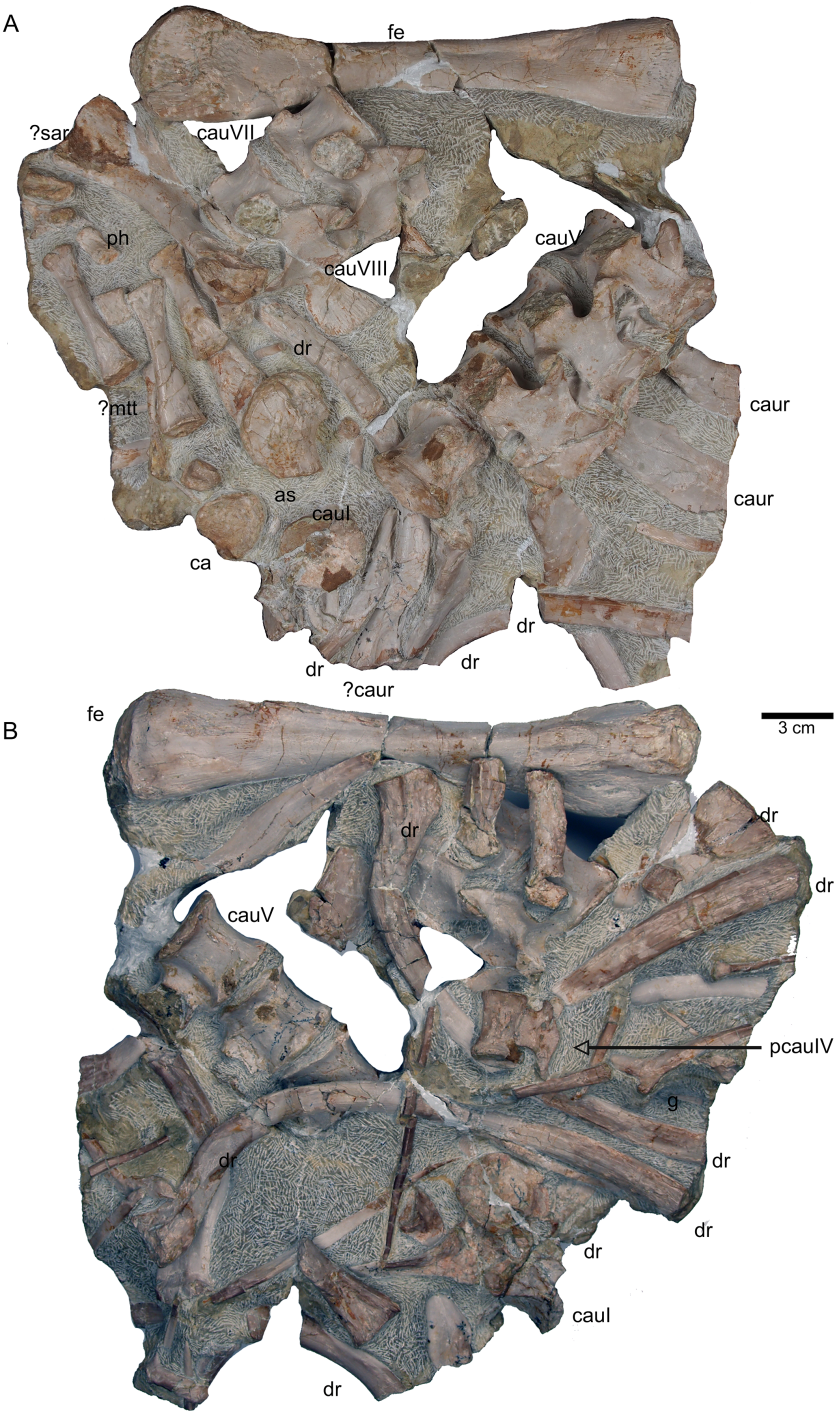
Femur and caudal vertebrae. (A) Complete slab G in field top view, exposing the femur in ventral view, caudal vertebrae II to V from their right lateral side and caudal VII and VIII from their left lateral side, six caudal ribs in dorsal view, elements of the disarticulated pes and two fragments of dorsal ribs. (B) Complete slab G in field bottom view, exposing dorsal ribs and caudal vertebrae I–VII.

**Figure 19:**
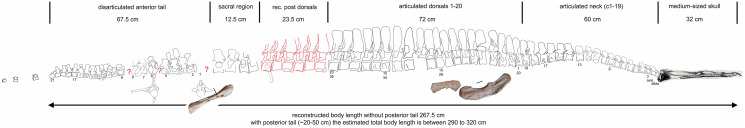
Scaled reconstruction. Scaled reconstruction of UMO 1000. Vertebral column with humerus, ulna and femur and a skull (modified from Rieppel & Wild (1996)/Meyer (1847–1855)) scaled in. Measurements represent the original measured and reconstructed length of vertebral regions.

**Figure 20:**
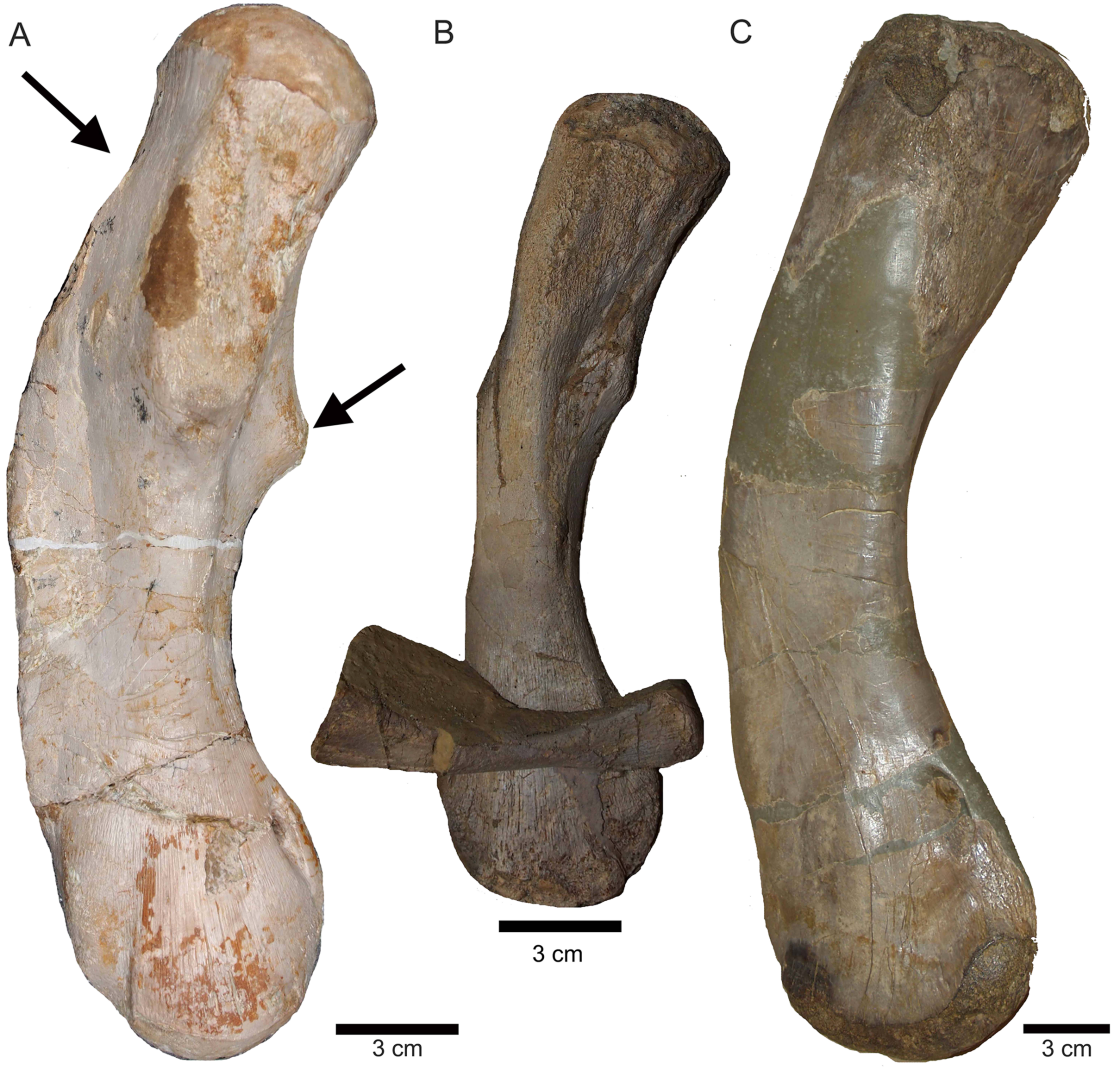
Comparison of humeri. (A) Humerus of UMO 1000, *Nothosaurus mirabilis*; (B) Humerus of *Nothosaurus jagisteus* (Rieppel, 2001), which is partially overlain by a sacral rib. (C) Humerus of *Nothosaurus giganteus* (SMNS 17822/3; Rieppel & Wild, 1996).

**Figure 21:**
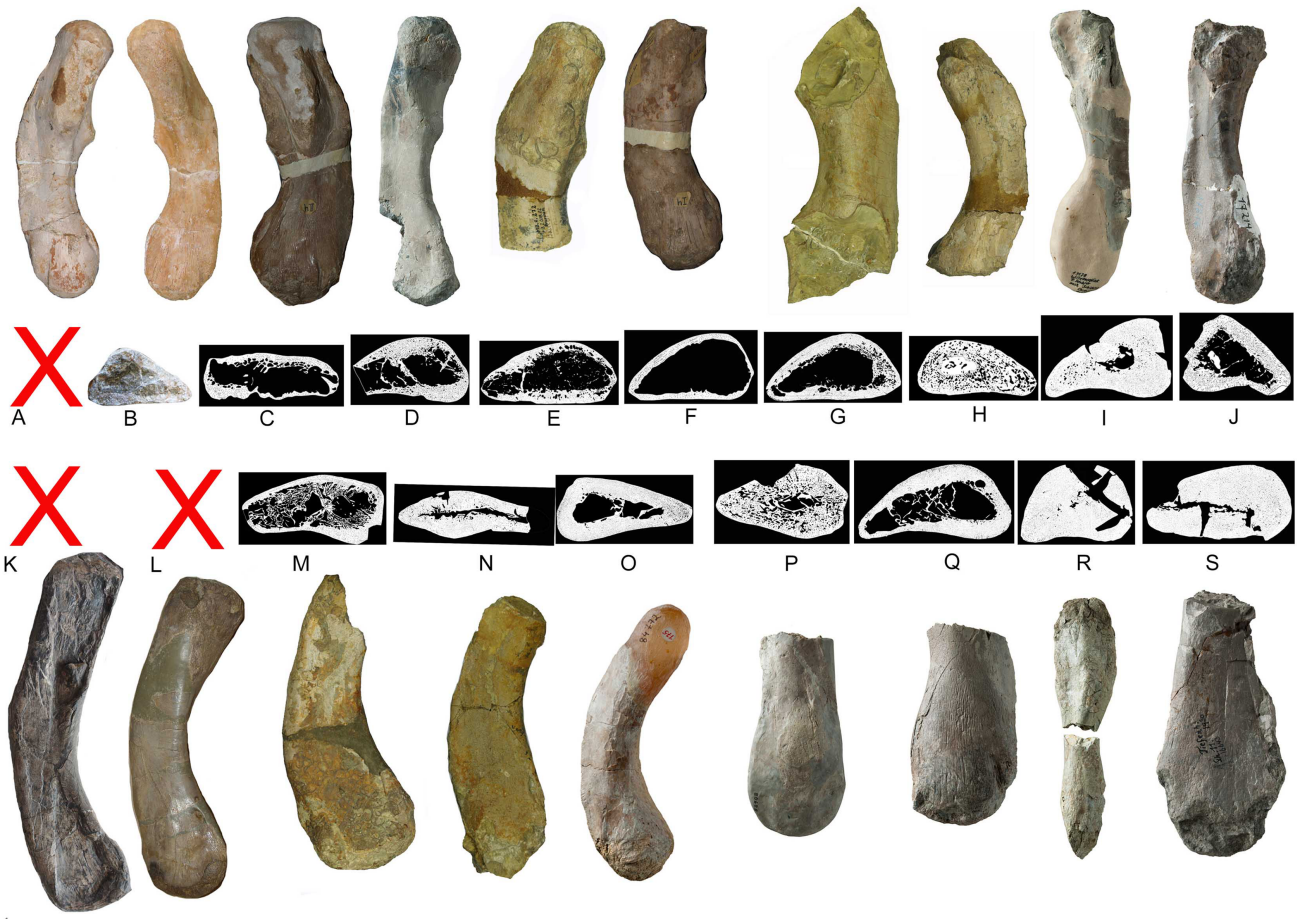
Humeri and microanatomical pattern. Humeri and microanatomical pattern studied in Klein et al. (2016), now assigned to *Nothosaurus mirabilis* (A–J), *Nothosaurus giganteus* (K–O), and those humeri with an osteosclerotic microanatomy (P–S) not clearly to assign due to poor preservation. For locality and other further information see Klein et al. (2016). (A) *N. mirabilis*, holotype UMO 1000 (23.5 cm); (B) *N. mirabilis* with a reduced cortex, UMO, unnumbered (25 cm); (C) *N. mirabilis* with a reduced cortex, PIMUZ AIII-2 (~35 cm); (D) *N. mirabilis*, with a reduced cortex, SMNS 17882 (32 cm); (E) *N. mirabilis* with a reduced cortex, MB.R. 272 (30.5 cm); (F) *N. mirabilis* with a reduced cortex, PIMUZ AIII-2 (30 cm); (G) *N. mirabilis* with a reduced cortex, MB.R. 278 (25 cm); H, *N. mirabilis* with a spongious cortex, MB.R. 282 (23.6 cm); (I) *N. mirabilis*, with a thick cortex, MHI 1978 (18.3 cm); (J) *N. mirabilis*, with a reduced cortex, SMNS 17214 (16 cm); (K) *N. giganteus*, holotype o exhibition at PIMUZ; (L) *N. giganteus* (SMNS 17822/3); (M) *N. giganteus* with a medium thick cortex, MB.R. 269 (40 cm); (N) ?*N. giganteus* with a compressed medium thick cortex, SMNS 80688 (29 cm); (O) *N. giganteus* with a medium thick cortex, MB.R. 281 (27 cm); (P) *N. giganteus* with a spongious cortex, SMNS 84772 (16.5 cm); (Q) *Nothosaurus* sp. with a thick cortex, SMNS 84851 (18 cm); (R) *Nothosaurus* sp. with a thick cortex, MHI 7175 (21 cm); (S) *Nothosaurus* sp. SMNS 81988 (31 cm). Not to scale.

**Figure 22:**
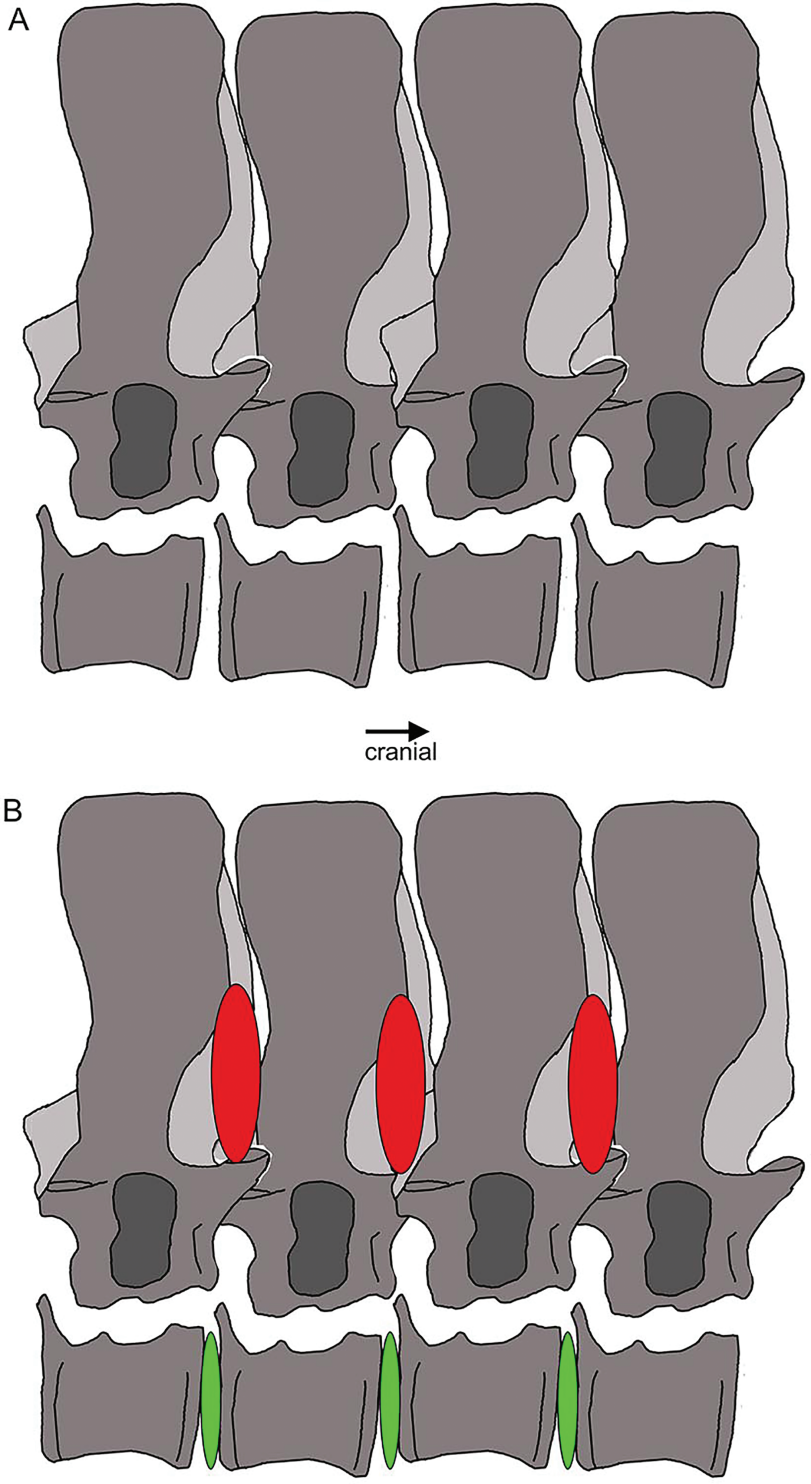
Vertebral stiffening and flexibility. Outline sketch of dorsal vertebrae 18–20 with the supersized zygantrum-zygosphene articulation highlighted. The red ovals indicate stiffening from the neural arch to mid-neural spines, whereas the green ones between the centra mark the large intercentral spaces, likely covered by intervertebral cartilage discs in the living animal, indicating flexibility.

#### History of research of UMO 1000

In March 1834, a worker brought a Muschelkalk slab with bone fragments from the Oschenberg quarry near Laineck to Count Münster, who realized that the fresh fractures indicated the presence of several bones. Instantly Münster checked the locality and spotted the matching counterpart in the quarry wall ca. 8.5–11.7 m below the surface. Münster let the overlying beds remove and excavated most of the specimen. The procedure of the excavation is described in Münster (1834) in the same paper where he also briefly mentioned the specimen and named it *Nothosaurus mirabilis*. Münster (1834) mentioned the vertebral column with ribs and gastralia, parts of the pelvic girdle, fragments of the anterior and posterior extremities, and the anterior part of the lower jaw with some small teeth laterally and larger teeth in front, which are thick, slightly curved and only faintly striated Münster (1834: 525). A skull was not associated with the skeleton (Münster, 1834). Münster (1834) was aware of the importance of this specimen but misinterpreted some elements due to the lack of comparable material at that time. He confused the preserved cervical and tail vertebrae, which was likely due to their weird position in the preserved skeleton (Figs. 5, 6). In addition, Münster (1834: 524) confused humeri and femora. These mistakes had already been corrected by Meyer (1847–1855). Münster donated the specimen to the ‘Kreis-Naturalien-Sammlung zu Bayreuth’ (today UMO) under the prerequisite that it has to stay in this collection and must not be sold (Münster, 1834; Meyer, 1847–1855). Thanks to this requirement, the specimen is still available. The skeleton was soon after its excavation incorporated into a composite mount for exhibition at the museum (Braun, 1840) (Fig. 3). It was put into a plaster bed, arranged with a skull and additional bones of several other individuals to reveal a more complete skeleton. Some original bones of UMO 1000 had been incorporated anatomically incorrectly (Fig. 3). The specimen was figured in its new assemblage in the catalogue of the museum’s collection for the first time (Braun, 1840). A few years later Hermann von Meyer studied this composite and published the first detailed description of the skeleton in his magnificently illustrated folio compendium on Muschelkalk reptiles (Meyer, 1847–1855). However, he illustrated only the vertebral column because he was aware that bones not belonging to this individual (such as the skull) had been added and other elements that might indeed belong to the individual had been inserted anatomically incorrectly or were not included (Meyer, 1847–1855: 29) (Figs. 3B, 3C). For example, the ilium and the lateral half of the ischium had been incorporated as zeugopodial elements in the right forelimb, whereas the medial fan-shaped half of the ischium was positioned above the left humerus as remnant of the shoulder girdle (Fig. 3C). Thus, in his description Meyer (1847–1855) relied mainly on the vertebral column, which undoubtedly belongs to the same individual due to its state of articulation. Other bones of the mount were mentioned by Meyer only briefly, pointing out that their association with the vertebral column might be questionable (Meyer, 1847–1855).

The specimen was over the years mentioned in many publications, *e.g*., in Peyer (1939). This author described *Paranothosaurus amsleri* (*i.e*., *Nothosaurus giganteus* see Rieppel & Wild, 1996) from the Grenzbitumenzone of Monte San Giorgio (Besano Formation) and briefly referred to the ‘Bayreuther Wirbelsäule’ (*i.e*., Bayreuther vertebral column; Peyer, 1939: 3) for comparison. Neither Rieppel & Wild (1996) nor Rieppel (2000), who did a comprehensive review on the genus *Nothosaurus* and on Sauropterygia in general in the late 1990s (summarized in Rieppel (2000)), were able to study the original material of the holotype skeleton of *Nothosaurus mirabilis* because at that time it ‘has been packed for storage for decades and has not been available for study’ (Rieppel & Wild, 1996: 73). In all publications, the description and illustrations of Meyer (1847–1855) served as the basis for any morphological inferences of that skeleton.

#### UMO 1000 in the year 2022

The historical composite skeleton figured in Braun (1840) (Figs. 3B, 3C) was disassembled in 2009 and the postcranial skeleton that was included into this mount was reworked and re-assembled in original find position by one of us (SE) (Fig. 4). In the process of re-mounting, bones not belonging to the original skeleton were identified and have been removed (Fig. 3C), but also, original bones belonging to the skeleton removed in the late 1830s (Fig. 4A) have been re-discovered in the collection and were reunited with the holotype (Fig. 4C). This was possible, because most of the bones of UMO 1000 were still embedded in the original sediment slabs that were covered under the plaster or artificial matrix of the composite mount. Identification of additional bones belonging to the skeleton was possible, due to the bright gray colour of sediment in which bones of UMO 1000 are embedded. Thus, the matrix of pieces belonging to UMO 1000 can easily be distinguished from the yellowish lumachelle matrix attached to isolated bones from the same quarry. The identification of further elements belonging to the original skeleton was also facilitated when fitting to any of the preserved slabs or bones forming the entire block holding the skeleton (Figs. 4C, 5). Today UMO 1000 consists of 13 connected slabs (Fig. 5; slabs A–M) displaying bones at both sides as well as of one additional small slab containing two articulated sacral vertebrae devoid of immediately fitting fractures but matching in color, morphology, and size. The individual slabs mounted on a metal frame and not glued or cemented can easily be removed from the block and the morphology of most elements of UMO 1000 can be examined in any detail (Fig. 4C). In addition, the entire specimen is mounted above a mirror (Fig. 4C), which will allow visitors of the Urwelt-Museum Oberfranken the sight at the bones from nearly all sides in the planned exhibition.

#### Find position and completeness of UMO 1000

It cannot be finally determined, which side of the specimen was field bottom and which was field top. However, there is evidence that the top side of the new mount corresponds to the field top position (Figs. 4C, 5A). According to Münster (1834: 523–524), who excavated most of the skeleton personally, the ribs and gastralia were lying below the vertebral column in a compressed and highly fragmented way, and still stuck in the sediment. Hence, it was not possible to count or excavate them all. This indicates that the ribcage was oriented towards field bottom and would fit to how the specimen is mounted today, *i.e*., with the vertebral column in right lateral view. Thus, in the following, we refer to the top of the new mount as field top position (Figs. 4C, 5A, 6).

Münster (1834) gave a short inventory of the find rather than a morphological description and unfortunately nothing was figured or illustrated. As mentioned above, Münster (1834) confused some bones, and other elements he mentioned are no longer trackable. The coracoid mentioned by Münster (1834: 523) is likely identical with the preserved ulna, the fan-shaped part of the ischium, or it is lost today. Undoubtedly considered to be lost today are the anterior part of a lower jaw fragment, associated lower limb bones (or Münster confused them with sacral or tail ribs) as well as the other femur and ilium that had been mentioned by Münster (1834). On the other hand, he did not mention the well preserved and nicely exposed ischium, likely because its lateral and medial halves were broken and separated. Meyer (1847–1855: 31) mentioned five, not articulated, dorsal vertebrae that had been incorporated into the composite (Fig. 3B) and discussed that these likely belonged to the specimen. However, the matrix associated with these isolated vertebrae differs from the slabs containing the skeleton. Hence, the association of these five dorsals is doubted. The matrix of a small slab containing two articulated sacral vertebrae, one of them associated with its broken sacral ribs and the last right dorsal rib or first sacral rib, fits well as do the elements in size, position and morphology (see below). Thus, it is likely that this slab belongs to the skeleton, too. However, neither Münster (1834) nor Meyer (1847–1855) mentioned any sacral vertebrae. An isolated left ischium (without any matrix preserved) fits in morphology, position, and size to UMO 1000.

#### The status as holotype of UMO 1000

The International Code of Zoological Nomenclature (ICZN) and the type concept were not in use at the time of Münster and Meyer. The ICZN was compiled in 1895 (Blanchard, 1889) and then published and established in 1905 (Blanchard, Maehrenthal & Stiles, 1905). Münster did not illustrate or figure any part of the specimen when he named it as *Nothosaurus mirabilis*. The postcranial skeleton UMO 1000 was explicitly designated as holotype of the taxon *Nothosaurus mirabilis* by Rieppel & Wild (1996) and the skulls found at the same locality became paratypes. Rieppel & Wild (1996) referred to the illustrations in Meyer (1847–1855) for justification. According to ICZN paragraph § 73.1.1, UMO 1000 was correctly designated as holotype for the taxon *Nothosaurus mirabilis* by Rieppel & Wild (1996), because Münster clearly referred only to this postcranial skeleton (later catalogued under the repository number UMO 1000) when writing about *Nothosaurus mirabilis* (Münster, 1834).

### Methods

Measurements (Table 1) were taken with a caliper. In the text and figures, Arabic numbers refer to the correct anatomical positions of the articulated vertebrae of the neck and the anterior trunk region. Roman numbers refer to isolated or disarticulated elements without any reference to their anatomical position. The single slabs forming the entire block were distinguished by letters (A–M). Slab E (Fig. 5) was µct scanned with a v|tome|x s scanner manufactured by GE phoenix|X-ray (Wunstorf, Germany). The µct machine is operated by the Institute of Geosciences, Paleontology, at the University of Bonn (Bonn, Germany). Voltage and current were set to 100 kV and 120 μA, respectively, voxel size was 121 μm. Due to the dimensions of the block, a higher resolution was not possible with this machine.

**Table 1:** Measurements (in cm) of UMO 1000.

Vertebrae	Total length	Total height	Vertebrae	Total length	Total height
atlas (c1)	2.8	4.4	acauI	>3.2	7.5
axis (c2)	3.4	4.6	acauII	nm	nm
c3	3.6	4.9	acauIII	~4	6.1
c4	2.9	5.2	acauIV	3.6	6.1
c5	nm	nm	acauV	4.2	>6
c6	3.7	5.8	acauVI	4.2	>6
c7	3.9	6.1	acauVII	4.3	6.5
c8	4.1	5.9	acauVIII	4.05	7.6
c9	4.1	6.1	acauIX	>4.5	>6
c10	4.2	6.3	acauX	4.9	7.1
c11	4.2	6.5	acauXI	nm	nm
c12	4.2	>6.8	acauXII	nm	nm
c13	4.7	7	acauXIII	3.3	7.4
c14	4.9	7.8	acauXIV	3.8	7.4
c15	4.8	8.1	acauXV	3.9	7.1
c16	4.6	8.6	acauXVI	3.2	7.3
c17	5.3	9.2	acauXVII	3.8	7.7
c18	>5	10.8	acauXVIII	3.4	7.4
c19	4.5	11.7	acauIXX	3.2	6.7
d1	4.4	12.3	acauXX	3.6	6.6
d2	4.6	12.6	acauXXI	3.7	6.1
d3	5.2	13.2	acauXXII	nm	nm
d4	4.4	13.8			
d5	4.1	13.6	**appendicular bones**	**length**	
d6	3.7	13.8	humerus (ri)	23.5	
d7	3.8	13.9	humerus (le)	22.8	
d8	4.1	14.4	ulna	11.3	
d9	4.1	14.1	?radius	10	
d10	3.7	14.4	femur	21	
d11	3.5	14.6		**length**	**width**
d12	3.5	15.1	ilium	4.85	5.9
d13	4.1	15.3	ischium (slab K)	12.3	12.6
d14	4	15.1	ischium (isolated)	13.5	12.4
d15	4.1	15	pubis	11.5	7.5
d16	3.7	14.8			
d17	3.7	14.4			
d18	4.7	13.9			
d19	3.6	13			
d20	3.8	13.1			
sa1	>3.5	nm			
sa2	>3.1	nm			
sa3	>3.6	>7			
sa4	3.71	7.1			

**Note:**

Please note that due to compaction and slightly different angles in preservation mainly the measurements of vertebrae are not very accurate. Abbreviations as for figures. Discrepancies in measurements to Meyer (1847–1855) may result from different method and further preparation of the specimen. The total length refers here to the length including the dimension of the zygapophyses and the height extends from the broadest centrum margin to the tip of the neural spine (Alafont 1992: fig 3.2).

## Results

**Systematic Paleontology**

Sauropterygia Owen, 1860

Eosauropterygia Rieppel, 1994

Eusauropterygia Tschanz, 1989

Nothosauridae Baur, 1889

*Nothosaurus* Münster, 1834

Type species *Nothosaurus mirabilis* Münster, 1834

**Holotype – **Partial postcranial skeleton (UMO 1000); original of Meyer, 1847–1855, Pl.

23.

**Paratypes –** (all skulls) UMO BT 667,00; 671,00, originals of Meyer, 1847–1855, Pl. 2, Figs. 1–2 and Pl. 3, Fig. 1; Pl. 3, Fig. 2 and Pl. 4, Figs. 1–3 (Fig. 47–50 in Rieppel, 2000).

**Stratum Typicum –** Upper Muschelkalk, Trochitenkalk or lower Meißner formations (moT, moM); atavus through compressus biozones; Middle Triassic, late Anisian, latest Illyrian (Rieppel, 2000).

**Locus Typicus –** Oschenberg near Laineck (also referred to as Lainecker Berg or Lainecker Höhenzug), East of Bayreuth, Bavaria, Germany.

**Diagnosis** (Rieppel, 2000) **–** A species of *Nothosaurus* of intermediate size with an adult condylobasal skull length of up to 460 mm; rostrum long and slender with parallel lateral edges; length-to-width ratio of mandibular symphysis 1.5–1.7; five fangs on each premaxilla; four small maxillary teeth preceding the paired maxillary fangs; rostral constriction weakly expressed; external nares long and slender; upper temporal fenestra elongated, with a constricted anterior corner and with the maxillary tooth row extending backwards to a level below its midpoint; high neural spines on dorsal vertebrae.

**Emended diagnosis** (this study) **–** The species is further characterized by a supersized zygantrum-zygosphene articulation connecting the high neural spines of the dorsal vertebrae; large intercentral spaces between centra in the neck and anterior trunk region; a flat humerus with an extremely broad (not constricted) shaft; humerus with a prominent edge at the beginning of the proximal postaxial shaft margin and a thin but broad crest that forms the preaxial half; flat ulna; humerus/femur ratio is 1.12; femur straight, ilium lacks a distinct set off iliac blade; ischium symmetrical; pubis with a not well pronounced prepubis and a obturator foramen in form of a deep slit.

**Distribution –** Upper Muschelkalk and Lower Keuper (Middle Triassic, late Anisian through early Ladinian); Central Europe, southern Alps.

**Redescription of the holotype (UMO 1000) of *Nothosaurus mirabilis***

**General description of UMO 1000 –** The holotype of *Nothosaurus mirabilis* (Münster, 1834; Rieppel & Wild, 1996; Rieppel, 2000), UMO 1000, consists of a partially articulated vertebral column with some of the limb and pelvic girdle bones closely associated. All elements fit in size, side, and number, indicating a single individual. Most parts of the vertebral column are exposed from their right lateral side in field top view (Figs. 4C, 5A), although some strings of articulated vertebrae are exposed from the left side in field top view (Figs. 5B, 6). However, most vertebrae are visible from both sides (Figs. 4C–14). The preserved limb and pelvic girdle bones are exposed in ventral or dorsal view (Figs. 6, 15–17). Pre-burial movements of the skeleton are indicated by the loss of some elements as well as by the unusual anatomical position of other elements and articulated parts of the vertebral column (Figs. 5, 6; see below).

The following description refers to the presumed field top view of elements (Figs. 4C, 5A). The anterior part of the vertebral column was turned between the 13^th^ and 14^th^ cervical vertebra and rotated almost 180°, resting now upside down parallel to the posterior cervicals and anterior dorsals. It is thus disarticulated from the rest of the neck (*i.e*., the posterior cervicals) and the anterior trunk column (Figs. 5, 6). The anterior part (*i.e*., atlas/axis *etc*.) points caudally, exposing still the right lateral side, and, thus, the centra are lying close to the centra of the rest of the articulated vertebral column, which displays the right lateral side. The 14^th^ to 19^th^ cervicals as well as the 1^st^ to 20^th^ dorsals are articulated and expose their right lateral side. The posterior part of the dorsal vertebral column is lost. Two articulated sacrals associated with both their pairs of sacral ribs (slab H) have a clear fit *via* their corresponding ribs to the main block (slab I), lying in ventral view close to the proximal head of the shifted left humerus (Figs. 5A, 6). Two additional sacrals have no fit to the main block but likely belong to the same individual (see above). The posterior one of them is nearly complete, also still associated with its broken off ribs at each side. From the anterior sacral vertebra, only the neural arch is preserved. There is space on the same small slab for the preceding vertebra, which we interpret as last dorsal of which the right rib is still in place (Fig. 11A). The numbering of the (preserved) caudal vertebrae follows—besides morphological characters (*i.e*., size and morphology of rib facets and presence of haemapophyses)—roughly their decreasing size (Figs. 11, 12; Table 1) but it cannot be excluded that caudals in between are lost or that the number or order respectively is incorrect. This is because of the disarticulation but also due to compaction and preservation in different views that obscures correct measurements. Nine caudal vertebrae, which are interpreted as anterior caudals (see below), are partially articulated (caudals I–V; caudals VII and VIII) or isolated (caudals VI and IX), respectively, and are spread over the posterior part of the block (Figs. 5, 6). The string of five and the two articulated caudal vertebrae are associated with the disarticulated and incomplete right hindlimb (Figs. 5, 18). These vertebrae are interpreted as anterior caudals, due to their size (Table 1) and morphology, indicating a position close to the sacral region. Further on, they are associated with broad and relatively short caudal ribs (Figs. 5, 12C, 12F, 12I, 12K, 18A). In field top view, only the centrum is visible from caudal I (Figs. 5A, 12A–12C) but it is more or less complete in field bottom view (Figs. 5B, 12A). Caudal II lacks the neural arch and spine. Caudals III–V are complete and are articulated in a row (Figs. 5A, 12C). Between preserved caudals I and II is enough space for another—now lost—caudal or they are just separated. The row of caudals I to V and caudals VII to VIII lay roughly parallel to each other, separated by some centimeters and running perpendicular to the main part of the vertebral column (Figs. 5A, 18). The isolated caudal VI is exposed in anterior view (Figs. 5A, 12G). The right transversal process of caudal VI is articulated to a caudal rib and on the left ventral side to a haemapophysis. Caudals VII and VIII expose their left sides in field top view and both show distinct articulation facets for the haemapophyses (Fig. 12H). At their right side they are still articulated to the proximal part of their caudal ribs (Fig. 12I). Caudal IX is exposed in posterior view, lying close to the distal end of the right humerus (Figs. 5A, 12J–12L). A string of 13 articulated caudal vertebrae, exposed in left view, run over two slabs (slab E–F). Slab E contains caudals XVI to XXII (Figs. 5, 10) and slab F contains caudals X to XV (Figs. 5, 13). The string of 13 caudals was turned, *i.e*., the posterior part is pointing anteriorly – and lying in the gap behind the preserved anterior dorsal column (Figs. 5, 6, 10). Caudals IXX to XXII are obscured by dorsals 18–20 (v37–39) but they are visible from the field bottom side (Figs. 5, 10B). Additionally, four isolated, small caudals of the posterior tail region are scattered over the block (Figs. 5, 14A–14E). The cervicals and anterior dorsal vertebrae are complete, meaning that centrum and neural arch and spine are articulated. The dorsals 11 to 20 are separated along their neurocentral suture by a few millimeters (Figs. 9, 10), indicating poor ossification. The articulated parts of the entire preserved vertebral column indicate large inter-central spaces, leaving about 0.5 cm between each centrum and implying a thick layer of cartilage between the centra.

Three cervical ribs are still articulated to their corresponding cervicals (cervical ribs 9, 11, 12) (Figs. 5, 7, 14F–14M). Five cervical ribs are found isolated and spread over the block (Fig. 5, 14F, I_M). About 13, fairly complete dorsal ribs (Figs. 5, 14O, 14Q, 14R) and several fragments of dorsal ribs (Fig. 5) are visible below and aside the vertebral column in field top and field bottom view. However, most dorsal ribs and their fragments are visible in field bottom view (Fig. 5B). Four sacral ribs are still attached to their two corresponding sacral vertebrae (Figs. 5, 11E, 11F, 11H). Two broken off sacral ribs are associated with sacral II (see below) and maybe one or two sacral ribs are found isolated (Figs. 5, 14T). However, it is difficult to address the exact anatomical assignment of some of the ribs due to incompleteness and compaction. Three distinctly flattened caudal ribs are associated with the left side of the caudals I–V (Figs. 5, 12C). The middle one between the three ribs lying between caudal I and II, is a dorsal rib (Figs. 12C, 14S, 18A). The two other ones may be caudal or dorsal ribs. The posterior one has a crest and is heavily flattened (Figs. 5A, 14S). One isolated short rib associated with the anterior neck at slab A (Figs. 5, 7A, 14N) might represent a posterior cervical or anterior dorsal rib. Another isolated rib associated with the pubis on slab K (Figs. 5B, 14P), might represent a posterior dorsal or anterior caudal rib. Gastralia are all disarticulated. Only few median elements are preserved (Figs. 5, 13D, 13E), most fragments represent lateral elements (Figs. 5, 13F). Their fragments are mainly found on the field bottom side of the specimen (Fig. 5B).

The right humerus is in field top view exposed in ventral view (Figs. 5, 6, 15A–15D). It is slightly dislocated caudally (when considering the position of neck and anterior trunk region based on the preserved vertebral column) and lies at the height of the mid-trunk region. Its distal end points anatomically correct in caudal direction. The left humerus is in field top view exposed in dorsal view (Figs. 5A, 15E, 15F) and has shifted even further caudally, lying now at the region of the formerly posterior trunk or even sacral region. The element has turned and its distal end points now cranially. The ulna has its distal end close to the atlas and the shifted part of the anterior neck but it is still in the region of its anatomical correct position (Figs. 5A, 15A). Associated with the left humerus is one single phalange (Figs. 5A, 16J). The preserved right femur is visible in dorsal view in field top view and lies at the posterior part of the skeleton at the same height as the left humerus and the two sacral vertebrae (Figs. 5, 7, 15G, 15H, 18). The distal end of the femur points cranially. The same slab contains remains of a disarticulated pes (in dorso-ventral view), consisting of three to four tarsals, four to five metatarsals (elements overlie each other) and two phalanges (Figs. 5A, 16I, 18A). Three additional elongated limb bones, either representing metacarpals or metatarsals, are spread over the block (Figs. 5, 16B, 16F, 16J, 16K). No elements of the shoulder girdle have been identified. The disarticulated but associated right pelvic girdle lies at the height of the anterior trunk region (Figs. 5, 17G). The right ischium and ilium are still associated (although the ilium was shifted) and exposed in ventral and medial view, respectively (Figs. 5, 17). The pubis was also turned and lies still close to the vertebral column. An isolated—left—ischium fits in size, morphology and side to the skeleton (Fig. 17).

The vertebral column is laterally compressed, whereas some caudal ribs and the left humerus are strongly dorsoventrally compressed. Many of the dorsal ribs show locally compaction along their midshaft.

**Skull –** No cranium was found with the postcranium except for an anterior part of a lower jaw (Münster, 1834; see above), which seems to be lost today. At least it could not be located yet in the UMO collection, despite meticulous efforts to find all missing pieces of the holotype by one of us (SE). According to Münster (1834), the jaw fragment showed the typical nothosaur dentition: small teeth laterally and large fangs in the front, which are only slightly curved and striated. Unfortunately, this piece was never figured and was also not mentioned by Meyer (1847–1855). The skull included to the historical composite skeleton (Braun, 1840) belonged to a different individual (Meyer, 1847–1855). Meyer (1847–1855) stated that the atlas would fit to a medium sized skull (~32 cm; Meyer, 1847–1855: 32) out of the numerous skulls found in the vicinity of Bayreuth and which were assigned to *Nothosaurus mirabilis*. It has to be noted that the skulls—on which most of the diagnosis of Rieppel & Wild (1996) and Rieppel (2000) for this taxon is based–are all paratypes. No skull or jaw material associated to UMO 1000 is known today.

**Vertebrae –** The cervical vertebral column of UMO 1000 is complete, consisting of altogether 19 vertebrae (Figs. 4C, 5–8). It is preserved in two articulated strings. Both strings, forming the entire neck, measure together ~60 cm (Fig. 19). All cervical centra are firmly attached to their corresponding neural arches (Figs. 7, 8). The atlas (Figs. 7A, 7B) has a low, nearly reduced and triangular-shaped neural arch, with the acute angle pointing posteriorly, forming a kind of ‘postzygapophysis’. The atlas is nearly half the size and height of the axis, the centrum being higher than long and somewhat constricted. The ventro-posterior margin of the centrum forms a posterior beak, paralleling the posterior part of the neural arch. Its anterior facet is slightly concave to receive the condyle. The posterior facet and the ventral margin are straight. There is no trace of an articulation facet for a cervical rib on the atlas (which is contrary to the description of Meyer (1847–1855)). The centrum of the axis (Figs. 7A, 7B) is nearly twice as long than high with its posterior ventral margin being stronger concave than in the posteriorly following cervicals. The massive neural arch, which is nearly twice as heigh as the centrum, differs from that of all other vertebrae: the spine is antero-posteriorly elongated and convex. The area is crushed but there seems to be a steep, round ridge, resembling a prezygapophysis running below the ‘postzygapophysis’ of the atlas. The postzygapophysis of the axis is well developed. The axis already resembles the morphology of the posteriorly following cervicals. Atlas and axis were in right side view separated by sediment and are not fused (Figs. 7A, 7B) but in left view they are closely together (Figs. 7C, 7D). Hence, it cannot finally be clarified if atlas and axis were fused or separate. The area where a possible cervical rib could have been attached to the axis is damaged. The cervicals 3 and 4 are similar in appearance to the axis, with the neural spines strongly reclined and pointing posteriorly, although their neural arches lack the anterior process present in the axis. The neural arches are steep with the posterior side pointed and higher than the anterior side. In both cervicals the centra are more massive and increase in height but remain longer than high and are less but more regularly concave ventrally. Both centra have two rib facets on either side, which are, however, very close to each other. The neural arch of cervical 5 is not preserved. From cervical 6 onwards the cervicals are of rather uniform morphology but continuously increasing in size (*i.e*., in height and length). The generally dorso-ventrally oval centra of the cervicals are laterally constricted and ventrally keeled by a very pronounced but narrow ridge (Fig. 7H). On both sides of these keels are depressions or channels, which contain foramina in some of the vertebrae. Pre- and postzygapophyses are in cervical 3 shifted, with the latter being now positioned higher in dorsal direction. The apophyses from cervical 4 backwards run nearly parallel, *i.e*., horizontally and are located approximately at the same height, along the entire neck (Fig. 7). The neural arch of cervical 8 shows a quite large foramen lateral to the neural canal, postero-ventrally of the prezygapophysis. This foramen is also observed in cervical 7 and 9, although being smaller and thus appearing less pronounced. It is not observed in the other cervicals. In the mid-cervicals, the anterior half of the neural arch is in dorsal view broader than the posterior half (Fig. 7I) but this is *vice versa* in the posterior cervicals (Fig. 8C). The zygosphene-zygantrum articulation is well developed (Fig. 7E). The top of the neural spines is posteriorly pointed from the axis to cervical 4, then they become broader and the neural spine parallels the dorsal margin of the centra. In the posterior-most cervicals (cervicals 18–19), the tops of the neural spines become postero-dorsally inclined (anteriorly dipping down). In general, the neural spines of the cervicals remain relatively low, only slightly exceeding the height of the cervical centra in the anterior to mid-neck region (Table 1). From cervical 14 backwards, the height of the vertebrae increases distinctly, which is mainly due to an increase of the height of the neural spines (Figs. 5A, 7A, 8).

All cervical centra (except for the atlas) have two rib facets that are principally horizontally oriented (Figs. 7A, 7B). Because in some cervicals the cervical rib is still attached but also due to compression the two rib facets are not always well visible. In the anterior cervicals (cervicals 2–12), the rib facets are located in the mid-ventral half of the centrum (Fig. 7B). The rib facets start anteriorly as round knobs, which become continuously larger. The facets lie horizontally and are situated closely together and thus are often not well to distinguish. In cervicals 3 and 4 the upper facets are much more pronounced than the lower ones. In cervical 8 the size difference of facets has matched. In the following cervicals, the rib facets are more clearly separated and the upper one (diapophysis) has moved dorsally closer to the suture to the neural arch. From cervical 14 backwards, the upper rib facet lies on the neural arch and the lower (parapophysis) one on the centrum (Figs. 8A, 8B). The facets are now nearly equal in size. In the posterior cervical vertebrae, the facets become more protruding, approaching the appearance of the transversal processi. In cervical 17, both facets form most of the lateral side of the mid-centrum, with the ventral parapophysis being now the larger one. Both facets are clearly separated by a distinct channel. The position and morphology of the rib facets changes quite abruptly in cervicals 18 and 19 (Figs. 8D, 8E). In cervical 18, the diapophysis forms a steep dorsoventrally running rectangle and has moved to the posterior margin of the ventral neural arch. The elongated rectangular parapophysis is very massive, forming most of the lateral centrum. Both rib facets are clearly separated from each other. In cervical 19, the diapophysis is constricted and larger than in cervical 18. Please note that in this study cervical vertebrae are distinguished from dorsal vertebrae by their number of rib facet: cervicals have two rib facets that are clearly separated by a channel. According to this definition we counted 19 cervicals, which is contrary to Meyer (1847–1855) who counted 20 cervical vertebrae.

The anterior dorsal column is in articulation from the first dorsal (*i.e*., the 20th vertebra in row) to the 20th (*i.e*., the 39th vertebrae in row) (Figs. 5, 8–10). Some dorsals are damaged, *i.e*., have the neural spine broken off (dorsal 4/v23, dorsal 5/v24) or the centrum is lost (dorsal 12/v31). The anterior-most four dorsals, still distinctly increase in size (Table 1; Figs. 5A, 8D). From dorsal 5 (v24) backwards, the size increase is less obvious but continuous (Table 1; Figs. 5, 8–10). The maximal total vertebral height is reached in dorsal 13 (v32; Figs. 5A, 9A; Table 1). After that, the height decreases again continuously (Figs. 5A, 9A; Table 1). The dorsal centra have slightly concave anterior and posterior articulation facets (*i.e*., they are slightly amphicoelous) with distinctly set off margins (Figs. 8–10). They are round-oval in dorsoventral direction (*i.e*., they are less high-oval when compared to the cervicals) and laterally constricted. The centra of the dorsals have an almost straight ventral margin (contrary to the concave ventral margins in the cervicals). Dorsal centra are ventrally keeled by one broad but less pronounced keel when compared to the centra of the cervicals. The centra of dorsals 14 to 17 each have one or two distinct vertically running scars (*i.e*., deep welts), indicating muscle attachments (Fig. 9A). The neural arches are not very broad or massive and stand with clearly set off pedicals on the centra, leaving room for a large round neural canal. The postzygapophyses are slightly higher than the prezygapophyses but both appear nevertheless nearly parallel and thus horizontally. The neural spines of the dorsals are in general high, being roughly twice the height of centrum and neural arch together (Figs. 5, 8–10). The neural spines had been prone of strong lateral compaction, resulting in a strange morphology: They have a well ossified main part that is postero-ventrally narrow or constricted and broadens in dorsal direction, becoming dorsally nearly twice as wide as ventrally (Figs. 8–10). Antero-ventrally to this well ossified posterior main part, the neural spine has a very thin bony ‘lamina’, which is only 1 mm (if at all) in width, compared to the well ossified part that is about 0.5 cm in width. This thin part is restricted to the anterior half of the neural spine (Figs. 8–10). It is ventrally–as visible–up to 1 cm long but tapers and finally merges dorsally with the well ossified part of the neural spine (Figs. 8–10). This ‘lamina’ was only made visible after further preparation in the course of the re-mounting and had been before covered by sediment. This antero-ventral lamina represents a supersized zygosphene that fits in between the zygantrum of the posterior part of the preceding vertebra (Figs. 8–10), forming an additional articulation for the high neural spines. These anterior ‘laminae’ are present from dorsal 4 backwards to the last preserved dorsal (dorsal 20/v39), although, due to preservation, not clearly visible in each vertebra. The top of the neural spine is always well ossified. The general shape of the neural spine is roughly rectangular in lateral view. Its top becomes more round and more massive contrary to the straight but postero-dorsally inclined (anteriorly dipping down) neural spines of the two last cervical vertebrae and the first two dorsals. From dorsal 16 (v35) onwards the top of the neural spine becomes horizontally straight again. The top of the neural spine shows vertical ridges indicating strong muscle attachments (Figs. 5–7). All dorsals have a single (*i.e*., fused), massive, protruding, roughly 8-shaped rib facet at either side, which is oriented principally vertically (Figs. 8–10). It distinctly projects laterally beyond the zygapophyses. In the first three dorsals (v20–22), di-and parapophyses are already fused but the sutures between both is still visible (Fig. 8D). The ventral part (*i.e*., parapophysis) is still located dorsally on the centrum, whereas the larger upper half (*i.e*., diapophysis) is situated on the neural arch. After the 4^th^ (v23) dorsal, the fused rib facet is located solely on the neural arch. The preserved string of dorsal vertebrae measures 72 cm (Fig. 19).

An unknown number of dorsal vertebrae is missing posterior to dorsal 20 (v39). This is evident by a clear change in morphology and size (*i.e*., height) between the last preserved dorsals and the sacral vertebrae (Fig. 5A; Table 1).

Two articulated sacrals (sacrals I and sacral II) are associated with UMO 1000 (Figs. 11A–11C) fitting in morphology and size to the main block. Their heart-shaped centra are laterally slightly constricted but not keeled. The posterior view of sacral II provided a zygantrum articulation (Fig. 11C). Their neural spines are incomplete but had been low (Table 1). On the same slab is space for a preceding vertebra, which is now lost but the corresponding left rib is preserved (Fig. 11A), being different from the sacral ribs (see below). This rib likely represents the last dorsal or first sacral rib. The anterior position of sacral I and II is supported by higher neural spines when compared to the two articulated sacrals (sacral III and sacral IV) on slab H (Figs. 11E–11I; Table 1). Sacral III and IV are articulated to each other and to their corresponding sacral ribs (Figs. 5, 11E). They have a dorso-ventrally flat oval centrum, which is only slightly constricted and not keeled but smooth. A well-developed, but much smaller (when compared to the dorsals) zygosphene is well visible although dislocated at sacral III in anterior view (Fig. 8H). The posterior view of sacral IV provides a dorsally shifted zygantrum (Fig. 11F). The tops of the low neural spines show muscle scars. The dorsoventrally extensive but laterally not far protruding transversal process is supported by centrum and neural arch, but the bigger part is on the neural arch. Thus, at least four sacrals (possibly 5) are preserved with UMO 1000 but one cannot exclude that additional ones are lost. The four sacrals measure together about 12.5 cm (Fig. 19).

The tail of UMO 1000 is highly disarticulated and incomplete. The anterior caudals share, in anterior-posterior view, round centra (Fig. 12) that are constricted laterally and are keeled (Fig. 9). Posteriorly, the centra become dorso-ventrally more oval (Figs. 10, 13). The neural arches have a low neural spine and pre- and postzygapophyses are horizontally oriented. The prezygapophyses are more prominent than the postzygapophyses (Fig. 12L). The top of the neural spines is striated, indicating muscle insertions. The large round-oval rib facets of caudals I–IX show a suture, which is located approximately at the midline between centrum and neural arch (Fig. 12D). The rib facets are moving ventrally and become distinctly and continuously smaller from caudal X backwards to caudal XVIII (Figs. 10, 13). From caudal XI backwards the rib facets are restricted to the centra. They are nearly absent or reduced from caudal IXX backwards (Fig. 10). From caudal VI backwards until caudal XXI paired haemapophyses are present (Figs. 10, 12, 13). They are situated ventrally at the posterior part of the centrum (Figs. 10, 12H, 13). Four isolated small caudals from the posterior tail region are scattered over the block (Figs. 5, 14A–14E). They are small and low (Table 1), being divided into an oval, constricted and keeled centrum and a reduced neural arch and spine (Figs. 14B, 14D, 14E). No traces of haemapophyses are visible on these posterior caudals. The different parts of the preserved caudal vertebral column measure in sum about 67.5 cm (Fig. 19).

**Ribs –** The right cervical ribs 9, 11, and 12 are still articulated to their corresponding cervicals (Figs. 5A, 7A, 14G, 14H). In addition, up to six isolated cervical ribs have been identified distributed over the block (Figs. 5, 14F, 14I–14M). All cervical ribs are double-headed. In the anterior neck, they are short (nearly oblong) with two lateral processes: a very short anterior and a slightly longer convexly curved posterior one. In the mid-cervical ribs, the posterior process becomes longer (Fig. 14K). Because the cervical ribs are preserved in different views and from different sides, they appear very irregularly shaped (Figs. 14F–14M).

The dorsal ribs have a set off proximal head and are curved shortly behind the rib head whereas the rest of the rib shaft is only slightly curved (Figs. 5, 14O, 14Q, 14R, 17, 18). The proximal head of the rib forms a constricted oval (*i.e*., 8-shaped) in cross section whereas the rest of the rib is of round-oval cross section, sometimes compressed or flattened. The shafts of the ribs are striated. All ribs are incomplete, all lacking the distalmost part, which was likely not well ossified, *i.e*., consisted of calcified cartilage as is the case in some other eosauropterygians (Klein et al., 2015; Klein, Canoville & Houssaye, 2019). The longest and most complete dorsal rib measures about 24 cm (Figs. 5A, 14O, 17G). The massive articulation facets of the rib heads fit the 8-shaped transversal processes of the dorsal vertebrae. Except of their massive proximal heads the dorsal ribs are relatively slender. The number of dorsal ribs associated with the specimen is low, which is the result of an excavation bias (see above; Münster, 1834). Between caudal I and II are three rib fragments with the shafts partially running below caudal II. They are of a strange morphology with distinct crests proximally (Figs. 12C, 14S, 18) and it cannot finally be solved if these represent dorsal or caudal ribs.

According to our interpretation, the rib in front of sacral I and II represents the last left dorsal rib (Fig. 11A). It is standing vertically and pointing ventrally (Fig. 11A). It is short but complete with a massive proximal head but a slender and pointed shaft. However, it cannot be excluded due to ‘sacralization’ of the last dorsals (Rieppel, 1994) that this was the first sacral vertebra. Two ribs (Figs. 14N, 14P) are difficult to assign. One has a massive—maybe two divided—articulation facet, a distinct ridge on the shaft and a slender pointed distal end (Fig. 14N). This could represent either a posterior-most cervical rib or an anterior dorsal rib. The other rib, which is also not exactly to assign, is smaller and overall more slender (Fig. 14P). This could be a posterior dorsal or anterior caudal rib. No ribs are associated to sacral I. Sacral II is still associated with the proximal part of its right and left ribs (Figs. 11A, 11B). Both are broken off and are now standing vertically, with the right rib pointing ventrally and the left rib pointing dorsally. Both show the typical massive and angled head of sacral ribs. The four sacral ribs are articulated to sacrals III and IV (Figs. 5, 11E). They are short (Table 1) and straight, nearly rectangular, and flattened or compacted in dorso-ventral view. The proximal and distal ends are nearly equal in size, with the distal end only being slightly longer. Both ends are very massive and angled in lateral-medial view. In their anterior to midshaft region, three out of the four have a posteriorly pointing half-round crest developed, which, however, is in all differently developed, depending on preservation and view. Their preserved distal ends are hollow (Fig. 11G), indicating a thick cartilage cap. At least one isolated additional sacral rib has been identified, lying together with elements of the disarticulated pes (Fig. 14T).

The three ribs which are associated with caudal vertebrae III to V are dorsoventrally flattened, appearing very flat and broad (Figs. 12C, 14S, 18). It cannot be clarified if they represent dorsal or caudal ribs. The right caudal rib articulated with caudal vertebra VI also has a massive proximal head and a straight shaft. The shaft is slender and tapers distally, which is quite different to the compacted or compressed caudal ribs mentioned above. The caudal rib associated with caudal VI is shorter when compared to the caudal ribs associated with caudals I to V (Table 1). The caudal ribs articulated to caudal VII to IX are incomplete but seem to have been much shorter than the above-described caudal ribs. High variation in the morphology of caudal ribs is also the result of different views and preservation.

**Haemapophyses –** Only two haemapophyses are identified along with UMO 1000 (Figs. 12E, 12G). One left half of a haemapophysis is articulated to caudal VI (Fig. 12G). It has a smooth bone surface and it is latero-medially curved.

**Gastralia –** Numerous fragments of gastralia are present, largely visible at the underside of the new mount (Figs. 5, 13D–13F). Most of them are just fragments, *i.e*., simple rods representing the lateral elements (Fig. 13F) of the gastralia, while a few represent the typical v-shaped median parts with a short anterior process (Figs. 13D, 13E). It cannot be verified if a complete gastralium was built of five elements, although this is likely when considering the condition in other *Nothosaurus* spp. (Rieppel & Wild, 1996; Rieppel, 2000). The single gastral elements and fragments respectively are massive and can be considered pachyostotic when compared to the rest of the bones of UMO 1000. As for the dorsal ribs, the number of preserved gastralia is low in UMO 1000, which is due to an excavation bias (Münster, 1834: 523).

**Girdle bones –** The right ilium is associated to the right ischium but it has slightly shifted (Figs. 17A, 17C, 17D, 17G). The—for sauropterygians—typical small ilium is of rather simple morphology. The iliac blade is reduced and not set off from the rest of the element. Its dorsal margin is slightly convex, with the posterior end minimally tapering and the anterior end (*i.e*., spina preacetabuli) being round. The ventral part of the ilium that participates in the formation of the acetabulum, is broader than its dorsal part. The posterior margin is dorsally constricted (Fig. 17C). Its lateral side is bulged and shows striations, whereas all other surfaces are smooth. The medial side has ventrally two large oval articulation facets: one for the ischium and one for the pubis. Antero-dorsally, above the facet for the pubis, runs a deep channel (Fig. 17D). No articular facets for the sacral ribs are visible. As Rieppel (1994) already pointed out, in *Nothosaurus* spp. is only space for three sacral ribs articulating to the medial ilium. The ischium is symmetrical with a deep concave anterior and posterior lateral margin and a flat, fan-shaped medial part (Figs. 17A, 17B, 17E). The medial margin is bifid but symmetrical. The shaft towards the lateral part is constricted but massive and relatively short. The isolated (left) ischium exactly corresponds in morphology with the ischium on slab K (Fig. 17E). The pubis has concave anterior and posterior margins (Fig. 17F). Although covered by a dorsal rib, a weak prepubic process can be identified. The most striking feature is a deep slit-like obturator foramen.

**Limb bones –** Both humeri are preserved and visible from all sides (Fig. 5). The right humerus is three dimensionally preserved (Figs. 15A–15D) whereas the left humerus is flattened and crushed (Figs. 15E, 15F). The humerus has a massive proximal head but shaft and distal end are very flat. Proximal and distal ends are not twisted against each other and both are of a similar width. The shaft is broad and not constricted. The preaxial (lateral) margin is long and straight to slightly convex and in lateral view very thin. In dorsoventral view it appears as a thin crest. The postaxial (medial) margin is short and half-round or concave, being thicker in lateral view. This results in a curved shape of the humerus and a wing-shaped cross section. The deltopectoral crest is very massive extending from the top of the proximal head to the anterior shaft region. At the beginning of the proximal shaft, the humerus has at the postaxial side a prominent edge or process, which is visible in dorsal and ventral view (Figs. 15A–15F, 20A). A latissimus dorsi insertion is only visible in the left humerus (Fig. 15E). The rounded distal end has a distinct ectepicondylar groove and an entepicondylar foramen. However, the latter is only in the right humerus visible (Figs. 15A, 15F).

One ulna is preserved, situated between the pubis and the axis on slab K (Figs. 5A, 16A). The ulna is broad and very flat, likely additionally compacted. The slightly curved proximal end is wider and has a long straight articulation facet to the humerus. The shaft is narrow with a straight postaxial margin and a slightly concave preaxial margin. The distal end is divided into two straight parts of which one is articulated once to the ulnare. Another long bone element is lying on the other side of the pubis on slab D (Figs. 5A, 16B). Although it is shorter than the ulna (Table 1), it is too long and massive to represent a metacarpal element. It is here interpreted as a radius. It has a convex proximal head; a constricted shaft and the distal end is wider than the shaft but not as wide as the proximal head. A large round-oval element, lying close to the neural spine of dorsal vertebra 1 (Figs. 5A, 16C), is interpreted as intermedium. It is a large element with a slightly concave dorsal margin.

The femur is straight and very slender (Figs. 5, 15G, 15H). The proximal head has a middle crest accompanied by two flanges of which the postaxial side is longer, broader, and convex curved (trochanter) when compared to the preaxial flange. The dorsal side of the proximal head is flat to slightly convex. The proximal and distal ends are not twisted and of equal width. The shaft is constricted appearing extremely slender compared to the broad shaft of the humerus. In cross section the femur is round-oval. The distal end is divided into two condyles.

Three tarsal ossifications are associated with the femur (Fig. 5A): astragalus, calcaneus and a third, possibly distal tarsal. The largest element is interpreted as the astragalus (Fig. 16D), with a round-oval shape and a constricted (*i.e*., concave) dorsal margin. The second largest element is the calcaneus, which is heart-shaped (Fig. 16E). The last element is smallest and roundish (Fig. 16E). In association with the femur and the tarsals are at least four metatarsalia (Figs. 5, 16I), which likely represent the 1^st^ to 4^th^ or 2^nd^ to 5^th^ toe, with the 1^st^ or 5^th^ small element lost or covered. They are of the typical hour glass shape with the two smaller elements being more rectangular. Associated with the metatarsalia are two phalanges, which are short, nearly rectangular and with a constricted shaft (Figs. 5A, 16I). Spread over the slabs are at least three more possible metatarsals or metacarpals (Figs. 5, 16F, 16G, 16J, 16K) and phalanges (Figs. 5, 16H).

The humerus-femur ratio is 1.12. The femur is only 2.5 cm shorter but it is much less wide and less massive than the humerus. In strong contrast is also the broad wing-shaped cross section of the humerus when compared to the round-oval cross section of the femur, clearly indicating different biomechanical usages. The humerus-ulna ratio is 2.09 and both elements share the flat and broad shape.

**Body size of UMO 1000 and maximal recorded size of *N. mirabilis***

The preserved strings of articulated and isolated vertebrae of the neck, trunk, sacral, and anterior tail region results in a length of 212 cm (Fig. 19). As described above, there is a clear morphological gap between the last preserved dorsal (d20; v39) and the sacral vertebrae, indicating the loss of the posterior dorsals. There are only very few complete skeletons of *Nothosaurus* to address the exact number of dorsal vertebrae. With 19 or 20 dorsals (Schröder, 1914) the stratigraphic older *N. marchicus* documents the plesiomorphic condition for *Nothosaurus* (Rieppel & Lin, 1995). *Lariosaurus* has between 20 and 24 (Rieppel, 1998) and *Ceresiosaurus* has 26 dorsal vertebrae (Hänni, 2004). The *Nothosaurus yangjuanensis* (*rostellatus*) from China has 24 dorsal vertebrae (Shang, 2006). The complete skeleton of the large sized and contemporaneously living *N. giganteus* shows 26 dorsal vertebrae (Peyer, 1939). *Simosaurus* has the most elongated trunk region with about 32 or more dorsal vertebrae (Rieppel, 1994). However, in different taxa of the specimen rich pachypleurosaurs, the number of vertebrae is variable between individuals of the same taxon (Rieppel & Lin, 1995; Klein et al., 2022). Meyer (1847–1855) speculated for UMO 1000 that about 8 to 10 dorsal vertebrae had been lost. Considering the number of other nothosaur taxa and the size difference between the last preserved dorsal (d20) and the–what we interpret as the–first sacral vertebrae (Table 1), we estimated approximately six dorsals to be lost. This would correspond with a length of about ~23.5 cm.

According to Meyer (1847–1855), the atlas preserved with UMO 1000 fits to a medium sized skull of *Nothosaurus mirabilis* of about 32 cm length, an estimation with which we agree. We further conservatively estimate between 20 to 50 cm for the missing posterior tail, resulting in a total body length for UMO 1000 of about at least 290 to 320 cm (Fig. 19). Our estimate of the total body length does not consider the slight bending of some parts of the vertebrae strings (slab C–E) or potentially additionally lost posterior dorsals, sacrals and anterior caudals. Münster (1834) estimated the total body length of the incomplete skeleton to be about 10 feet, which is ~305 cm. Meyer (1847–1855) calculated a lower total body length of 254.2 cm.

In our estimation the skull approximately makes about 10% of the entire body length (or skull:body length ratio is 0.1). This is contrary to Meyer (1847–1855), who concluded that the skull makes the 8^th^ part of the entire body length based on his body length calculation. Numerous skulls had been assigned to *Nothosaurus mirabilis*, of which some had been found at the same locality as the postcranial skeleton. The largest skull of *N. mirabilis* measures 46 cm (Rieppel & Wild, 1996), which would, according to our calculation, result in a total body length of about 460 cm.

### Ontogenetic stage of UMO 1000

Besides the above calculated total body length, there is some additional evidence that UMO 1000 did not represent a fully grown individual. Nearly all vertebrae are complete, meaning that centrum and neural arch are still articulated. However, in dorsals 13 to 20, centra and neural archs are separated by a distinct space of about over 0.5 cm (Figs. 9, 10). This could indicate a less firm ossification in this area. A weak connection between neural arch and centrum is typical of eosauropterygians, irrespective of their ontogenetic stage (*e.g*., Rieppel, 2000; Sander et al., 2014). Usually, centrum and neural arch of Eosauropterygia from the Germanic Basin are disconnected and found isolated (Rieppel, 2000; Klein, 2012; Klein et al., 2015). Brochu (2006) described a distinct caudal to cranial pattern of ossification along the vertebral column in modern crocodiles as a size independent criterion of maturity. Rieppel (2000: 69) observed that in nothosaurs ‘the dissociation of the neural arch from the centrum is less frequent in the cervical and sacral region than in the dorsal region’. Also, in pachypleurosaurs and *Nothosaurus marchicus*, the state of vertebral fusion along the column, *i.e*., neurocentral suture closure, is an indicator for skeletal maturity (Klein, 2012; Klein et al., 2015). Thus, the weak connection along the neurocentral suture of the posterior dorsals in UMO 1000 likely indicates a not fully ossified vertebral column and thus a not yet fully grown individual. Further on, this character supports the direction of ossification in the neck and the trunk column observed before by Rieppel (2000) from anterior to posterior, since the cervicals and anterior dorsals are more firmly fused than the preserved part of the posterior trunk region. The preserved caudal vertebrae are also well articulated. However, taphonomy can also influence the degree of ‘articulation of vertebrae’.

UMO 1000 further revealed a regular distance between centra of the neck and dorsals (*i.e*., intercentral space or intervertebral cartilage (Zwischenknorpel; Meyer, 1847–1855). In the neck, this space is about 0.5 cm wide, in the dorsals the space is less regular wide (likely due to preservation) but still distinct. It seems to narrow down in the posterior dorsals (Figs. 7–10). Because of the lateral preservation of UMO 1000, which is so far unique for *Nothosaurus*, it cannot be clarified whether this is typical of the genus *Nothosaurus*, or a particularity of UMO 1000, or if it is caused by the not fully adult stage of UMO 1000.

The massive compaction, *i.e*., flattening of some dorsal and caudal ribs could be related to incompletely ossified elements as well, or to special taphonomic processes (see below). Beside these indications of a semiadult status of UMO 1000, the skeleton is in general well ossified and developed.

## Discussion

### Comments on morphology

As typical for Sauropterygia, the postcranium of *N. mirabilis*, based on UMO 1000, shows a mixture of plesiomorphic (*e.g*., humerus morphology when compared in the course of aquatic adaptation to the simplified humerus morphology of *Pistosaurus*) and advanced features (high neural spines; large intercentral spaces; reduced humeral cortex).

#### Observations on the vertebral column

A detailed morphological comparison of *Nothosaurus* species can be found in the works of Peyer (1939), Rieppel (1994), Rieppel & Wild (1996), and Rieppel (2001). In the following we discuss only new aspects. However, comparison is anyway hampered, by the rareness of articulated or associated material and its different preservation. Pachypleurosaurs, of which high individual numbers of complete skeletons are known, show growth related morphological changes during ontogeny, depict distinct sexual dimorphism, and a well-documented generally high individual variation (*e.g*., Rieppel, 1989; Sander, 1989; Lin & Rieppel, 1998; Cheng et al., 2009; Klein et al., 2022). Likely, the same also applies to nothosaurs but complete skeletons are too rare to proof this view. Further on, eosauropterygian taxa are usually defined solely by skull characters or by a combination of characters (Klein et al., 2022) due to the similarities in the postcranial morphology, which result from secondary adaptation. In any case, one has to be careful not to overrate single features as was already pointed out by Geissler (1895).

The number of 19 cervical vertebrae seems to be rather constant in *N. marchicus, N. mirabilis*, and *N. giganteus*, while *N. jagisteus* has 24 cervicals. However, of each taxon only one articulated neck region is known and the exact number of vertebrae in each body region could be subject to intraspecific variation, as in pachypleurosaurs. The number of dorsals in nothosaurs is discussed above. The dorsal vertebrae of the trunk column of all nothosauroids show additional articulation facets called zygosphene-zygantrum (Rieppel, 1994). *Simosaurus* has additionally infraprezygapophyses and infrapostzygapophyses in the cervical and dorsal vertebrae (Rieppel, 1994). *Nothosaurus mirabilis* is unique among nothosauroids in having a supersized zygosphene-zygantrum articulation along its anterior dorsals (Figs. 8–10, 22), whereas the zygosphene-zygantrum articulation is normally developed in the neck. The neural spines of the posterior cervicals and dorsals of *Nothosaurus mirabilis* are constantly higher than in any other eosauropterygian as was pointed out several times before (summarized in Rieppel (2000)). Low dorsal neural spines are regarded as plesiomorphic in nothosaurs (Rieppel & Wild, 1996). In addition, the height of the dorsals of *N. mirabilis* increases from the anterior to the mid-dorsal region and decreases then again. The anterior neck, the sacral and the tail spines are low. The height of the neural spines of the dorsals of *N. marchicus, N. jagisteus*, and *N. giganteus* is constantly low or does not that distinctly increase and decrease, respectively. However, again, comparison is limited due to the rareness of complete skeletons.

The vertebral column of UMO 1000 reveals an additional specialty not observed in any other nothosaur, which however, could also be simply the result of its unique lateral preservation. Large spaces between the centra of UMO 1000 indicate large intervertebral cartilage discs, mainly in the neck region but also along the preserved trunk region. These are even larger when considering compaction or shift. Peyer (1939) did not explicitly mention intervertebral spaces in *Nothosaurus giganteus* (formerly *Paranothosaurus amsleri*) but from his figures and from the original skeleton (on exhibition at PIMUZ) spaces between the trunk centra are obvious in *N. giganteus* as well, although the skeleton was compacted during fossilisation, too. Smaller eosauropterygians such as pachypleurosaurs, *N. marchicus* (Schröder, 1914) and *N. jagisteus* (Rieppel, 2001) or *Pistosaurus* (Geissler, 1895) do not show such extensive intervertebral spaces. Aside all this, the vertebrae of *N. mirabilis* are in general more slender when compared to those of *N. giganteus* (which are nearly pachyostotic) and are even more slender when compared to those of *Pistosaurus* (Geissler, 1895; Sues, 1987). This slenderness is also supported by the high pedicles/pillars on which the neural arches stand on the centra. Contrarily, the neural arches of *N. giganteus* lie broadly on the centra. The transversal processes of the dorsals are much stronger and clearly more projected in *N. mirabilis*, reaching laterally far beyond the zygapophyses, when compared to those of *N. giganteus*. On the contrary, the neural arches, *i.e*., zygapophyses are in *N. mirabilis* not as broad as in *N. giganteus*.

UMO 1000 presents a–for an eosauropterygian–short neck and tail (note that the latter is only reconstructed and could have been longer than in our interpretation), whereas the trunk is relatively elongated (Fig. 19). *N. mirabilis* shares these body proportions with *N. giganteus* (Peyer, 1939) and *Simosaurus* (Rieppel, 1994) but differs from the smaller taxa *N. marchicus* and N. *jagisteus*. Meyer (1847–1855) already recognized the slight bending of the neck and that the anterior trunk region formed a slight arch in UMO 1000 (Fig. 19). However, this position could be a result of taphonomy.

The *Nothosaurus yangjuanensis* and *N. youngi* from China differ in their vertebral morphology and size from *N. mirabilis* (*e.g*., Shang, 2006; Ji et al., 2014). Some nothosaur postcranial bones and vertebrae from other European localities (summarized in Marquez-Aliaga et al. (2017)) and Israel (Rieppel, Mazin & Tchernov, 1999) are similar to UMO 1000 (as well as to other taxa from the Germanic Basin) but this material is in general too incomplete to be compared in detail.

#### Humerus morphology and histology

The humerus of Sauropterygia is important since its morphology and histology often allows at least a rough assignment to a major group level (Klein, 2010). Nothosaur humeri generally share a very flat and curved shape, a separation into a proximal, midshaft, and distal area, and have a characteristic wing-shaped cross section (Klein, 2010; Krahl, Klein & Sander, 2013; Klein et al., 2016). However, preservation, ontogenetic and individual variation, sexual dimorphism and the rareness of humeri associated with skulls often hamper their exact taxonomic assignment. Hence, UMO 1000 provides the rare opportunity now to assign a certain humerus type to a specific taxon. The humerus of *N. mirabilis* differs from all other nothosaur humeri by its extremely broad unconstricted shaft, the very prominent edge or process at the beginning of the proximal postaxial shaft margin, and by a thin but broad crest that forms the preaxial half. In addition, the humerus of *N. mirabilis* appears edged and displays more morphological features when compared, for example, to humeri of similar size of *N. giganteus*, which is smooth and has a more simple morphology (Peyer, 1939; Rieppel & Wild, 1996; SMNS 17822/3) (Fig. 20). The deltopectoral crest is less pronounced in *N. giganteus* and the entire proximal head is less differentiated (Fig. 20).

With this knowledge, an assignment of some of the humeri studied by Klein et al. (2016) to *N. mirabilis* or *N. giganteus*, respectively, is now possible (Fig. 21). Klein et al. (2016) documented very thin cortices and very thick cortices in large humeri of nothosaurs, but a further interpretation was not possible. The now possible taxonomic assignment reveals that the very thin cortices are not primarily related to taxonomy (Fig. 21). These thin cortices are typical of both large nothosaurs, however, cortices of *Nothosaurus mirabilis* humeri are tendentially thinner than cortices of *N. giganteus* (Fig. 21). In addition, decreasing cortex thickness seems to be related to older individuals (or larger size), because most of the very large humeri show the thinnest cortices. However, few exceptions exist (Fig. 21; Klein et al., 2016). Unfortunately, some humeri depicting a very thick cortex are too incompletely and/or poorly preserved to assign them unequivocally to *N. mirabilis* or *N. giganteus*. However, it seems as if *N. mirabilis* and *N. giganteus* each have an example of such an extreme thick cortex as well (Fig. 21). Small UM nothosaur taxa in general tend to develop thick cortices (Klein et al., 2016). Hence, the high humeral microanatomical diversity in large nothosaurs is a result of developmental plasticity, *i.e*., environmental influence (coastal or shallow marine *vs.* open sea). It might be further related to large body size (humerus length >25 cm) and adaptation to sustained swimming (see below). The very compact humeri belong to medium-sized individuals (around 20 cm humerus length) but this can be a sampling bias.

Despite its size, the humerus of *N. jagisteus* (Rieppel, 2001) is more slender compared to *N. mirabilis* and has a constricted shaft. However, it shares with the latter the angled proximal head with a well-developed deltopectoral crest, the process at the postaxial proximal shaft, the short curved postaxial and long straight preaxial margin (Fig. 20). The type skeleton of *N. jagisteus* thus might represent a juvenile individual of *N. mirabilis* and would be a junior synonym of this taxon. However, Rieppel (2001) argued against this interpretation. A detailed morphological re-study of the entire skeleton of *N. jagisteus*, maybe aided by micro-CT data, is necessary to clarify its status.

#### Hindlimb and pelvic elements

All nothosaurs share a straight or slightly s-shaped and slender femur when compared to the humerus. The proximal femur head is usually prominent whereas the shaft is constricted, round oval and the distal end is flat, differentiated into two more or less prominent condyles. The femur of UMO 1000 is straight but otherwise typical nothosaurian. It is shorter than the humerus (Table 1). The ilium of UMO 1000 also displays the typical nothosaur morphology with a weakly set off iliac blade and a rather simple morphology when compared to other eosauropterygians (see Rieppel, 1994: fig. 27, 28, 50). Contrary to smaller nothosaur taxa and *Simosaurus*, the ischium of *N. mirabilis* is comparatively symmetrical and with a clearly set off and long constricted shaft. The ischia of *N. mirabilis* and *N. giganteus* are quite similar. The same is true for the pubis; both large nothosaur taxa share a rather angular pubis with a deep slit and only a slightly set off prepubis.

### Comments on locomotion of *Nothosaurus mirabilis*

Braun & Reif (1985) interpreted *Nothosaurus* as an anguilliform swimmer, *i.e*., moving by propulsion by lateral undulation. However, the relatively short *Nothosaurus* tail does not show the morphology of a distinct swimming tail, such as elongation, high neural spines and chevrons, lateral compaction (*e.g*., Peyer, 1939; this study). Hence, the tail of *Nothosaurus*, could only marginally contribute to propulsion by undulating. This is contrary to many other marine reptiles such as mesosaurs, thalattosaurs, pachypleurosaurs, and choristoderes or modern the marine iguana, which all have a strong swimming tail (summarized in Houssaye (2013)).

Rieppel (1994: 35–36) noted for *Simosaurus*: ‘Interlocking of pre- and postzygapophyses and infrapre- and infrapostzygapophyses [only present in *Simosaurus*] with almost vertically oriented articular surfaces effectively reduces the potential for lateral undulation in the posterior part of the axial skeleton, whereas the neck and anterior trunk region with more horizontally oriented pre- and postzygapophyses would preserve their potential for extensive lateral bending during prey capture’. UMO 1000 has horizontally oriented zygapophyses throughout its preserved vertebral column and large intercentral spaces in the neck and anterior trunk region allowing for lateral movements (*i.e*., undulation). The neural spines were firmly connected *via* the zygosphene-zygantrum articulation and are thus rigid. The exceptional height of the neural spines and their additional stiffening indicate an extremely strong *musculus longissimus/latissimus dorsi*, which would further contribute to lateral undulation. This means that *N. mirabilis* used its trunk region–instead of a swimming tail–as main propulsion element during swimming by lateral undulation.

Muscle reconstructions and morphological observations of the pectoral girdle led other authors to the conclusion that Nothosauroidea had evolved paraxial locomotion (Watson, 1924; Huene, 1944; Carroll & Gaskill, 1985) with the forelimbs employed in a ‘rowing flight’, combining lift- and drag-based elements of propulsion. The use of the forelimbs for propulsion was supported by trace fossils interpreted as swimming tracks possibly formed by a large nothosaur (Zhang et al., 2014) and by morphological observations such as wing-shaped humeral cross section (Klein et al., 2015; Krahl, 2021). Thewissen & Taylor (2007) argued for a sculling type of locomotion in nothosaurs assuming a stiff body with weak and short tail, contrary to their stout and broad forearms. As is evident from UMO 1000 and the few other nothosaur skeletons known, the humerus of nothosaurs is longer than the femur, which put them into the ‘M4 category’ of aquatic adaptation of Motani & Vermeij (2021). Further on, the humerus in nothosaurs is much more massive and broader when compared to the femur, implying that the humerus played a much more active role in locomotion and propulsion than the femur. The hindlimbs likely were used only for maneuvering. Following all these former observations together with the new information based on UMO 1000, a combination of lateral undulation and paraxial propulsion seems applicable for *N. mirabilis*. It is conceivable that different kinds of locomotion were used depending on speed: Propelling with the forelimbs was *e.g*., used at slow swimming and lateral undulation for fast swimming.

Differences in locomotion are also supported by different feeding strategies. The more gracile skull and jaw with the exceptional frontal fangs of *N. mirabilis* (*i.e*., SMNS 18690) indicate a mainly piscivorous diet whereas *N. giganteus* with its massive posteriorly extended lower jaw seemed to have had stronger bite forces, likely to subdue larger prey such as other reptiles. *Simosaurus* that is comparable in size to *N. mirabilis* had a rounded skull with a durophagous dentition and was probably able to catch moderately hard-shelled prey such as hard-scaled fishes or ammonoids with a quick snapping bite (Rieppel, 1994).

It is thus, quite clear, based on morphological differences in vertebrae, humeri (including histology and microanatomy; Klein & Griebeler, 2016; Klein et al., 2016, this study) as well as in skull and dentition that the three large nothosauroids, *N. mirabilis, N. giganteus*, and *Simosaurus*, followed each a different trait of locomotion and hunting and feeding strategy. In consequence, swimming abilities and styles likely were also different among these taxa. Similar differentiations were already claimed for other marine reptile taxa by Massare (1988). *N. mirabilis* and *N. giganteus* tendentially share the reduction of the humeral cortex with a distinct decrease in bone mass, which is typical for fast, possibly sustained open marine swimmers (Ricqlès & Buffrénil, 2001). However, based on morphology, microanatomy and the various localities were *Nothosaurus* spp. can been found, they likely were not highly specialized to a certain environment but seemed to have managed well in different habitats. Conversely, *Simosaurus* shows tendentially osteosclerotic humeri and femora (Klein & Griebeler, 2016), appropriate for an animal living and foraging in shallow marine environments. However, a detailed study and interpretation of biomechanics and functional questions as well as a thorough analysis of the swimming style is beyond the scope of this article.

### Taphonomy

As described above in detail, UMO 1000 underwent an interesting pre-burial history as is evidenced by the pattern of articulation of neck and most of the trunk vertebral column and disarticulation of the rest of the skeleton, as well as by the strange position of strings of vertebrae and shift of other elements as by movements of elements from one body half to the other (left and right body side elements) (Figs. 5, 6). The skull and most of the lower jaw are lost, the complete shoulder girdle, some parts of the posterior tail, the left half of the pelvic girdle and most elements of the zeugo- and autopodium. A total of 75% of the bones of the stylopodium are preserved. The predominately lateral position of the vertebral column itself is a rare phenomenon among marine reptiles, which are usually preserved in dorsal or ventral position. Pre-burial movements are indicated by the nearly 180° turned strings of the anterior cervical and anterior caudal vertebrae, by the caudal shift of both humeri whereas the preserved pelvic bones have shifted cranially. The femur, which lies close to the sacral region, shows an orientation as observed in other articulated nothosaur specimens (*i.e*., *N. marchicus*, Schröder, 1914; *N. jagisteus*, Rieppel, 2001) with the distal end pointing cranially, suggesting a kind of crouched position. The latter however, could well be the result of muscle relaxing. The loss of smaller bones such as posterior tail vertebrae or most autopodial elements can be explained by slight water movements or scavenging by small carrion feeder. Also, the loss of the skull and lower jaw is likely due to water movements since the skull is the first element in a drifting and decaying tetrapod to disarticulate from the rest of the skeleton (Weigelt, 1927). The absence of heavy elements such as the shoulder girdle and parts of the pelvic girdle, the shift of humeri and the remaining pelvic girdle elements might be the result of the impact by larger scavengers or stronger water movements. It is conceivable that the skeleton was anchored somehow and it was not possible for scavengers to remove big chunks of the body completely. After burial, the vertebral column underwent lateral compaction and the left humerus and some of the dorsal and caudal ribs were heavily dorso-ventrally flattened.

## Conclusions

- An updated sedimentological profile is figured for the historical quarries in the vicinity of Bayreuth with an assessment of the origin of UMO 1000, the holotype of *Nothosaurus mirabilis*.
- A detailed overview about historical activities around the UM of Bayreuth and the history of UMO 1000 is given to clarify some anecdotal knowledge.
- The historical postcranial skeleton, the holotype of *Nothosaurus mirabilis*, is after further preparation and remounting re-described and figured in detail for the first time. The description provides morphological characters for future phylogenetic analyses.
- *N. mirabilis* shows a mixture of plesiomorphic (*e.g*., humerus morphology) and advanced features (high neural spines; reduced humeral cortex).
- Aside its heigh neural spines in dorsal vertebrae, the vertebral column of *Nothosaurus mirabilis* is unique due to the supersized zygosphene-zygantrum articulation of dorsals and large intercentral spaces all along the neck and trunk.
- The humerus of *Nothosaurus mirabilis* is very characteristic. It can now be clearly distinguished from that of *Nothosaurus giganteus*. This allows further assigning of some isolated humeri to the two taxa.
- Humeral microanatomy described in a former study is with the new taxonomical knowledge revisited. It documents that reduced humeral cortices are related to size and developmental plasticity or environment and not to taxonomy.
- Locomotion of *Nothosaurus mirabilis* is only preliminary discussed but morphological characters such as stiffening of upper dorsal vertebrae and flexibility between centra, humerus morphology and microanatomy clearly indicate complex and likely speed depending different kinds of locomotion and for sure the ability of fast, maybe even sustained swimming.
- All large nothosaurs (*N. mirabilis*, *N. giganteus*, *Simosaurus*) show differences in morphology and aquatic adaptations resulting in different locomotion modes and likely feeding strategies. Future research should focus on biomechanics and functional questions and on a detailed analysis of the swimming style in *Nothosaurus*.
- The lateral position and the degree of articulation of UMO 1000 with strings of vertebrae and appendicular elements turned and shifted is quite unique. It indicates fast burial but also anchorage and likely scavenging of large predators on the body.

## Institutional abbreviations

MfN/MB.R: Museum für Naturkunde, Berlin
MHI: Muschelkalkmuseum Hagdorn, Ingelfingen
SMNS: Staatliches Museum für Naturkunde, Stuttgart
SFM: Senckenberg Forschungsinstitut und Naturmuseum, Frankfurt
UMO: Urwelt-Museum Oberfranken, Bayreuth; all Germany

## Figure Abbreviations

as, astragalus; at, atlas; ax, axis; acau, anterior caudal vertebra; c, cervical vertebra; ca, carpale; cl, calcaneus; cr, cervical rib; cau, caudal; caur, caudal rib; dr, dorsal rib; g, gastralium; h, haemapophyses; hu, humerus; il, ilium; is, ischium; mtc, metacarpal; pcau, posterior caudal; ph, phalange; pu, pubis; sa, sacral vertebra; sar, sacral rib; ul, ulna.

## Additional Information and Declarations

### Data Availability

The following information was supplied regarding data availability: The specimen number UMO 1000 is housed at the Urweltmuseum Bayreuth, Oberfranken, Germany.

## References

[ref-100] Alafont LS (1992). Notosaurios y Placodontos (Reptilia) del Triásico Medio de Bienservida – Villarrodrigo. Instituto de Estudios albac-etenses de la Excma. Diputación de Albacete.

[ref-1] Agassiz L (1833–1844). Recherches sur les poissons fossiles.

[ref-2] Bachmann GH, Beutler G, Hagdorn H (1999). Muschelkalk und Keuper am Autobahndreieck Bayreuth/Kulmbach, Nordost-Bayern. Hallesches Jahrbuch für Geowissenschaften B.

[ref-3] Baur G (1889). Palaeohatteria Credner, and the Proganosauria. American Journal of Science.

[ref-4] Blanchard R (1889). Bulletin de la Société zoologique de France.

[ref-5] Blanchard R, Maehrenthal F, Stiles CW (1905). Règles internationales de la Nomenclature Zoologique adoptées par les Congrès Internationaux de Zoologie. International Rules of Zoological Nomenclature. Internationale Regeln der Zoologischen Nomenklatur. Paris Rudeval.

[ref-6] Braun CWF (1840). Verzeichnis der in der Kreissammlung von Bayreuth befindlichen Petrefakten.

[ref-7] Braun J, Reif WE (1985). A survey of aquatic locomotion in fishes and tetrapods. Neues Jahrbuch für Geologie und Paläontologie, Abhandlungen.

[ref-99] Brochu CA (2006). A new miniature horned crocodile from the Quaternary of Aldabra Atoll, western Indian Ocean. – Copeia.

[ref-8] Bronn HG (1835–1838). Lethaea Geognostica oder Abbildungen und Beschreibungen der für die Gebirgsbildungen bezeichnendsten Versteinerungen. Erster Band das Übergangs- bis Oolithen-Gebirge enthaltend. Zweiter Band das Kreide- und Molassen-Gebirge enthaltend.

[ref-9] Carroll RL, Gaskill P (1985). The nothosaur *Pachypleurosaurus* and the origin of plesiosaurs. Philosophical Transactions of the Royal Society of London Series B: Biological Sciences.

[ref-10] Cheng Y-N, Holmes R, Wu X-Ch, Alfonso N (2009). Sexual dimorphism and life history of *Keichousaurus hui* (Reptilia: Sauropterygia). Journal of Vertebrate Paleontology.

[ref-11] Conybeare WD (1824). On the discovery of an almost perfect skeleton of the Plesiosaurus. Transactions of the Geological Society of London.

[ref-12] Cuvier G (1824). Recherches sur les ossemens fossils où l’ on rétablit les charactères de plusieurs animaux don’t les révolutions du globe ont détruit les espèces, nouvelle edition, vol. 5, 2nd part, 33 pls.

[ref-13] Diedrich CG (2012). The Middle Triassic marine reptile biodiversity in the Germanic Basin, in the centre of the Pangaean world. Central European Journal of Geosciences.

[ref-14] Diener W, Zapf H (2004). Wege und Verbleib Bayreuther paläontologischer und mineralogischer Sammlungen. Berichte der Naturwissenschaftlichen Gesellschaft Bayreuth.

[ref-95] Edinger T (1921). Über *Nothosaurus* II. Zur Gaumenfrage. Senckenbergiana.

[ref-15] Emmert U (1977). Geologische Karte von Bayern 1:25.000. Erläuterungen zu Blatt 6035 Bayreuth.

[ref-16] Emmert U, Stettner G (1995). Geologische Karte von Bayern 1:25.000. Erläuterungen zu Blatt 6036 Weidenberg.

[ref-101] Frosch H (1923). Die Ceratiten des Bayreuther Muschelkalkes. – Jahresberichte und Mitteilungen des oberrheinischen geologischen Vereins. Neue Folge.

[ref-19] Furrer H (1995). The Prosanto Formation, a marine Middle Triassic Fossil-Lagerstätte near Davos (Canton Graubünden, Eastern Swiss Alps). Eclogae Geologicae Helvetiae.

[ref-20] Geissler G (1895). Über neue Saurier-Funde aus dem Muschelkalk von Bayreuth. Zeitschrift der Deutschen Geologischen Gesellschaft, Band.

[ref-21] Gevers TW (1926). Der Muschelkalk am Nordwestrande der Böhmischen Masse. Neues Jahrbuch für Mineralogie, Geologie und Paläontologie, Beilage-Band B, Stuttgart.

[ref-22] Geyer G, Friedlein V (2020). Die Randfazies des Muschelkalks in Nord-Bayern. In: Deutsche Stratigraphische Kommission (Hrsg.; Koordination und Redaktion, für die Subkommission Perm-Trias): Stratigraphie von Deutschland XIII. Muschelkalk. Schriftenreihe Deutsche Gesellschaft für Geowissenschaften.

[ref-25] Hagdorn H (2020). Biostratigraphie der Muschelkalk-Cephalopoden. In: Deutsche Stratigraphische Kommission (Hrsg.; Koordination und Redaktion: Hagdorn, H., Simon, T., für die Subkommission Perm-Trias): Stratigraphie von Deutschland XIII. Muschelkalk.

[ref-26] Hagdorn H, Rieppel O (1999). Stratigraphy of marine reptiles in the Triassic of Central Europe. Zentralblatt für Geologie und Paläontologie Teil I.

[ref-27] Hagdorn H, Simon T, Dittrich D, Ernst R, Farrenschon J, Freudenberger W, Geyer G, Kramm E, Vath U (2020). Lithostratigraphie der Oberer-Muschelkalk-Subgruppe.

[ref-23] Hänni K (2004). Die Gattung Ceresiosaurus. Ceresiosaurus calgagnii Peyer und Ceresiosaurus lanzi n.sp. (Lariosauridae, Sauropterygia).

[ref-28] Houssaye A (2013). Bone histology of aquatic reptiles: what does it tell us about secondary adaptation to an aquatic life?. Biological Journal of the Linnean Society.

[ref-29] Huene FV (1933). Die Placodontier. 4. Zur Lebensweise und Verwandtschaft von Placodus. Abhandlungen der Senckenbergischen Naturforschenden Gesellschaft.

[ref-30] Huene F (1944). Ein beachtenswerter Humerus aus unterstem Muschelkalk und seine Bedeutung. Neues Jahrbuch für Mineralogie, Monatshefte Abt B.

[ref-31] Huene EV (1949). Studie über die Umwandlung des Landfußes in den Schwimmfuß bei Sauropterygiern und Placodontiern, gezeigt an der Vorderextremität. Neues Jahrbuch für Mineralogie, Geologie und Paläontologie, Abhandlungen.

[ref-32] Jiang D-Y, Motani R, Hao W, Rieppel O, Sun Y, Tintori A, Sun Z, Schmitz L (2009). Biodiversity and sequence of the Middle Triassic Panxian Marine Reptile Fauna, Guizhou Province, China. Acta Geologica Sinica–English Edition.

[ref-33] Ji C, Jiang D-Y, Rieppel O, Motani R, Tintori A, Sun Z-Y (2014). A new specimen of Nothosaurus youngi from the Middle Triassic of Guizhou, China. Journal of Vertebrate Paleontology.

[ref-35] Klein N (2010). Long bone histology of Sauropterygia from the Lower Muschelkalk of the Germanic Basin provides unexpected implications for phylogeny. PLOS ONE.

[ref-36] Klein N (2012). Postcranial morphology and growth of the pachypleurosaur *Anarosaurus heterodontus* (Sauropterygia) from the Lower Muschelkalk of Winterswijk, The Netherlands. Paläontologische Zeitschrift.

[ref-37] Klein N, Griebeler EM (2016). Bone histology, microanatomy, and growth of the nothosaurid *Simosaurus gaillardoti* (Sauropterygia) from the Upper Muschelkalk of southern Germany/Baden-Württemberg. Comptes Rendus Palevol.

[ref-39] Klein N, Voeten DFAE, Lankamp J, Bleeker R, Sichelschmidt OJ, Liebrand M, Nieweg DC, Sander PM (2015). Postcranial material of *Nothosaurus marchicus* from the Lower Muschelkalk (Anisian) of Winterswijk, The Netherlands, with remarks on swimming styles and taphonomy. Paläontologische Zeitschrift.

[ref-40] Klein N, Sander PM, Krahl A, Scheyer TM, Houssaye A (2016). Diverse aquatic adaptations in *Nothosaurus* spp. (Sauropterygia)–inferences from humeral histology and microanatomy. PLOS ONE.

[ref-41] Klein N, Canoville A, Houssaye A (2019). Microstructure of vertebrae, ribs, and gastralia of Triassic sauropterygians–new insights into the microanatomical processes involved in aquatic adaptations of marine reptiles. The Anatomical Record.

[ref-42] Klein N, Furrer H, Ehrbar I, Torres Ladeira M, Richter H, Scheyer TM (2022). A new pachypleurosaur from the early Ladinian Prosanto Formation in the Eastern Alps of Switzerland. Swiss Journal of Palaeontology.

[ref-43] Krahl A (2021). The locomotory apparatus and paraxial swimming in fossil and living marine reptiles: comparing Nothosauroidea, Plesiosauria, and Chelonioidea. Paläontologische Zeitschrift.

[ref-44] Krahl A, Klein N, Sander PM (2013). Evolutionary implications of the divergent long bone histologies of *Nothosaurus* and *Pistosaurus* (Sauropterygia, Triassic). BMC Evolutionary Biology.

[ref-34] Li Q, Liu J (2020). An Early Triassic sauropterygian and associated fauna from South China provide insights into Triassic ecosystem health. Communications Biology.

[ref-45] Lin K, Rieppel O (1998). Functional morphology and ontogeny of Keichousaurus hui (Reptilia, Sauropterygia).

[ref-46] Lin W-B, Jiang D-Y, Rieppel O, Motani R, Tintori A, Sun Z-Y, Zhou M (2021). *Panzhousaurus rotundirostris* Jiang et al., 2019 (Diapsida: Sauropterygia) and the recovery of the monophyly of Pachypleurosauridae. Journal of Vertebrate Paleontology.

[ref-47] Liu J, Hu S-X, Rieppel O, Jiang D-Y, Benton MJ, Kelley NP, Aitchison JC, Zhou C-Y, Wen W, Huang J-Y, Xie T, Lv T (2014). A gigantic nothosaur (Reptilia: Sauropterygia) from the Middle Triassic of SW China and its implication for the Triassic biotic recovery. Scientific Reports.

[ref-49] Marquez-Aliaga Ana, Klein N, Reolid M, Plasencia P, Villena JA, Martinez-Perez C (2017). An enigmatic marine reptile, *Hispaniasaurus cranioelongatus* (gen. et sp. nov.) with nothosauroid affinities from the Ladinian of the Iberian Range (Spain). Historical Biology.

[ref-48] Massare JA (1988). Swimming capabilities of Mesozoic marine reptiles: implications for method of predation. Paleobiology.

[ref-50] Meyer H v (1832). Palaeologica zur Geschichte der Erde und ihrer Geschöpfe.

[ref-51] Meyer H v (1833). Beiträge zur Petrefaktenkunde. Conchiosaurus clavatus, ein Saurus aus dem Muschelkalke von Bayreuth. Museum Senckenbergianum.

[ref-52] Meyer H v (1847–1855). Die Saurier des Muschelkalks mit Rücksicht auf die Saurier aus Buntem Sandstein und Keuper. Zur Fauna der Vorwelt, zweite Abtheilung.

[ref-56] Motani R, Vermeij GJ (2021). Ecophysiological steps of marine adaptation in extant and extinct non-avian tetrapods. Biological Reviews.

[ref-53] Müller H (1979). Bayreuth und die Paläontologie. Der Aufschluss.

[ref-54] Münster G (1830). Über einige ausgezeichnete fossile Fischzähne aus dem Muschelkalk bei Bayreuth.

[ref-55] Münster G (1834). Vorläufige Nachricht über einige neue Reptilien im Muschelkalke von Baiern. Neues Jahrbuch für Mineralogie, Geognosie, Geologie und Petrefaktenkunde.

[ref-57] Owen R (1858). Description of the skull and teeth of the Placodus laticeps Owen. With indications of other new species of Placodus, and evidence of the saurian nature of that genus. Philosophical Transactions of the Royal Society of London.

[ref-58] Owen R (1860). Palaeontology; or, a systematic summary of extinct animals and their geologic remains.

[ref-59] Peyer B (1939). Die Triasfauna der Tessiner Kalkalpen XIV. Paranothosaurus amsleri nov. gen. nov. spec. Abhandlungen der Schweizerischen Paläontologischen Gesellschaft.

[ref-60] Reis OM (1923a). Ausflug nach dem Bindlacher Berg mit den Muschelkalkbrüchen. Jahresberichte und Mitteilungen des oberrheinischen geologischen Vereins, Neue Folge.

[ref-61] Reis OM (1923b). Ausflug in die Triasablagerungen im unteren Steinachtal zwischen Laineck, Rodersberg und Döhlau am Oschenberg. Jahresberichte und Mitteilungen des oberrheinischen geologischen Vereins, Neue Folge.

[ref-62] Ricqlès A de, Buffrénil V de, Mazin J-M, Buffrénil Vde (2001). Bone histology, heterochronies and the return of tetrapods to life in water: where are we?. Secondary Adaptations of Tetrapods to Life in Water.

[ref-63] Rieppel O (1989). A new pachypleurosaur (Reptilia: Sauropterygia) from the Middle Triassic of Monte San Giorgio, Switzerland. Philosophical transactions of the Royal Society of London B.

[ref-64] Rieppel O (1994). Osteology of *Simosaurus gaillardoti* and the relationships of stem-group Sauropterygia. Fieldiana: Geology.

[ref-97] Rieppel O (1998). The status of the sauropterygian reptile genera *Ceresiosaurus*, *Lariosaurus* and *Silvestrosaurus* from the Middle Triassic of Europe. - Fieldiana (Geology) n.s..

[ref-65] Rieppel O, Wellnhofer P (2000). Sauropterygia I. Placodontia, Pachypleurosauria, Nothosauroidea, Pistosauroidea. Handbuch der Paläoherpetologie/encyclopedia of paleoherpetology, Teil 12A.

[ref-66] Rieppel O (2001). A new species of *Nothosaurus* (Reptilia: Sauropterygia) from the Upper Muschelkalk (Lower Ladinian) of southwestern Germany. Palaeontographica Abteilung A.

[ref-67] Rieppel O, Lin K (1995). Pachypleurosaurs (Reptilia: Sauropterygia) from the Lower Muschelkalk, and a review of the Pachypleurosauroidea. Fieldiana: Geology.

[ref-68] Rieppel O, Brinkmann P (1996). Case 2994. *Nothosaurus* Münster, 1834 (Reptilia, Sauropterygia): proposed precedence over *Conchiosaurus* Meyer [1833]. Bulletin of Zoological Nomenclature.

[ref-96] Rieppel O, Hagdorn H, Callaway JM, Nicholls EL (1997). Paleobiogeography of Middle Triassic Sauropterygia in Central and Western Europe. Ancient Marine Reptiles.

[ref-69] Rieppel O, Wild R (1996). A revision of the genus *Nothosaurus* (Reptilia, Sauropterygia) from the Germanic Triassic, with comments on the status of *Conchiosaurus clavatus*. Fieldiana (Geology), n.s.

[ref-70] Rieppel O, Sander MP, Storrs GW (1997). The skull of the pistosaur *Augustasaurus* from the Middle Triassic of Northwestern Nevada. Journal of Vertebrate Paleontology.

[ref-71] Rieppel O, Mazin JM, Tchernov E (1999). Sauropterygia from the Middle Triassic of Makhtesh Ramon, Negev, Israel. Fieldiana (Geology), n.s.

[ref-72] Röhl HJ, Schmid-Röhl A, Furrer H, Frimmel A, Oschmann W, Schwark L (2001). Microfacies, geochemistry and palaeoecology of the Middle Triassic Grenzbitumenzone from Monte San Giorgio (Canton Ticino, Switzerland). Geologia Insubria.

[ref-73] Sander MP (1989). The pachypleurosaurids (Reptilia: Nothosauria) from the Middle Triassic of Monte San Giorgio (Switzerland) with the description of a new species. Philosophical Transactions of the Royal Society of London B.

[ref-98] Sander PM, Klein N, Albers PCH, Bickelmann C, Winkelhorst H (2014). Postcranial morphology of a basal Pistosauroidea (Sauropterygia) from the Lower Muschelkalk of Winterswijk, The Netherlands. Paläontologische Zeitschrift.

[ref-74] Scheyer TM, Neuman AG, Brinkman DB (2019). A large marine eosauropterygian reptile with affinities to nothosauroid diapsids from the Early Triassic of British Columbia, Canada. Acta Palaeontologica Polonica.

[ref-75] Schröder H (1914). Wirbeltiere der Rüdersdorfer Trias. Abhandlungen der Königlich Preussischen Geologischen Landesanstalt. Neue Folge.

[ref-76] Shang Q-H (2006). A new species of *Nothosaurus* from the early Middle Triassic of Guizhou, China. Vertebrata PalAsiatica.

[ref-77] Shang Q, Wu XC, Li Ch (2020). A new Ladinian nothosauroid (Sauropterygia) from Fuyuan, Yunnan Province, China. Journal of Vertebrate Paleontology.

[ref-78] Spiekmann SNF, Scheyer TM (2019). A taxonomic revision of the genus Tanystropheus (Archosauromorpha, Tanystropheidae). Palaeontologia Electronica.

[ref-80] Storrs GW (1991). Anatomy and Relationships of *Corosaurus alcovensis* (Diapsida: Sauropterygia) and the Triassic Alcova Limestone of Wyoming. Bulletin of the Peabody Museum of Natural History Yale University.

[ref-79] Sues H-D (1987). Postcranial skeleton of *Pistosaurus* and interrelationships of the Sauropterygia (Diapsida). Zoological Journal of the Linnean Society.

[ref-81] Sun Z, Jiang D, Ji C, Hao W (2016). Integrated biochronology for Triassic marine vertebrate faunas of Guizhou Province, South China. Journal of Asian Earth Sciences.

[ref-82] Thewissen JGM, Taylor AM, Hall BK (2007). Aquatic adaptations in the limbs of amniotes. Fins into Limbs: Evolution, Development, and Transformation.

[ref-83] Tschanz K (1989). *Lariosaurus buzzii* n. sp. from the Middle Triassic of Monte San Giorgio (Switzerland) with comments on the classification of nothosaurs. Palaeontographica Abt A.

[ref-84] Voeten DFAE, Albers PCH, Klein N (2019). Nothosauroidea from the Vossenveld Formation and their relatives. Grondboor & Hamer (Staringia 16).

[ref-86] Watson DMS (1924). The elasmosaurid shoulder-girdle and fore-limb. Proceedings of the Zoological Society London.

[ref-87] Weigelt J (1927). Rezente Wirbeltierleichen und ihre paläobiologische Bedeutung.

[ref-88] Weiss GW (1937). Bayreuth als Stätte alter erdgeschichtlicher Entdeckungen.

[ref-89] Weiss GW (1954). Zur Frage der Muschelkalk-Obergrenze bei Bayreuth. Erlanger geologische Abhandlungen.

[ref-90] Weiss GW (1983). Bayreuth als Stätte alter erdgeschichtlicher Entdeckungen. Zum 150. Jubiläum des Graf-Münster-Gymnasiums Bayreuth.

[ref-91] Welzel E (1963). Stratigraphie der Ceratitenschichten im Gebiet Bayreuth-Kronach. Geologische Blätter für Nordost-Bayern.

[ref-92] Wild R (1972). Die Wirbeltierfaunen der fränkischen und südalpinen Mitteltrias (ein Vergleich). Zeitschrift der deutschen geologischen Gesellschaft.

[ref-93] Wild R (1988–1989). Erdwissenschaftliche Forschungen in Bayreuth–Ein historischer Überblick. Berichte der Naturwissenschaftlichen Gesellschaft Bayreuth.

[ref-94] Zhang Q, Wen W, Hu S, Benton MJ, Zhou C, Xie T, Lü T, Huang J, Choo B, Chen Z-Q, Liu J, Zhang Q (2014). Nothosaur foraging tracks from the Middle Triassic of southwestern China. Nature Communications.

